# Nucleosomes and IDRs suppress promiscuous GCN4 binding on minichromosomes

**DOI:** 10.1038/s41594-026-01864-x

**Published:** 2026-08-13

**Authors:** Zelin Wei, Oluwakemi E. Abiodun, Yick Hin Ling, Fijare Plous, Maryam Yamadi, Katherine Chen, Songgao Shan, Annie Huang, Carl Wu

**Affiliations:** 1 Department of Biology, Johns Hopkins University, Baltimore, MD USA; 2 Department of Biomedical Engineering, Johns Hopkins School of Medicine, Baltimore, MD USA; 3 Department of Molecular Biology and Genetics, Johns Hopkins School of Medicine, Baltimore, MD USA; 4 Present Address: Department of Biology, Emory University, Atlanta, GA USA; 5 Present Address: Center for Cell and Gene Therapy, Baylor College of Medicine, Houston, TX USA

**Keywords:** Nucleosomes, Single-molecule biophysics, DNA, Transcription

## Abstract

Eukaryotic sequence-specific transcription factors (TFs) must find their cognate DNA targets hidden in genomic chromatin amid an excess of nonspecific sequences and degenerate motifs. Although static TF interactions with nucleosomal targets have been elucidated, how TFs efficiently search for cognate sites within native gene-sized chromatin domains has been unclear. Here we used purified *Saccharomyces* *cerevisiae* *HIS3* minichromosomes and single-molecule imaging to compare association and dissociation kinetics of transcription activator GCN4 on chromatin and naked genomic DNA. GCN4 displays widespread and stable off-target binding on bare DNA because of entrapment by degenerate sites and interactions with nonspecific DNA of increasing length, indicative of one-dimensional (1D) diffusion. Nucleosome organization on the minichromosome reduces promiscuous GCN4 residence times by obstructing TF association and restricting 1D target search within nucleosome-free regions. Furthermore, the intrinsically disordered GCN4 activation domain independently enhances targeting efficiency and specificity by accelerating association–dissociation kinetics in vitro and in living cells. Altogether, both nucleosome organization and activation domains independently suppress promiscuous GCN4 binding, which, if unchecked, may cause aberrant cryptic transcription known to occur upon chromatin disruptions.

## Abstract (web summary)

Wei et al. measure single-molecule kinetics of transcription factor GCN4 on purified yeast minichromosomes to reveal that both nucleosomes and transcription factor intrinsically disordered regions facilitate GCN4 target search on native chromatin by shortening its promiscuous binding.

## Main

Gene expression under the control of sequence-specific transcription factors (TFs) provides the cornerstone of transcriptional programs directing general and cell-type-specific functions. Over 100 TFs in budding yeast and more than 1,000 mammalian TFs harbor DNA-binding domains (DBDs) that enable recognition of short *cis*-regulatory DNA elements throughout the genome. In the search for functional targets, TFs must navigate a vast excess of genomic DNA. For example, GCN4, the bZIP activator regulating budding yeast genes for amino acid biosynthesis^1^ and the yeast functional homolog of human AP1, binds to 546 targets comprising <0.1% of the 12.1-Mb yeast genome^2^.

How TFs find their cognate sites selectively and efficiently during exploration of the nucleoplasm and chromatin is critical for understanding the earliest stages of transcriptional control^3^. For prokaryotic TFs, efficient target search is theoretically achieved by the ‘facilitated diffusion’ model, which combines three-dimensional (3D) diffusion in solution and one-dimensional (1D) diffusion on bare DNA^4^. This has been experimentally validated for *Escherichia* *coli* LacI^5,6^ and further extended to human p53 and *Drosophila* GAGA factor^7,8^. However, eukaryotic genomes are organized into assemblies of nucleosomes, which have a general inhibitory effect on TF binding^9,10^. ATP-dependent chromatin remodelers have a central role in the establishment and maintenance of nucleosome-free or nucleosome-depleted regions (collectively, NFRs) on promoter and enhancer DNAs^11–16^ bracketed by nucleosome roadblocks to 1D TF diffusion^8^. The impact of chromatin on TF targeting has been revealed by steady-state genomic studies^9,10^ and TF dynamics have also been quantified in living cell nuclei by single-particle tracking^17^; however, the kinetics of target search and TF residency on native chromatin domains comprising entire genes and regulatory elements have not been explored.

In addition, eukaryotic TFs recognize short, degenerate sequences with lower information content than prokaryotic regulators (11 bits for GCN4 versus 31 bits for LacI)^18–21^. TF binding to such core cognate motifs was recently shown to be modulated by flanking sequences^22–25^. The stochastic occurrence of imperfect matches to TF cognate motif could trap eukaryotic TFs away from their intended targets and, thus, interfere with the search process. Furthermore, genome-wide TF binding to sites lacking clear cognate motifs can also be directed by their activation domains (ADs) or intrinsically disordered regions (IDRs)^26,27^. These observations underscore the importance of studying full-length TFs and gene-size (kb-scale) chromatin substrates when measuring the kinetics of target search.

Here, we apply single-molecule total internal reflection fluorescence (smTIRF) imaging to elucidate kinetic mechanisms that regulate GCN4 targeting on the complete *HIS3* gene within a 3.1-kb purified native yeast minichromosome (MC)^28–30^. We provide evidence for substantial off-target GCN4 binding and length-dependent scanning on histone-free DNA and quantify the chromatin-based suppression of promiscuous GCN4 binding to enhance promoter-specific targeting. We also demonstrate that the GCN4 AD, separate from its traditional role in recruiting transcription preinitiation proteins, can accelerate TF association and dissociation to boost targeting efficiency and specificity, which we further validate in living yeast.

## smTIRF microscopy of purified MCs

The budding yeast MC^28–30^ is a kb-scale, circular, endogenously chromatinized plasmid harboring an origin of replication, a selectable marker, tandem bacterial operator sites for affinity purification and an insertion of any yeast gene of interest (for example, the GCN4 target *HIS3*), which recapitulates the inducible expression of the endogenous gene^1,29,31,32^. In the yeast genome, GCN4 binds specifically to the upstream activating sequence (UAS) of *HIS3*, as shown by ChIP-seq^33^; however, in vitro G-SELEX analysis revealed >1,000 off-target GCN4-binding sites on yeast genomic DNA with low in vivo occupancy^1^.

**Fig. 1:**
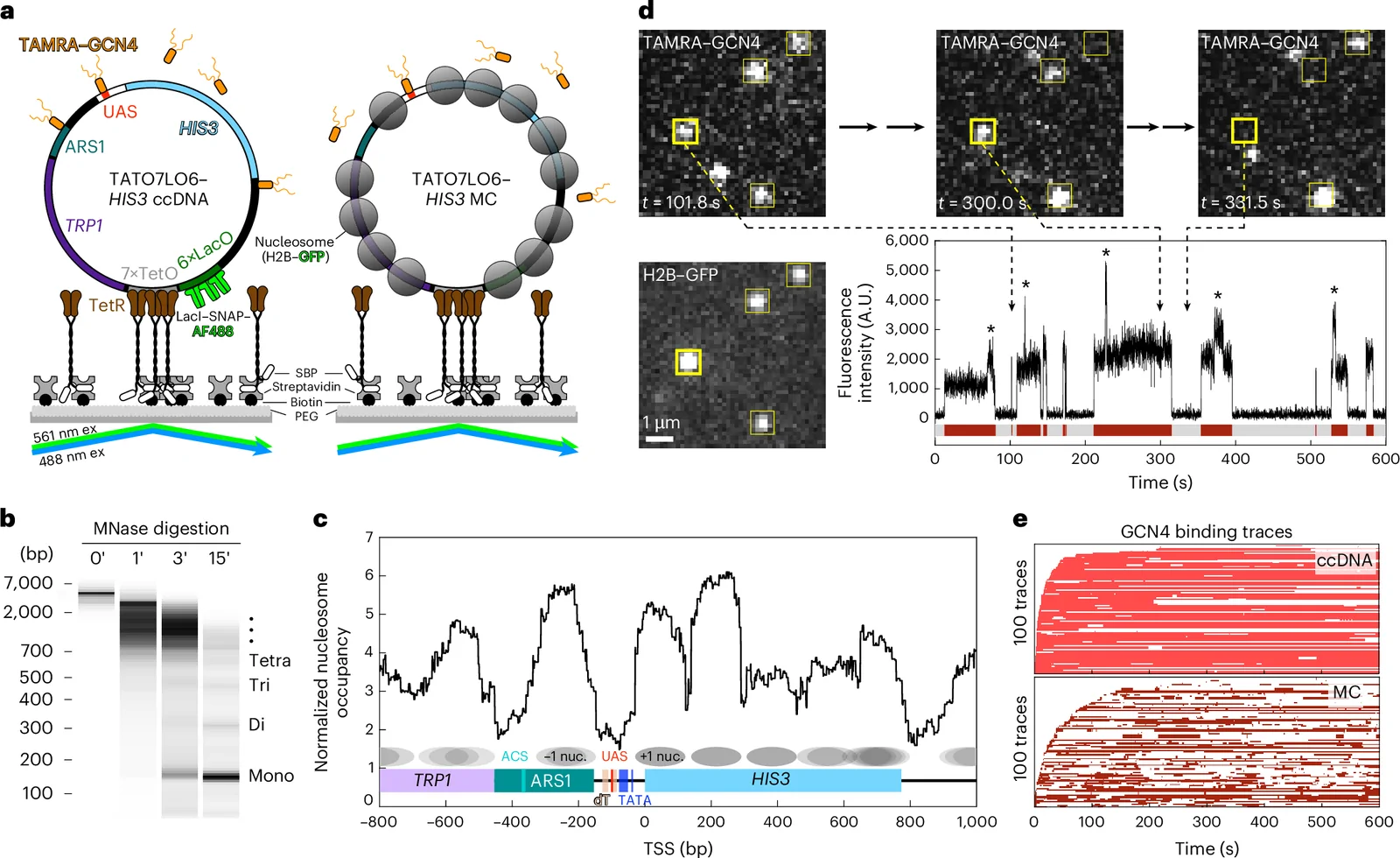
GCN4 single-molecule binding kinetics on purified, immobilized MCs. **a**, Schematics of purified 3.1-kb ccDNA and MC immobilized on PEGylated TIRF slides. Right, spheres represent H2B–GFP-labeled nucleosomes. SBP, streptavidin-binding peptide. **b**, MNase digestion patterns of purified MC, analyzed on an Agilent Bioanalyzer. All MC samples used in this work show similar band patterns. **c**, Normalized nucleosome occupancy (filtered for 125–175-bp reads) across *HIS3* from purified MC determined by MNase-seq. Gray ovals indicate deduced nucleosome positions. The bar diagram shows *cis* elements: ACS, ARS consensus sequence (ORC binding); ARS1, autonomously replicating sequence 1; dT, poly(dT) sequence; UAS, GCN4-binding motif. **d**, Representative TIRF video frames and TAMRA–GCN4 fluorescence trace over time. In the H2B–GFP panel, MC spots are boxed; the example GCN4 trace is generated from the bolded box. The TAMRA–GCN4 panels show the same field of view at indicated times (arrows). Asterisks indicate additional GCN4-binding events. The bar diagram shows GCN4-bound (dark red) and unbound (gray) states. **e**, Rastergrams of 100 randomly selected GCN4 traces on ccDNA or MC templates arranged in order of first GCN4-binding event after flow-in at time 0. Source data

**Extended Data Fig. 1:**
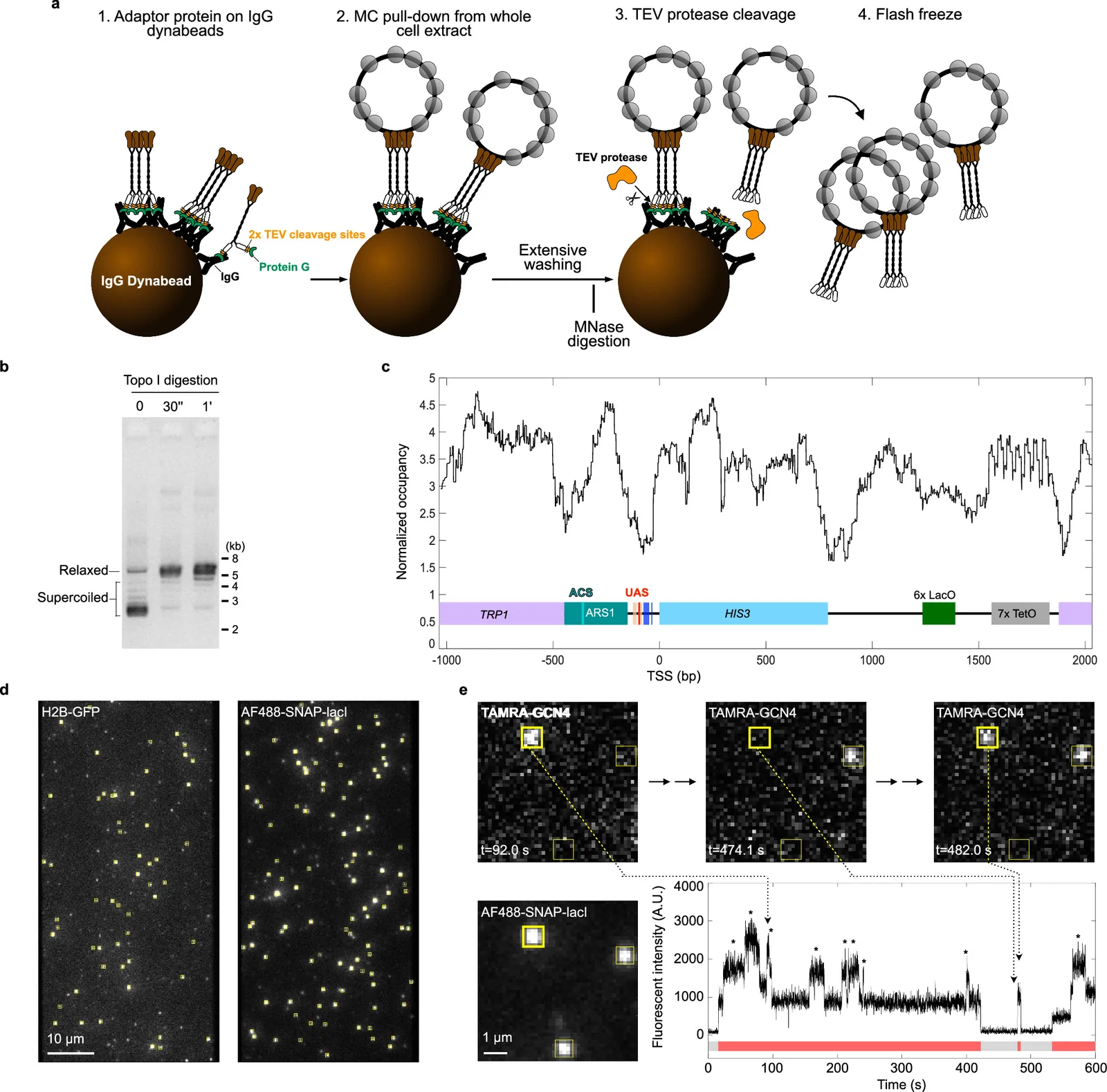
Purification and characterization of MC, and TIRF imaging of GCN4 binding to ccDNA. **a**, Schematics of MC purification. **b**, Topo I digestion pattern of purified TATO21 (TRP1-ARS1-TetOx21) MC backbone. 2 independent experiments yield the same results. **c**, Normalized protein occupancy (MNase-seq) on TATO7LO6*HIS3* MC without filtering read length. **d**, Representative field of view of immobilized MC (H2B-GFP) and ccDNA (AF488-SNAP-lacI). All MC and ccDNA samples in this work show similar fields of view. **e**, Representative video frames and GCN4 fluorescence trace on immobilized ccDNA as in Fig. 1c. Source data

To test the hypothesis that chromatin suppresses off-target GCN4 binding, we introduced a 3.1-kb MC carrying the *HIS3* gene (TATO7LO6–HIS3: TRP1–ARS1–TetO7–LacO6–HIS3) (Fig. 1a) into a *HTB2*–*GFP* knock-in yeast strain to enable detection of the purified MC (Extended Data Fig. 1a). The *HIS3* MC is organized in an array of nucleosomes (Fig. 1b), displaying a well-positioned +1 nucleosome with an upstream border at −29 bp relative to the transcription start site (TSS), a −1 fuzzy nucleosome with a downstream border at ~−150 bp and an NFR of ~120 bp spanning the *HIS3* promoter harboring poly(dT) sequences, TATA boxes and a single UAS (Fig. 1c). The chromatin organization of the *HIS3* MC, typical of yeast promoters, is consistent with previous studies of genomic and minichromosomal *HIS3* (refs. ^29,34,35^). In addition to the *HIS3* promoter NFR, we observed NFRs at ARS1, the *HIS3* 3′ end (Fig. 1c) and the *TRP1* promoter, as well as TetR footprints over tandem operators because of affinity purification (Extended Data Fig. 1c).

**Extended Data Fig. 2:**
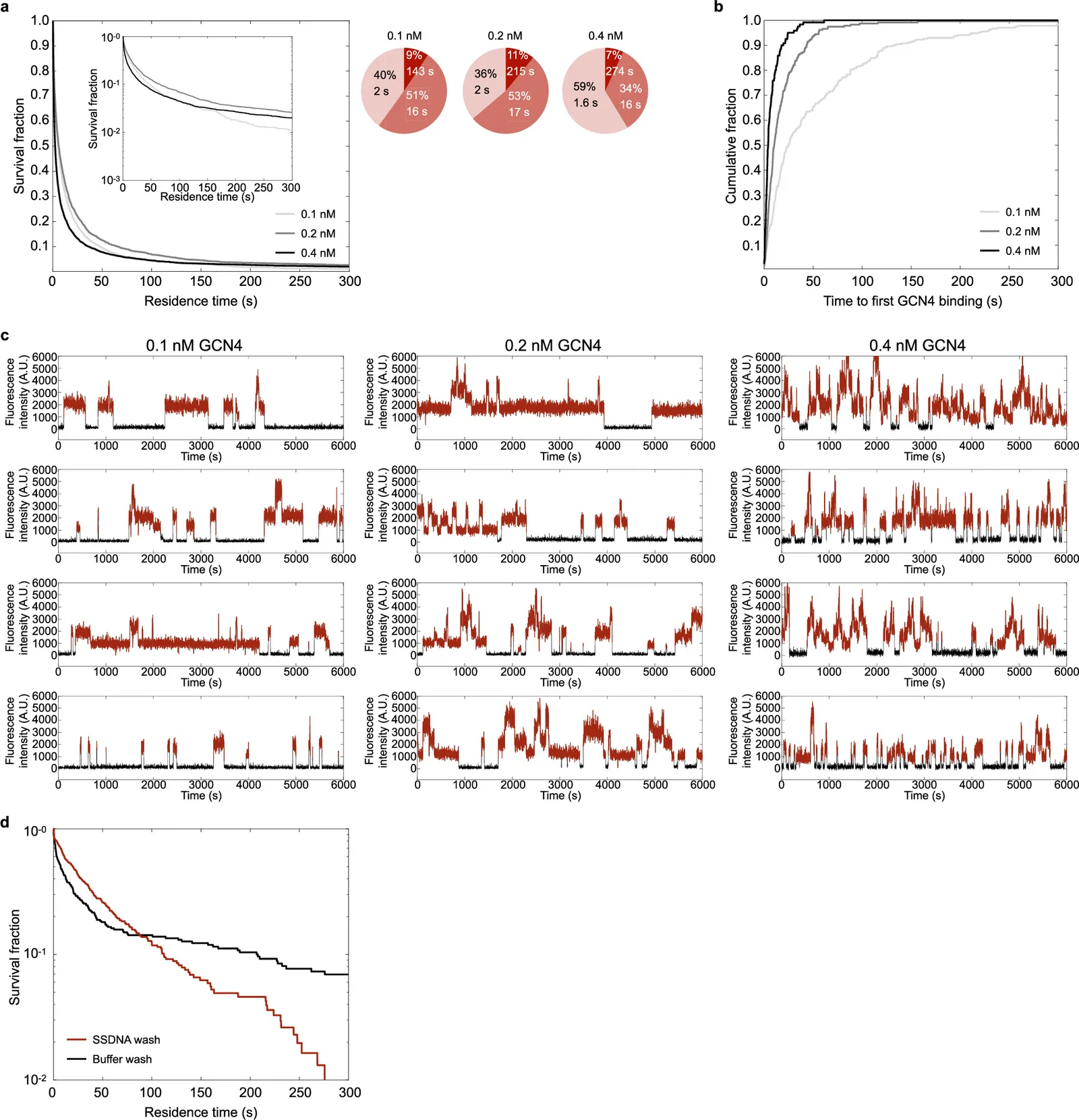
Titration of GCN4 on WT MC. **a**, 1-CDF for GCN4 τ at different GCN4 concentrations, with exponential fitting shown by pie charts. Inset shows the same plots with semi-log scale. GCN4 concentration does not interfere with τ distribution in general, with small divergence when τ>150 s. **b**, CDF for GCN4 association time (time interval between GCN4 flow-in and first binding event) at different concentrations. Association time shows strong anti-correlation to GCN4 concentration. **c**, Example fluorescence traces at different GCN4 concentrations. Frequency of multi-GCN4 binding increases with GCN4 concentration. **d**, 1-CDF for GCN4 in vitro τ when washed with buffer or 0.1 mg/mL salmon sperm DNA (SSDNA) as competitor during TIRF imaging. Source data

The MC or its closed circular DNA (ccDNA) appears as bright fluorescent spots upon immobilization for TIRF imaging (Fig. 1d and Extended Data Fig. 1d–e). We measured TAMRA–GCN4 binding under physiological salt conditions to individually resolved MCs or ccDNAs by fluorescence colocalization, which showed single (or multiple) GCN4 molecules binding to the 3.1-kb substrate as indicated by the abrupt increase in TAMRA fluorescence (Fig. 1d and Extended Data Fig. 1e). To minimize multiple GCN4-binding events (Fig. 1d and Extended Data Figs. 1e and 2a,c), we performed measurements at low GCN4 concentration (0.1 nM) and call discrete TAMRA–GCN4 signals above background as the bound state. Inspection of rastergram profiles for GCN4 association and dissociation on individual *HIS3* MC or ccDNA molecules (Fig. 1e) revealed qualitatively that chromatin organization regulates GCN4 target search by both lowering the GCN4 association rate (*k*_on_) and raising the dissociation rate (*k*_off_).

## Off-target GCN4 binding to naked DNA

**Fig. 2:**
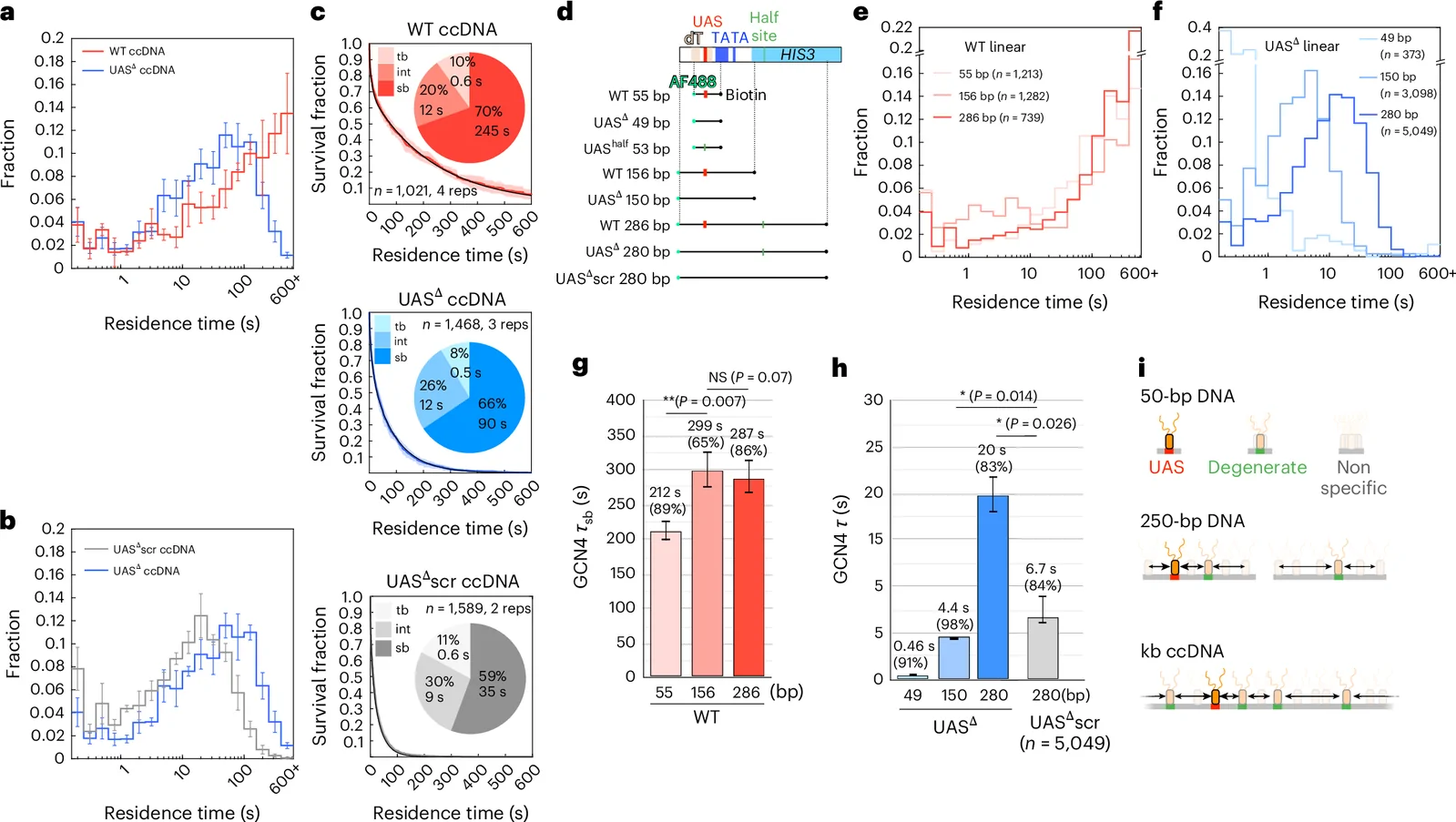
GCN4 displays stable ectopic entrapment by degenerate motifs and length-correlated nonspecific binding on naked DNA. **a**,**b**, GCN4 residence time (*τ*) histogram (average of replicates, *x* axis log-binned) for WT and UAS^∆^ ccDNA (**a**) and for UAS^∆^scr (scrambled degenerate motifs) ccDNA (**b**). Error bars indicate the s.d. among all replicates (**c**). The last bin includes binding events > 600 s. **c**, Curves of 1 − CDF for data in **a**,**b**. Shadows indicate the s.d. among replicates and thin black curves represent exponential fits. Pie charts represent the three-component exponential fit for GCN4-binding fractions (%) and *τ* (s) on WT, UAS^∆^ and UAS^∆^scr ccDNA. The numbers of GCN4-binding events (*n*) and biological replicates are labeled. **d**, Linear DNA fragments for TIRF microscopy with UAS and half-site marked. **e**,**f**, GCN4 *τ* histogram for WT (**e**) and UAS^∆^ (**f**) linear DNA fragments. **g**, GCN4 *τ*_sb_ (F_sb_ in brackets) on WT linear DNA fragments (**e**). Column values indicate the fitting result of original dataset and error bars indicate the 95% confidence intervals (CIs) determined by bootstrapping. *P* values were calculated using a double-tailed *t*-test (Methods). NS, not significant. **h**, GCN4 major *τ* (dominant fraction in brackets) on UAS^∆^ (**f**) and UAS^∆^scr linear DNA fragments. Column values, error bars and *P* values are as in **g**. **i**, Model of GCN4 1D diffusion on bare DNA. When a specific target (UAS) is present, GCN4 is stably captured and *τ* is dominated by target-specific binding. On nonspecific DNA of greater length, there are more degenerate motifs and nonspecific DNA along which GCN4 diffuses in 1D, prolonging its overall off-target *τ*. Source data

**Extended Data Fig. 3:**
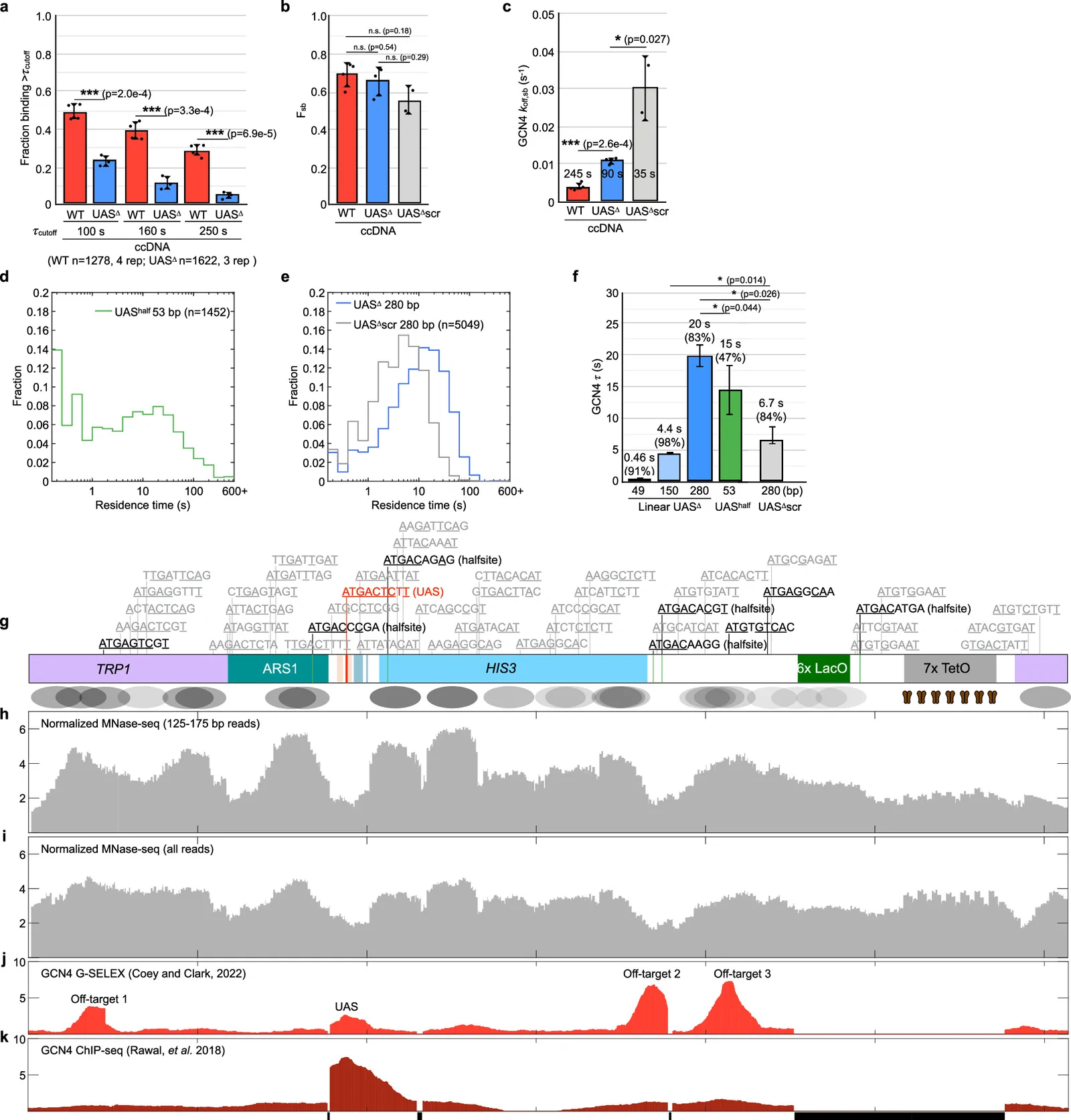
GCN4 binding to degenerate motifs on naked ccDNA and DNA fragments. **a**, Fraction of binding events > indicated τ_cutoff_ on ccDNA (binding event n and replicate number labeled). **b-c**, GCN4 F_sb_ (**b**) and *k*_off,sb_ (**c**) for different ccDNAs. τ_sb_ = 1/*k*_off,sb_ labeled for each column in (**c**). (**b**) and (**c**) are derived from the same datasets as Fig. 2c. For (**a**)–(**c**), columns indicate averages of biological replicates, error bars indicate S.D. between replicates, and p-values are calculated by double-tailed t-test. **d-e**, GCN4 τ histogram for 53-bp UAS^half^ DNA (**d**) and 280-bp UAS^∆^scr DNA (**e**). **f**, same as Fig. 2h with UAS^half^ included. **g**, Half-sites and other degenerate motifs that have at least 6 matches (underscored) to ‘ATGACTCAT’ are marked grey or black above the bar diagram. We note that, unlike other GCN4 target genes such as *ARG1*, the *HIS3* UAS is the closest to, but not a perfect match to, the highest-affinity site. For UAS^∆^scr templates, sequences in black are scrambled. Ovals below indicate deduced nucleosome and TetR positions. **h-i**, Filtered (**h**, as Fig. 1c) and unfiltered (**i**, as Extended Data Fig. 1e) MNase-seq coverage on MC. **j**, G-SELEX data from Coey and Clark^2^. **k**, GCN4 ChIP-seq data extracted from Rawal, *et al*.^33^ applied to MC sequence. Black blocks at the bottom indicate artificial fusion or insertion on MC sequence. Source data

We first examined GCN4 kinetics on the 3.1-kb ccDNA template (TATO7LO6–HIS3) devoid of histones. To quantify dissociation rates (*k*_off_ = 1/*τ*), we plotted the residence time (*τ*) histogram for thousands of binding events and applied exponential fitting to extract *k*_off_. The *τ* histogram for the wild-type (WT) *HIS3* ccDNA reveals a large GCN4-binding fraction with *τ* > 100 s (49%, *n* = 1,021; Fig. 2a and Extended Data Fig. 3a). Deletion of the core sequence ‘TGACTC’ from the UAS (UAS^∆^ ccDNA) shifted the GCN4 *τ* peak to 50–100 s, which was still surprisingly stable, and correspondingly decreased the *τ* > 100 s fraction (23%, *n* = 1,468; Fig. 2a and Extended Data Fig. 3a). Because a previous study^1^ identified off-target GCN4-binding sites located beyond the UAS on the naked *HIS3* MC DNA sequence, we scrambled the underlying degenerate motifs (sequences mismatched to the cognate motifs) plus additional half-sites (UAS^∆^scr ccDNA; Extended Data Fig. 3g,j), which resulted in a further histogram peak shift to ~20 s (*n* = 1,589; Fig. 2b).

Exponential fitting of GCN4 residence time (Fig. 2c) discerned three binding populations: stable (sb), intermediate (int) and transient (tb), reflecting different levels of binding stability. For WT ccDNA, the dominant stable fraction (F_sb_=70%) displayed *τ*_sb_ of 245 s, while UAS^∆^ and UAS^∆^scr ccDNAs gave lower *τ*_sb_ of 90 s (F_sb_ = 66%), and 35 s (F_sb_ = 59%), respectively (*P* ≤ 0.027; Fig. 2c and Extended Data Fig. 3b,c). Note that F_sb_ and *τ*_sb_ for WT ccDNA may have been underestimated because of partial exclusion of right-censored binding events (Methods). These changes in *τ*_sb_ represent progressively weaker stabilities of GCN4 on the UAS, degenerate sites and other nonspecific sequences. Thus, degenerate motifs and the entire spectrum of nonspecific DNA sequences on the 3.1-kb *HIS3* ccDNA contribute to off-target GCN4 residency.

**Extended Data Fig. 4:**
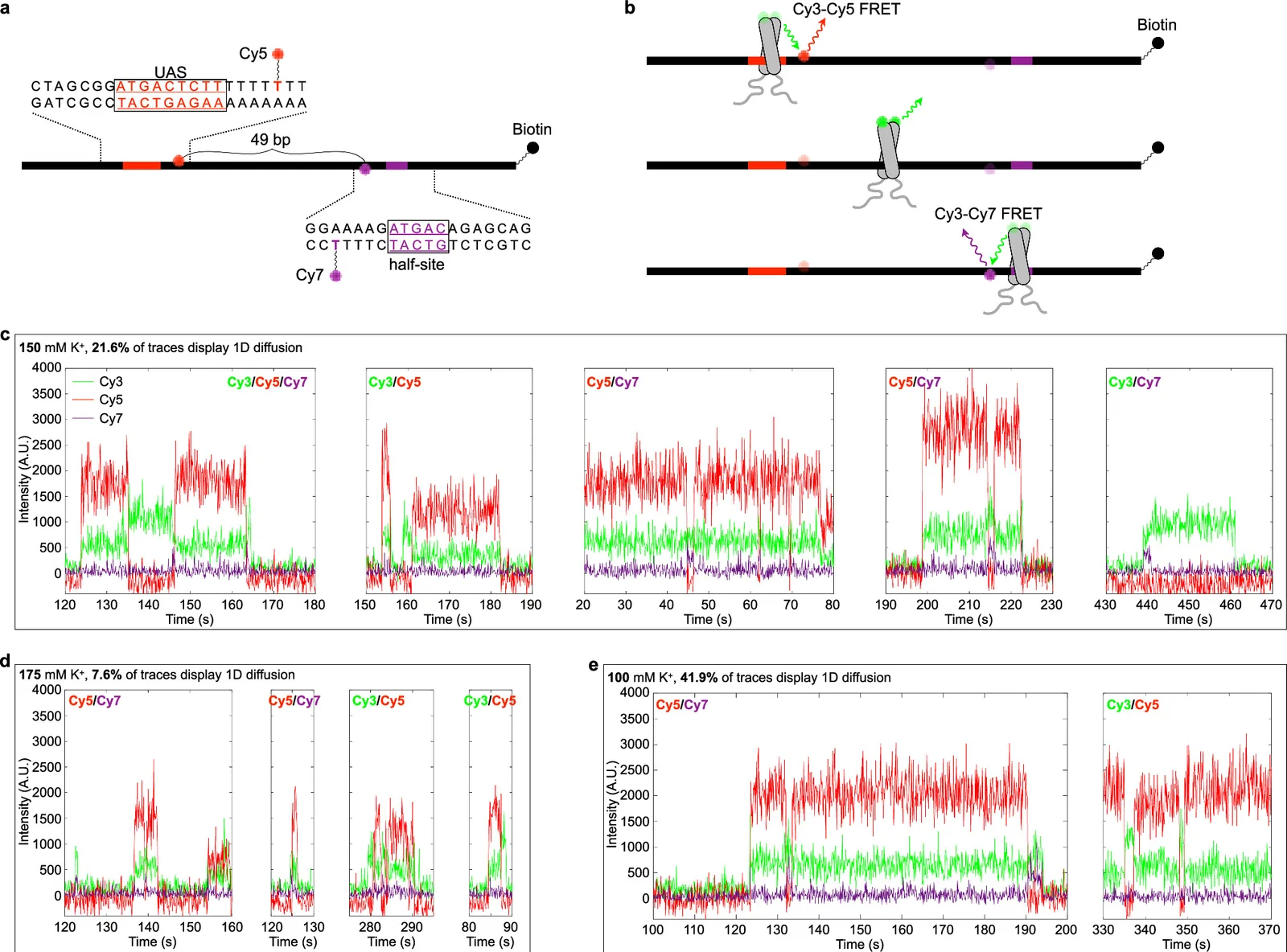
GCN4 1D diffusion on linear DNA. **a**, Schematics of dual-labeled 118-bp DNA fragments used for 3-color FRET. **b**, Illustration of 3-color FRET. When GCN4-Cy3 binds at UAS, Cy3-Cy5 FRET will be observed; at half-site, Cy3-Cy7 FRET; at other off-target sequences, Cy3-only signals. **c**, Example traces showing GCN4 1D diffusion at 150 mM KOAc (n = 1727 traces). Fraction of traces displaying 1D diffusion labeled at the top of the box. For each example trace, state switching is labeled at the top, where Cy5 indicates binding at UAS, Cy7 indicates binding at half-site, and Cy3 indicates binding at other off-target sequences. **d-e**, 1D diffusion FRET traces under 175 mM (**d**; n = 776 traces) and 100 mM (**e**; n = 739 traces) KOAc, respectively. Format same as (**c**).

Given the stable GCN4 off-target binding on 3.1-kb ccDNA, we considered whether DNA length also has an impact on GCN4 *τ*. On short WT *HIS3* promoter DNA fragments of 55 bp, 156 bp and 286 bp (Fig. 2d), GCN4 displayed *τ*_sb_ of 212 s, 299 s and 287 s (F_sb_ = 89%, 65% and 86%), respectively, comparable to *τ*_sb_ = 245 s (F_sb_ = 70%) for the 3.1-kb WT ccDNA (Fig. 2e,g). On UAS-deleted DNA fragments (UAS^∆^) of 49 bp, 150 bp and 280 bp (Fig. 2d), the dominant GCN4-binding population displayed increasing *τ* values of 0.46 s, 4.4 s and 20 s (F = 91%, 98% and 83%), respectively, in positive correlation with increasing DNA length (Fig. 2f,h). To eliminate the effect of degenerate motif entrapment on the 280-bp UAS^∆^ DNA harboring one half-site (Fig. 2d), we scrambled the half-site (280-bp UAS^∆^scr), leading to a *τ* decrease to 6.7 s (Fig. 2h and Extended Data Fig. 3e,f), still correlated with DNA length. These observations are consistent with previous findings for murine Sox2 (ref. ^36^) and support GCN4 1D diffusion on naked DNA. This was further confirmed by three-color fluorescence resonance energy transfer (FRET) analysis^6,8^ of GCN4 binding to linear promoter DNA harboring the UAS and a half-site (Extended Data Fig. 4). We note that GCN4 1D diffusion displayed salt sensitivity (Extended Data Fig. 4c–e), similar to GAGA factor^8^, suggesting that 1D hopping has a role in GCN4 1D diffusion. Altogether, both ectopic entrapment at degenerate motifs and promiscuous interactions with nonspecific sequences during GCN4 1D diffusion are responsible for stable off-target residence on naked DNA (Fig. 2i).

## Chromatin suppresses stable off-target GCN4 binding by blocking DNA accessibility

**Fig. 3:**
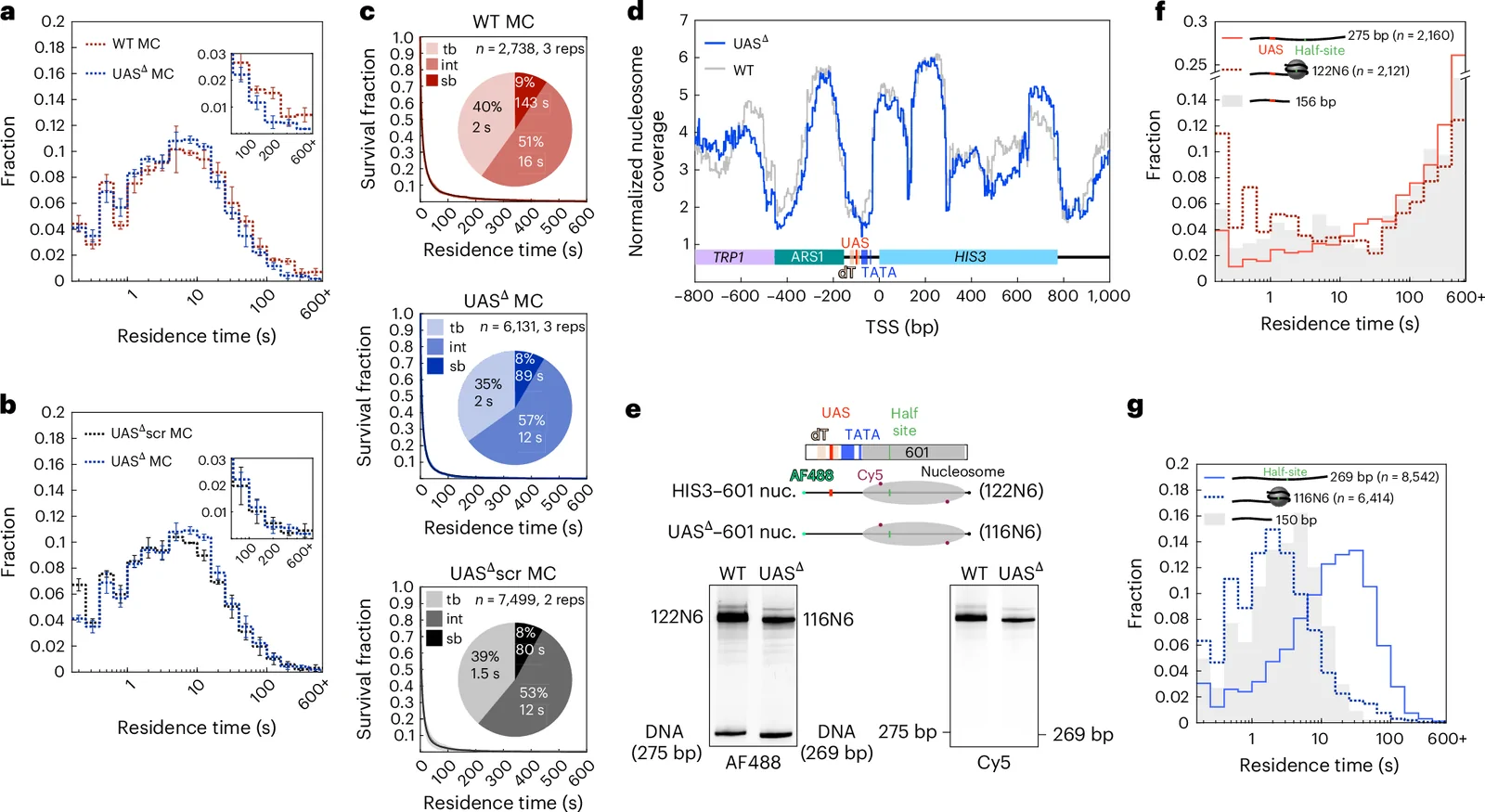
Chromatin suppresses stable GCN4 binding by confining DNA exposure to NFRs. **a**,**b**, GCN4 residence time (*τ*) histogram (average of replicates) for WT and UAS^∆^ MC (**a**) and for UAS^∆^scr MC (**b**). Error bars indicate the s.d. among all replicates (**c**). Insets, zoomed-in view of *τ* > 60 s. **c**, Curves of 1 − CDF and pie charts representing three-component exponential fit for WT, UAS^∆^ and UAS^∆^scr MC (formats as in Fig. 2c). The numbers of binding events (*n*) and biological replicates are labeled. **d**, Normalized nucleosome occupancy on UAS^∆^ MC compared to WT by MNase-seq. **e**, Top, reconstituted long-linker mononucleosomes for TIRF microscopy. Bottom, AF488–DNA and Cy5–H2B scans of nucleosomes on native PAGE gel (three independent electrophoreses show similar band patterns). Solid lines in the Cy5 panel mark the positions of 275-bp and 269-bp DNA bands. **f**,**g**, GCN4 *τ* histogram for WT (**f**) and UAS^∆^ (**g**) long-linker nucleosomes (with half-site). Gray shading represents *τ* histograms for 156-bp WT (**f**) or 150-bp UAS^∆^ (**g**) linear DNA. Source data

**Extended Data Fig. 5:**
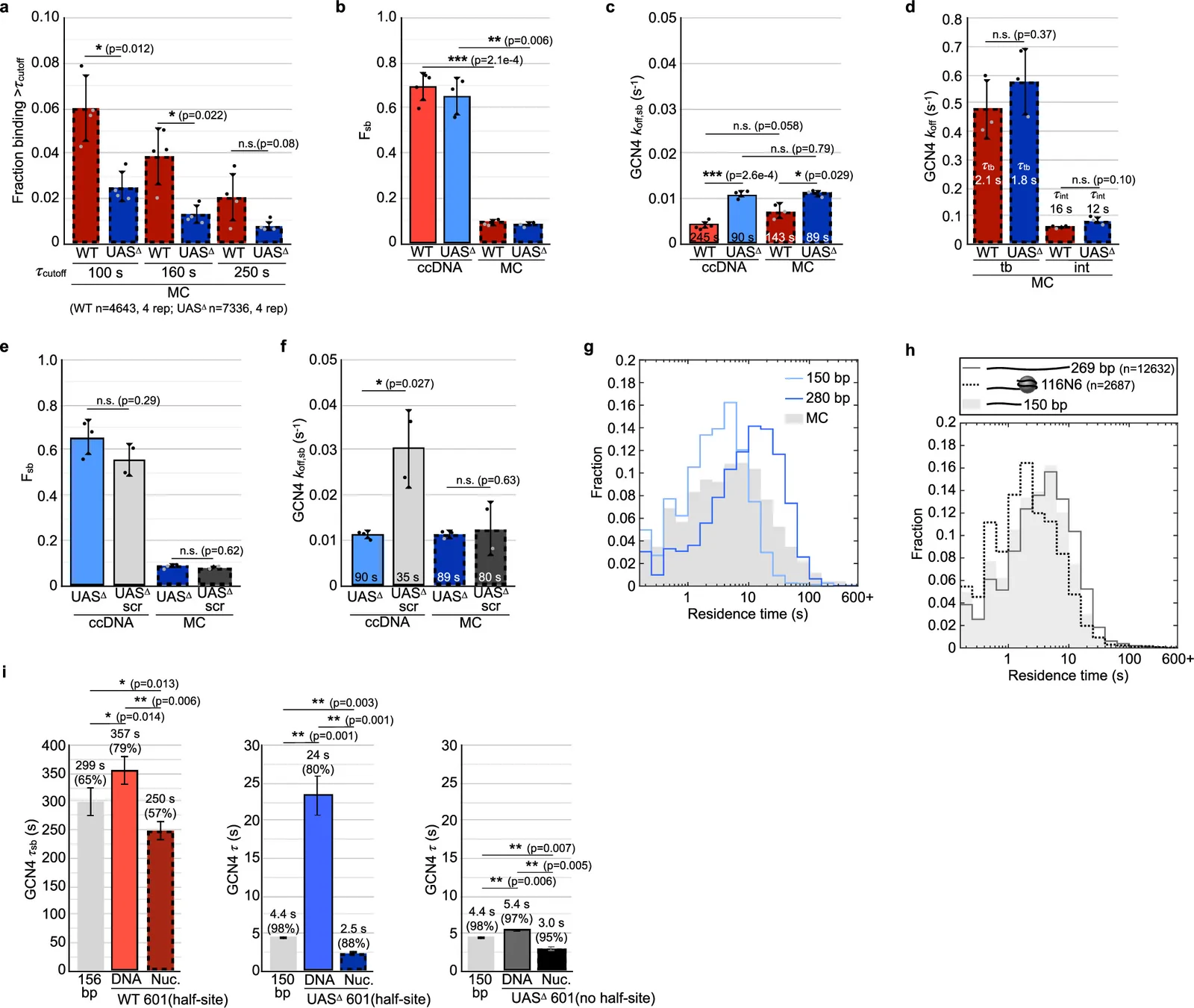
Comparative GCN4 residence time (τ) on naked DNA and chromatin. **a**, Fraction of binding events > indicated τ_cutoff_ on MC. **b, e**, Comparison of GCN4 F_sb_ between WT and UAS^∆^ (**b**) or UAS^∆^ and UAS^∆^scr (**e**) ccDNA and MC. **c, f**, Comparison of GCN4 *k*_off,sb_ between WT and UAS^∆^ (**c**) or UAS^∆^ and UAS^∆^scr (**f**) ccDNA and MC. **d**, Comparison of GCN4 *k*_off,tb_ and *k*_off,int_ between WT and UAS^∆^ MC, with τ = 1/*k*_off_ as indicated. For (**a**)-(**f**), columns indicate average values of biological replicates and error bars indicate S.D. between replicates. P-values calculated by double-tailed t-test among replicates. **g**, Comparison of GCN4 τ distribution between UAS^∆^ linear DNA and MC. **h**, GCN4 τ histogram for UAS^∆^ 116N6 nucleosomes without half-site (grey shade represents τ histograms for 150-bp UAS∆ linear DNA). **i**, GCN4 τ_sb_ (F_sb_ in brackets) or major τ (fraction in brackets) for long linker nucleosomes in comparison with linear DNA. Columns indicate fitting values of original datasets and error bars indicate 95% C.I. determined by bootstrapping. Source data

The dissociation kinetics of GCN4 on the MC differ greatly in comparison to ccDNA. The GCN4 *τ* histogram showed a peak shift from >100 s for WT ccDNA (Fig. 2a) to ~10 s for the WT MC (*n* = 2,738; Fig. 3a). Exponential fitting also revealed a major decrease in F_sb_ (70% to 9%, *P* < 0.001) and reduction in *τ*_sb_ (245 s to 143 s, *P* = 0.058; Fig. 3c and Extended Data Fig. 5a–c). Correspondingly, intermediate and transient GCN4-binding populations expanded on WT MC (F_int_ = 51%, *τ*_int_ = 16 s; F_tb_ = 40%, *τ*_tb_ = 2 s; Fig. 3c). The results indicate that nucleosome arrangement on the MC has a major inhibitory effect on GCN4 stable binding.

Deletion of the UAS (UAS^∆^ MC, *n* = 6,131) did not alter nucleosome arrangement (Fig. 3d) but reduced *τ*_sb_ (from 143 s to 89 s, *P* = 0.029), while intermediate and transient fractions and *τ* values remained the same as the WT MC (Fig. 3a,c and Extended Data Fig. 5b–d). Hence, off-target GCN4 binding on the UAS^∆^ MC is dominated by intermediate (57%, 12 s) and transient (35%, 2 s) populations, in contrast to UAS^∆^ ccDNA, where off-target binding is dominated by the stable population (66%, 90 s; Fig. 2c). Although degenerate motif interactions are a major cause of stable off-target binding on ccDNA, scrambling degenerate motifs on the MC (UAS^∆^scr MC, *n* = 7,499) did not change the overall *τ* distribution (Fig. 3b,c and Extended Data Fig. 5e,f), indicating that nucleosome organization sufficiently suppresses off-target GCN4 entrapment.

Notably, the GCN4 *τ* histogram profile for the *HIS3* MC was intermediate to the *τ* profiles for 150-bp and 280-bp linear UAS^∆^ naked DNA (Extended Data Fig. 5g), consistent with average NFR lengths on the MC (Fig. 1c and Extended Data Fig. 1c). This suggests that nucleosomes limit GCN4 off-target search time by confining DNA exposure to NFRs. We further analyzed GCN4 dissociation kinetics using a reconstituted ‘long-linker’ nucleosome containing a positioned Widom 601 core particle (nucleosomal H2B is Cy5-labeled), with substitution of a half-site between SHL-2 and SHL-2.5 to mimic the *HIS3* + 1 nucleosome and a 122-bp extension of linker DNA containing the *HIS3* promoter with UAS (Fig. 3e). For this WT long-linker nucleosome (122N6), the GCN4 *τ* histogram showed a decrease in the predominant >100-s fraction compared to unreconstituted bare DNA (Fig. 3f), with a corresponding decrease in F_sb_ and *τ*_sb_ (Extended Data Fig. 5i).

For the UAS^∆^ nucleosome (116N6, with half-site), there was also a substantial left-shift in the *τ* histogram compared to bare DNA (Fig. 3g), with a corresponding reduction in *τ* from 24 s to 2.5 s—shorter *τ* than for naked 150-bp UAS^∆^ DNA (*P* = 0.003; Extended Data Fig. 5i). The same analysis on UAS^∆^ 116N6 without the half-site also revealed a reduction in GCN4 *τ* from 5.4 s on bare DNA to 3.0 s (*P* = 0.005; Extended Data Fig. 5i). Hence, nucleosomes modulate GCN4 residence time by limiting bare DNA length and restricting 1D diffusion.

## Promoter NFR length determines the association rate of stable GCN4 binding

**Fig. 4:**
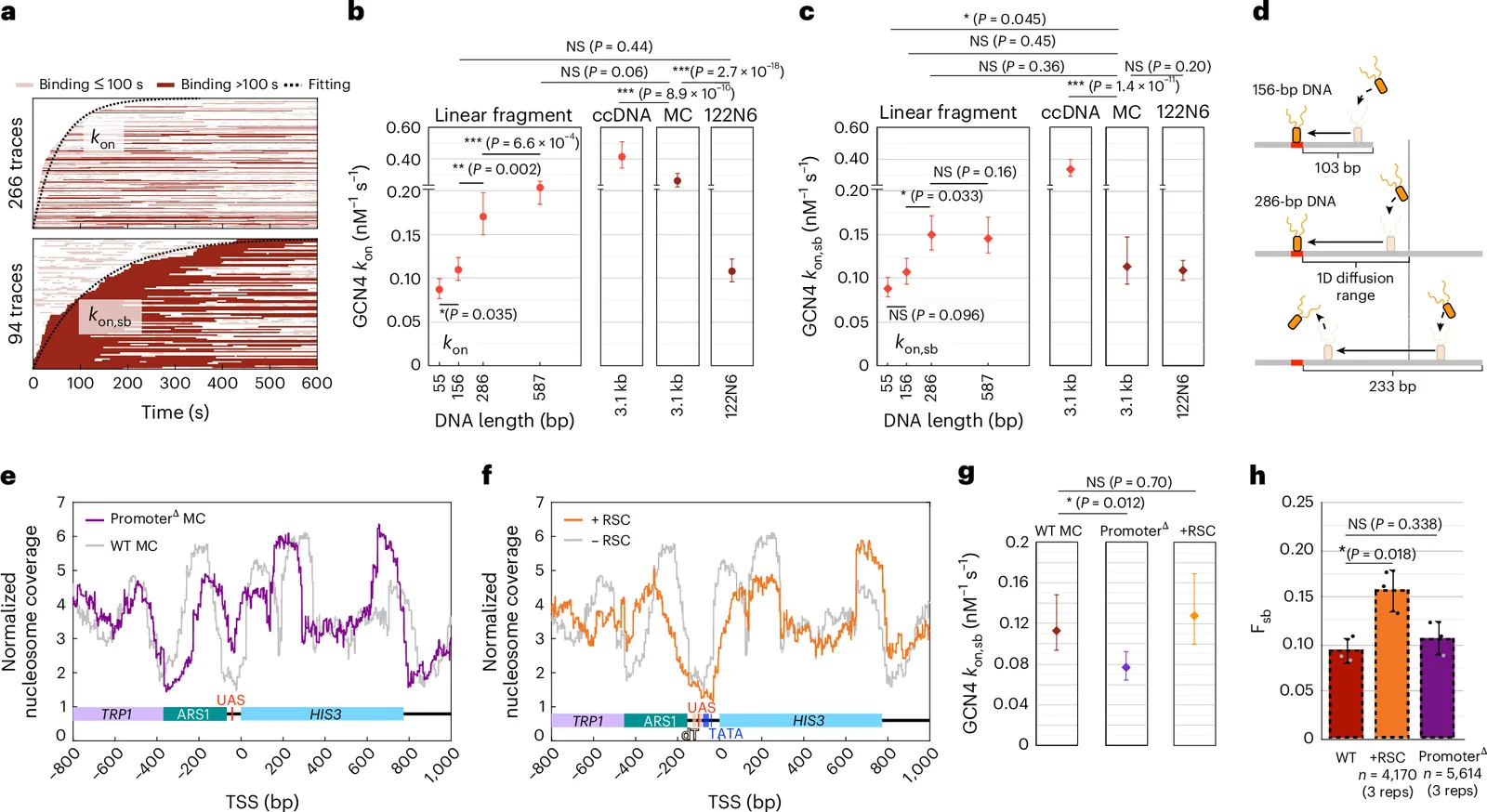
Promoter NFR length modulates GCN4 association rate and fraction of stable binding. **a**, Rastergrams of GCN4 traces sorted by first binding event (top) or first stable binding event (>100 s; bottom) to WT MC. Dotted lines represent exponential fitting for GCN4 *k*_on_ (top) and *k*_on,sb_ (bottom). **b**,**c**, GCN4 *k*_on_ (**b**) and *k*_on,sb_ (**c**; for binding events > 100 s) for WT templates as indicated (same datasets as Figs. 2 and 3). For linear DNA templates, the *x* axis is in linear scale with DNA length labeled. Dot and diamond values are fitting results of the original datasets and error bars indicate the 95% CIs determined by bootstrapping. *P* values were calculated using a double-tailed Wilcoxon rank-sum test. The numbers of analyzed traces (*n*) were as follows: 55 bp, 232; 156 bp, 279; 286 bp, 203; 587 bp, 294; ccDNA, 396; MC, 266; 122N6, 266. **d**, Graphics show how *k*_on,sb_ is limited by 1D diffusion. When the distance from GCN4 landing position to the UAS is too long, GCN4 is more likely to dissociate before reaching UAS (red). **e**,**f**, Normalized nucleosome occupancy on promoter^∆^ (**e**) and RSC-treated (**f**) MCs. In **e**, promoter^∆^ and WT MCs are aligned at the shared UAS position. **g**, GCN4 *k*_on,sb_ for WT, promoter^∆^ and RSC-treated MCs (formats for statistics as in **c**). The numbers of analyzed traces (*n*) were as follows: promoter^∆^ MC, 245; RSC-treated MC, 169 (same datasets as **h**). **h**, F_sb_ for WT, promoter^∆^ and RSC-treated MCs. Column values indicate averages of replicates and error bars indicate the s.d. among biological replicates. *P* values were calculated using a double-tailed *t*-test. The numbers of binding events (*n*) and biological replicates are labeled. Source data

To complement the kinetics of GCN4 dissociation, we also determined the association rate by measuring the interval between GCN4 flow-in and the first binding event (Fig. 4a). The general association rate constant (*k*_on_) on naked *HIS3* DNA correlated well with template length (Fig. 4b); 55-bp DNA displayed the lowest *k*_on_ (0.087 nM^−1^ s^−1^), increasing progressively by ~5-fold (to 0.41 nM^−1^ s^−1^) for the 3.1-kb ccDNA. For the four NFRs on the *HIS3* MC with ~500 bp of total bare DNA, the general *k*_on_ (0.26 nM^−1^ s^−1^) was comparable to the value for a 587-bp linear DNA fragment (0.22 nM^−1^ s^−1^, *P* = 0.06), suggesting that the general *k*_on_ is mainly driven by NFRs. In addition, the *k*_on_ for a 122N6 long-linker nucleosome and 156-bp naked promoter DNA was almost equivalent (0.11 nM^−1^ s^−1^, *P* = 0.44), indicating that the general *k*_on_ of GCN4 is primarily determined by the total length of NFR DNA, with little contribution from the nucleosome core particle. In other words, GCN4 association predominantly occurs at NFRs.

For the stable binding GCN4 subpopulation representing UAS-specific association on the MC, we determined the stable association rate constant (*k*_on,sb_) for binding events *τ* > 100 s (Fig. 4a,c). Unlike general *k*_on_, the *k*_on,sb_ reached a plateau around 0.15 nM^−1^ s^−1^ as linear DNA length increased to 286 bp (Fig. 4c), suggesting that *k*_on,sb_ is limited by the distance range of 1D diffusion before entrapment or dissociation^37,38^ (Fig. 4d). Of interest, *k*_on,sb_ apparently exceeded this limit for ccDNA (‘Discussion’). Notably, the WT MC, the 122N6 long-linker nucleosome and the 156-bp naked promoter DNA all showed comparable *k*_on,sb_ values (0.11, 0.12 and 0.11 nM^−1^ s^−1^, respectively, *P* ≥ 0.20), indicating that NFR length at *HIS3* promoter determines the GCN4 association rate for UAS-specific binding.

**Extended Data Fig. 6:**
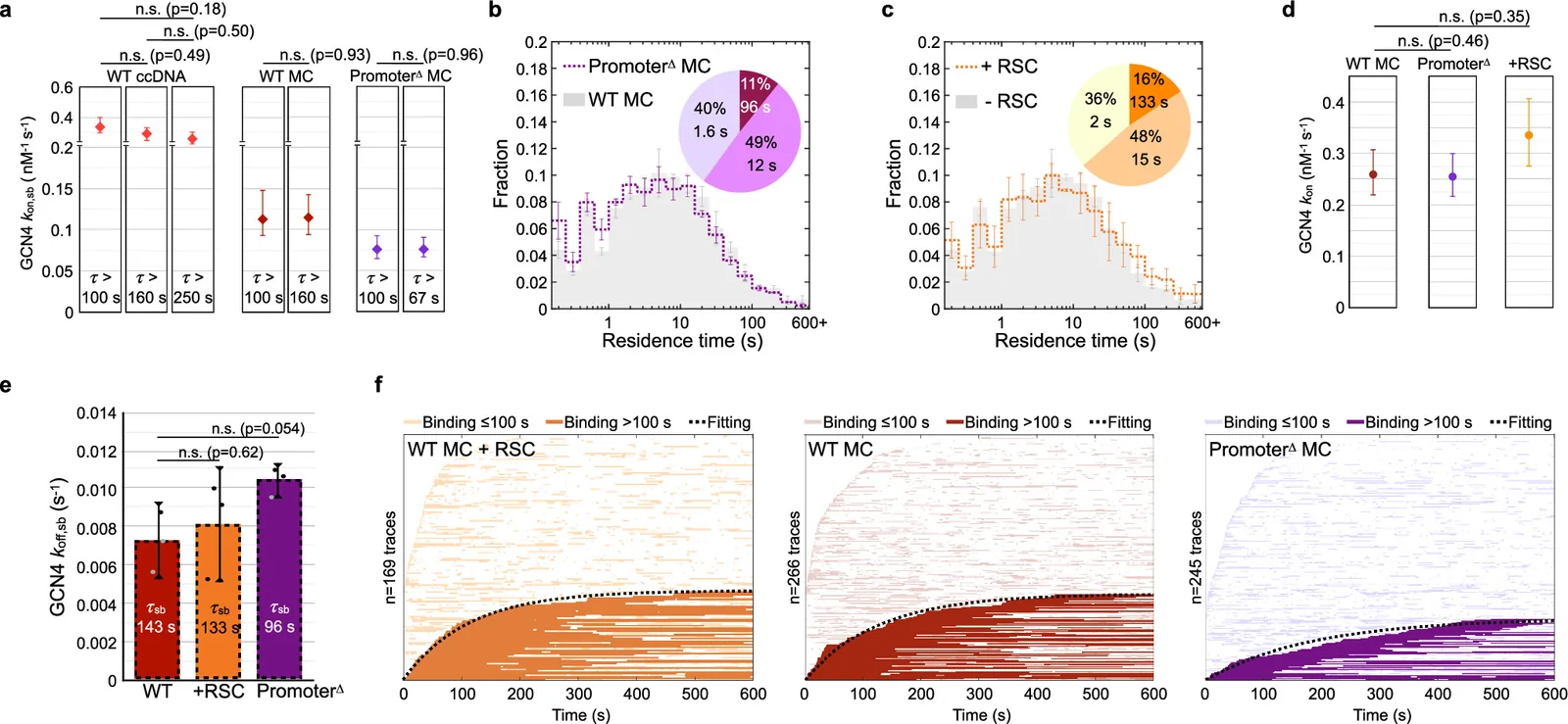
GCN4 *k*_on,sb_ under different *τ*_cutoff_, and *τ* comparisons for various MC templates. **a**, GCN4 *k*_on,sb_ values calculated using different τ_cutoff_ as indicated show little or no difference. Diamonds represent fitting values for original datasets, error bars indicate 95% C.I. determined by bootstrapping, and p-values are calculated by double-tailed Wilcoxon rank sum test. **b, c**, GCN4 τ histograms (averages of biological replicates) of promoter^∆^ MC (**b**) and RSC-treated WT MC (**c**). Error bars indicate S.D. among replicates. Inset pie charts represent corresponding 3-component exponential fit for GCN4 τ distribution. **d**, GCN4 *k*_on_ for WT, promoter^∆^, and RSC-treated MC, formats for statistics as in (**a**). **e**, GCN4 *k*_off,sb_ (τ_sb_ = 1/*k*_off,sb_ as indicated) on WT, RSC-treated WT, and promoter^∆^ MC. Columns and error bars indicate averages and S.D. among biological replicates. **f**, Comparison of GCN4 rastergrams on different MCs. Traces are sorted by first stable binding events (>100 s). All panels are derived from the same datasets as Fig. 4g–h. Source data

Furthermore, contracting the *HIS3* promoter NFR length from ~120 bp to ~30 bp through the deletion of promoter sequences responsible for NFR formation^16^ but keeping the UAS intact (promoter^∆^; Fig. 4e) led to a reduction in *k*_on,sb_ from 0.11 to 0.076 nM^−1^ s^−1^ (*P* = 0.012; Fig. 4g) but no reduction in the general *k*_on_ (Extended Data Fig. 6d). In contrast, expansion of the promoter NFR to ~200 bp through the action of the ATP-dependent chromatin remodeler RSC (Fig. 4f) resulted in a notable increase in GCN4 F_sb_ (9% to 16%, *P* = 0.018; Fig. 4h) while barely raising *k*_on,sb_ (0.11 nM^−1^ s^−1^ to 0.13 nM^−1^ s^−1^, *P* = 0.70; Fig. 4g), likely because of the 1D diffusion constraint. Hence, altering the *HIS3* promoter NFR length leads to corresponding changes in the UAS-specific association rate and the fraction of stable GCN4 binding to chromatin.

## AD accelerates GCN4 dissociation and association to enhance promoter targeting

**Fig. 5:**
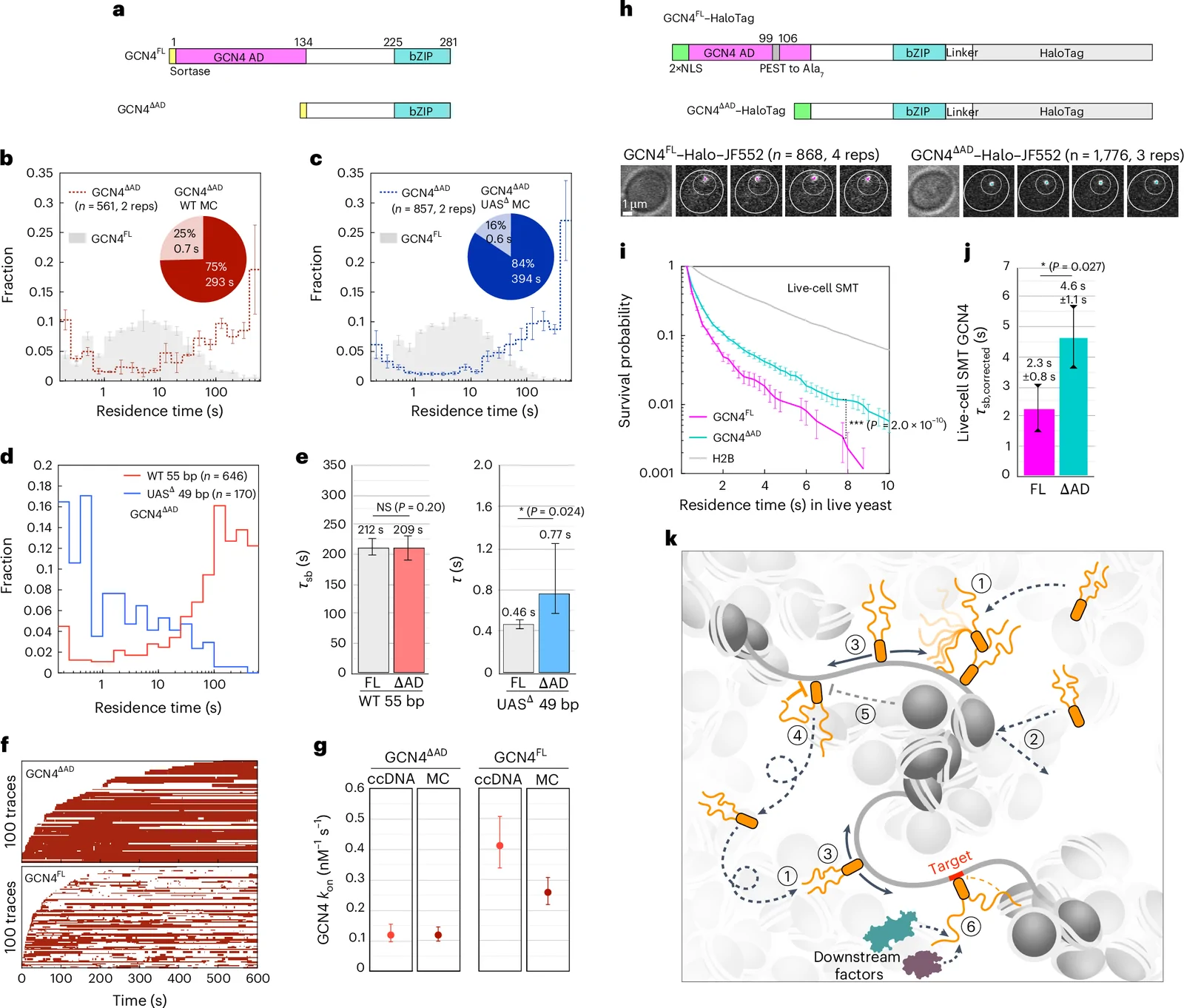
AD accelerates GCN4 dissociation and association to enhance target selectivity and search efficiency. **a**, GCN4 constructs for TIRF. **b**,**c**, GCN4^∆AD^ *τ* histogram for MC WT (**b**) and UAS^∆^ (**c**) (formats as in Fig. 4e). Gray shading represents the *τ* histogram of GCN4^FL^ on corresponding templates. Error bars indicate the s.d. among replicates. The numbers of binding events (*n*) and biological replicates are labeled. **d**, GCN4^∆AD^ *τ* histogram for linear 55-bp WT and 49-bp UAS^∆^ DNA. **e**, Comparison between GCN4^∆AD^ (same datasets as **d**) and GCN4^FL^ (same datasets as Fig. 2e,f) *τ*_sb_ on 55-bp WT DNA and major *τ* on 49-bp UAS^∆^ DNA (formats for statistics as in Fig. 2g). **f**, Rastergrams for GCN4^∆AD^ and GCN4^FL^ on WT MC, as in Fig. 1e. **g**, *k*_on_ of GCN4^∆AD^ (same datasets as **b**,**c**) and GCN4^FL^ (same as Fig. 4b) for different WT templates. Dots represent the fitting values of original datasets and error bars indicate the 95% CIs determined by bootstrapping. The numbers of analyzed traces for GCN4^∆AD^ were as follows: ccDNA, 54; MC, 90. **h**, GCN4 constructs for live-cell SMT. Representative trajectories shown with the numbers of trajectories (*n*) and biological replicates are labeled. **i**, Curves of 1 − CDF (mean of bootstraps) of *τ* for GCN4^FL^, GCN4^∆AD^ and H2B in live yeast. Error bars indicate the s.d. among bootstrapped data. *P* values were calculated using a double-tailed Wilcoxon rank-sum test. **j**, The *τ*_sb_ (mean of bootstraps) for GCN4^FL^ and GCN4^∆AD^ in live yeast, corrected by H2B *τ*_sb_. Error bars indicate the s.d. *P* values were calculated using a double-tailed *t*-test. **k**, Kinetic model of GCN4 target search on native chromatin. (1) AD accelerates GCN4 association. (2) Nucleosomes occlude association. (3) GCN4 1D diffusion is constrained within NFR by nucleosome barriers. (4) GCN4 AD undergoes autoinhibition. (5) Potential 3D steric occlusion exists from nearby nucleosomes. (6) Stable GCN4 occupancy at the UAS recruits downstream factors. Source data

**Extended Data Fig. 7:**
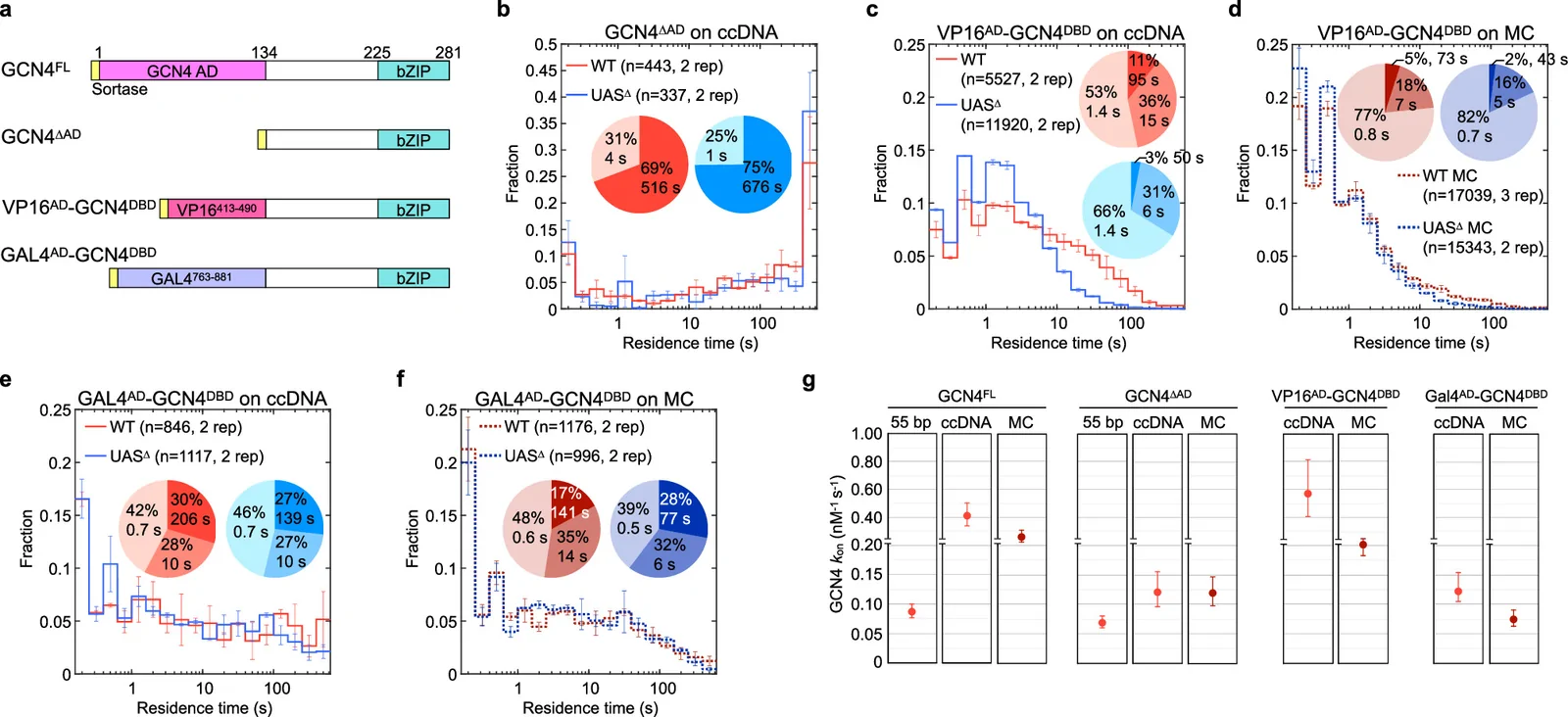
Binding kinetics of GCN4 constructs to short DNA, ccDNA, and MC. **a**, GCN4 constructs for TIRF. **b-f**, τ histogram (averages of replicates with error bars indicating S.D. among replicates) for GCN4^∆AD^ on ccDNA (**b**), VP16^AD^-GCN4^DBD^ on ccDNA (**c**) and MC (**d**), and GAL4^AD^-GCN4^DBD^ on ccDNA (**e**) and MC (**f**). Inset pie charts represent exponential fit. Numbers of binding events and biological replicates labeled. **g**, *k*_on_ of GCN4 constructs on indicated templates. Dots indicate fitting values of original datasets and error bars indicate 95% C.I. determined by bootstrapping. Numbers of analyzed traces: GCN4^∆AD^ on 55-bp, 138; ccDNA, 54; MC, 90. VP16^AD^-GCN4^DBD^ on ccDNA, 187; MC, 283. GAL4^AD^-GCN4^DBD^ on ccDNA, 129; MC, 96. We note that deletion of AD moderately compromises GCN4 dimerization based on fluorescence intensities. Such compromised dimerization should lead to shorter τ and therefore cannot explain the prolonged τ observed. Source data

Recent studies have revealed that, in addition to DBDs, IDRs or ADs in TFs contribute to genome-wide chromatin binding^26,27^. GCN4 possesses a defined AD, which is also an IDR, spanning residues 1–134 at the N terminus^39^. To examine the role of the GCN4 AD in promoter search, we measured binding of a truncated GCN4 (GCN4^∆AD^) to the MC (Fig. 5a). Compared to GCN4^FL^, the *τ* distribution showed a large increase in stable GCN4^∆AD^ binding to the WT MC (F_sb_ from 9% to 75%, *τ*_sb_ from 143 s to 293 s, *n* = 561; Fig. 5b) and a similar increase in stable GCN4^∆AD^ binding to WT ccDNA (Extended Data Fig. 7b). These findings indicate that the AD in GCN4^FL^ facilitates GCN4 dissociation from either chromatin or naked DNA templates.

Notably, GCN4^∆AD^ also displayed large F_sb_ (84%) and long *τ*_sb_ (394 s) on the UAS^∆^ MC (*n* = 857; Fig. 5c) comparable to (or even higher than) the values for the WT MC with UAS, indicating that the sequence specificity of GCN4^∆AD^ is compromised. Similar weakened specificity was also observed for ccDNA binding (Extended Data Fig. 7b). Such high and indistinguishable binding stabilities on WT and UAS^∆^ templates document that the GCN4 AD improves sequence specificity for the full-length TF by facilitating greater dissociation^40,41^ from off-target sequences than from the UAS.

However, for short (~50 bp) linear DNA fragments with little extra sequence, GCN4^∆AD^ exhibited ideal specificity for WT over UAS^∆^ templates (Fig. 5d,e), in full concurrence with the experimental history of sequence-specific DNA binding reported for bZIP DBDs^42^. Notably, although GCN4^FL^ and GCN4^∆AD^ displayed the same *τ*_sb_ on 55-bp WT DNA, the AD reduced the off-target *τ* (that is, increase *k*_off_) for GCN4 binding to 49-bp UAS^∆^ DNA (*P* = 0.024; Fig. 5e). These observations strengthen our conclusion that the GCN4 AD functions as a sequence filter for the cognate motif.

In addition, we found that the AD also increased the general *k*_on_ of GCN4^FL^ for both ccDNA and MC, compared to the rate for GCN4^∆AD^ (*P* < 0.001; Fig. 5f,g). Thus, the acceleration of both association and dissociation rates would shorten the GCN4 off-target search time, thereby improving the efficiency of target search (Fig. 5k). ADs from other TFs can, to various degrees, rescue the GCN4^∆AD^ deficiency for off-target dissociation and chromatin association (Extended Data Fig. 7 and ‘Discussion’).

## Live-cell single-molecule tracking (SMT) confirms the role of AD in driving GCN4 dynamics

To validate our in vitro observations in living cells, we carried out SMT for GCN4, which is subject to translational regulation and extremely rapid degradation^1,43^. To bypass these issues, we introduced mutations in the GCN4 PEST degradation signal and expressed the corresponding HaloTag fusions of GCN4^FL^ or GCN4^∆AD^ under the control of the weak *REV1p* promoter (Fig. 5h). SMT with long exposure time (250 ms) blurs fast-moving molecules, allowing the detection of slow-moving, chromatin-bound GCN4 (Fig. 5h). Deletion of the AD led to a longer *τ*_sb_ (Fig. 5i), which, after photobleaching correction, increased from 2.3 s for GCN4^FL^ to 4.6 s for GCN4^∆AD^ (*P* = 0.027; Fig. 5j). This twofold increase in residence time is consistent with our in vitro observations, although the absolute values of the average *τ* in live cells are far shorter than in vitro measurements, likely because of the absence of competing DNA (Extended Data Fig. 2d), other nuclear components or nucleosome movements^44^.

**Extended Data Fig. 8:**
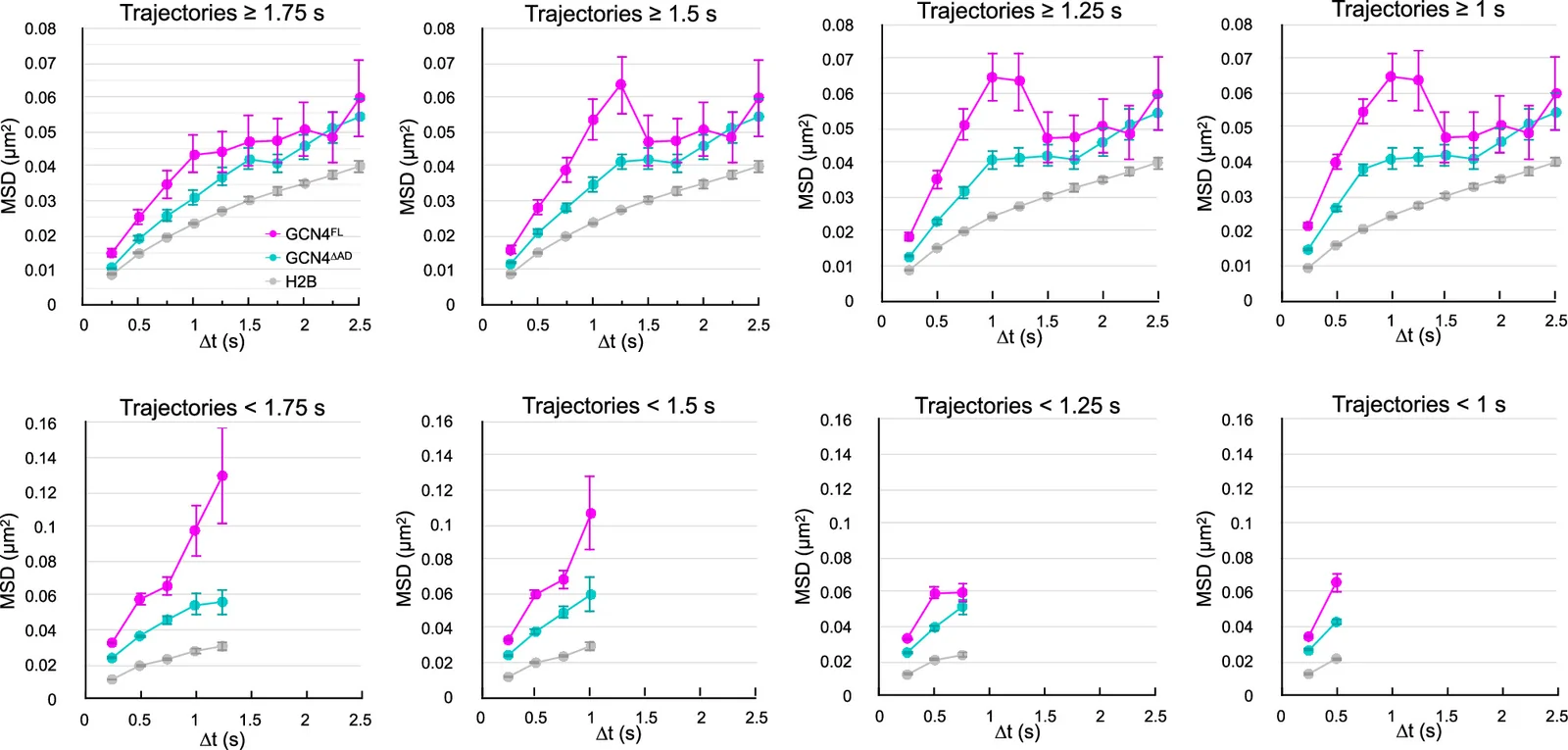
Live cell GCN4 MSD plots with different *τ*_cutoff_ values. Mean squared displacements (MSDs) of GCN4^FL^ and GCN4^∆AD^ for short or long trajectories in live cell SMT defined by different τ_cutoff_ values (same datasets as Fig. 5h–j). Error bars indicate S.D. among all analyzed trajectories. Source data

In addition, our in vitro observations of higher GCN4^FL^ dynamics mediated by the AD were consistent with mean squared displacement (MSD) analysis of live-cell SMT trajectories (Extended Data Fig. 8)^45^, where GCN4^FL^ displayed higher MSD than GCN4^∆AD^ for short trajectories (<1.75 s) but comparable MSD to GCN4^∆AD^ or H2B for long (≥1.75 s) trajectories. These data suggest that the AD is responsible for increased GCN4 mobility when bound nonspecifically. However, we do not rule out the possibility that increased mobility of GCN4^FL^ could also be because of higher chromatin motions caused by gene activation.

## Discussion

In this study, we developed an MC-based TIRF platform that allows single-molecule kinetic measurements of GCN4 targeting to the *HIS3* gene in native chromatin or its naked ccDNA equivalent. In addition to UAS-specific binding, we observed abundant and stable off-target GCN4 interactions on naked MC DNA, as shown by entrapment at degenerate motifs and length-dependent interactions on nonspecific sequences. These results point to a target search model of consecutive 1D diffusion and TF capture at degenerate and cognate sites on naked DNA, which is further demonstrated by the back-and-forth scanning of GCN4 between separate sites, similar to *E*. *coli* LacI^6^ and *Drosophila* GAGA factor^8^. Such 1D scanning by other eukaryotic TFs has also been proposed^24,25^.

Our observations on MC chromatin indicate that, in addition to direct occlusion at off-target sequences^9,10^, nucleosomes act as roadblocks^8^ constraining 1D diffusion to suppress TF promiscuity beyond NFRs. In support, both the *τ* distribution and the association rate of GCN4 for the 3.1-kb MC were intermediate to the values for 150-bp and 280-bp linear DNA fragments (Fig. 4b,c and Extended Data Fig. 5g). However, we do not rule out the likelihood that higher-order chromatin organization also occludes promiscuous binding, because GCN4 binding at two half-sites within the HIS3 3′ NFR (Extended Data Fig. 3g–i) was also largely suppressed on the MC as previously shown (Extended Data Fig. 3k)^33^ and confirmed in this work (Fig. 3b,c and Extended Data Fig. 3d–h). In addition, nucleosomes near NFRs in 3D space might also destabilize GCN4 binding (Fig. 5k), as hinted by a decrease in *τ*_sb_ on WT 122N6 nucleosomes compared to bare DNA (Extended Data Fig. 5i), as well as shorter *τ*_sb_ for WT MC compared to linear NFR DNA fragments (albeit with statistical confidence). Our observations are also consistent with a report of reduced residence time on reconstituted nucleosome arrays for yeast Rap1 (ref. ^46^).

Altogether, drawing on the original facilitated diffusion model for TF search on bacterial DNA^4^, we extended the same principles to eukaryotic chromatin, whereby a TF lands on exposed NFRs from 3D diffusion, undergoes nucleosome-restricted 1D scanning within each NFR and is captured by its cognate target or dissociates (Fig. 5k). Although some TFs, including several bHLH–bZIP factors, display preferential binding to nucleosomal DNA at certain SHL positions^9,47–49^, their target search processes might still include nucleosome-restricted 1D diffusion, as shown for the GAGA factor^8^. Interestingly, the 1D diffusion range of GCN4 between 103 bp and 233 bp, which is comparable to the <115 bp reported for LacI^5^, approximates average NFR lengths (~140 bp) in the yeast genome^13,15,16^, suggesting that the NFR length may represent an evolutionary trade-off between suppressing promiscuous binding and permitting productive on-target association. NFR sizes comparable to the 1D diffusion range also allow efficient regulation of the TF association rate by chromatin remodeling activities as shown here (Fig. 4g,h) and elsewhere^50^.

It is interesting that *k*_on,sb_ for ccDNA substantially exceeded the upper limit for short linear fragments. Beyond the inclusion of stable, off-target binding events, we speculate that the elevated *k*_on,sb_ could be because of the higher spatial DNA density of a supercoiled template (Extended Data Fig. 1b). Futures studies on supercoiled and relaxed ccDNA and MC should provide insights into the influence of DNA topoisomerases on the kinetics of TF target search.

A recent study^27^ revealed that many TFs including GCN4 display a large fraction of non-functional binding throughout the genome. This is consistent with our observation that 91% of GCN4 binding on WT MC is not at the UAS. However, as we showed, the duration of such promiscuous binding is limited by nucleosome organization, thus disfavoring ectopic functions. The suppression of promiscuous binding observed for GCN4 should apply to other TFs for which screening from synthetic DNA libraries reveals large numbers of potential off-target binding sequences^24,25,51^. Such off-target binding of TFs may activate cryptic transcription from intragenic regions, as shown for GCN4 and its human homolog AP1 (refs. ^33,52^). Disruptions of intragenic nucleosome density, phasing and modifications are known to derepress transcription from cryptic promoters^52–55^. Thus, the establishment and maintenance of NFRs over eukaryotic regulatory elements and the coverage by nucleosome arrays over gene bodies and extragenic DNAs are both important for proper transcriptional regulation.

In addition to the influence of chromatin, we found that ADs (or IDRs) facilitate GCN4 dissociation from nonspecific sequences. Facilitated TF dissociation^40,41^ or autoinhibition of DBDs by IDRs has been reported for other proteins such as Sox2, p53 and MYC:MAX^36,56–58^. GCN4 might adopt a similar mechanism, whereby its acidic IDR competes with DNA for the bZIP domain and improves sequence selectivity by outcompeting lower-affinity DNA sequences (Fig. 5k). We found that the GCN4 IDR can also elevate the association rate in vitro and increase local mobility of chromatin-bound factors in living cells, potentially by increasing the effective radius of diffusion and/or interacting with chromatin directly^59^. These effects are observed for other TF ADs or IDRs, as substitution of VP16^AD^ restores sequence binding specificity and the association rate of GCN4^∆AD^ (Extended Data Fig. 7a–d,g). However, distinct from GCN4 and VP16, GAL4^AD^, while also reducing the prolonged residence time of GCN4^∆AD^, poorly facilitated specific binding or the association rate (Extended Data Figs. 7e–g). Other examples where IDRs modulate TF kinetics differently from GCN4 and VP16 ADs^36,38,45,60–63^ indicate alternative roles for ADs and IDRs in TF target search.

Recent studies revealed that ADs and IDRs expand the repertoire of TF targets as measured by ChEC-seq^26,27^. We suggest that this is not in conflict with our findings. The dynamic nature of GCN4^FL^ allows high-frequency sampling of chromatin targets, potentially generating more ChEC-seq peaks despite short-lived GCN4 residency at each site. In contrast, stable trapping of GCN4^∆AD^ would lower sampling frequency and produce fewer ChEC-seq peaks, as observed. Separately, IDR-directed TF binding could be mediated by protein partners, whose influence we did not investigate in this work.

Lastly, the yeast MC is a versatile substrate that bridges physiological chromatin in vivo and reconstituted chromatin in vitro. It allows for convenient genetic manipulations and additional biochemistry to investigate histone modifications^30^ and histone variants and can be extended to single-molecule kinetic analyses of transcription preinitiation through termination. The MC platform is also adaptable to crude nuclear extracts that desirably mimic the nucleoplasmic environment^64^. With its origin of replication, the MC may also be deployed as a native chromatin substrate to study the kinetics of other pathways of DNA metabolism.

## Methods

### Plasmids and yeast strains

To construct TATO7LO6–HIS3, we used an MC backbone similar to Unnikrishnan et al.^65^ but replaced the 8× LacO with 6×LacO + 7×TetO. We inserted the *HIS3* gene (sacCer3 ChrXV: 721,778–722,735) into the BamHI site in the backbone. To construct UAS^∆^, we deleted 6 bp (TGACTC) from the UAS. For UAS^∆^scr, we further mutated all five half-sites (ATGAC) into AGCTT, as well as degenerate motifs ATGAGTCGT (within *TRP1* ORF) into ACGAAAGTT, GTGACACAT into AGCTTAACT and TTGCCTCAT into AGCTTAGCT. To construct promoter^∆^, we deleted 90 bp (sacCer3 ChrXV: 721,795–721,884) except for the UAS sequence (ATGACTCTT). The MC sequence was cloned into the XbaI site on pUC19 backbone and amplified in *E*. *coli*.

For yeast MC strains, we used *HTB2*–*GFP(S65T)* W1588-4c (*MATa*, *ade2-1*, *can1-100*, *ura3-1*, *his3-11,15*, *leu2-3,112*, *trp1-1*, *RAD5*) as our background strain. MC sequences were excised out from the plasmid, relooped by T4 ligation and transformed into the *HTB2*–*GFP* strain. Transformed yeast was selected on CSM −Trp plates. For each yeast MC strain in this study, we sequenced the purified MC ccDNA to verify that it carries the correct monomer loop.

For live-cell SMT strains, we used *pdr5∆* W303 as our background strain and integrated the *REV1p::2×NLS*–*GCN4*^*FL*^ (or *GCN4*^*∆AD*^)–*6×GGGGS*–*Halo* construct into the endogenous *URA3* locus. Transfected yeast was selected on CSM-Ura plate and we confirmed the correct integrations by colony PCR and sequencing.

### Linear DNA fragment and reconstituted nucleosome acquisition

For 55-bp WT linear DNA (CATTTTTTTTTTTCCACCTAGCGGATGACTCTTTTTTT TTCTTAGCGATTGGCAT), 49-bp UAS^∆^ linear DNA (CATTTTTTTTTTTCCACCTAGCG GATTTTTTTTTCTTAGCGATTGGCAT) and 53-bp UAS^half^ linear DNA (CATTTTTTTT TTTCCACCTAGCGGATGACTTTTTTTTTCTTAGCGATTGGCAT), we ordered Alexa Fluor 488-5′-positive strand and biotin-TEG-5′-negative strand from Integrated DNA Technologies (IDT). Two strands (2 µM each) were annealed in buffer (20 mM HEPES–KOH pH 7.6, 10 mM MgSO_4_, 80 mM potassium acetate, 5% glycerol and 0.2 mg ml^−1^ BSA) using the following protocol: 95 °C, 5 min, −0.1 °C s^−1^ to 70 °C, 70 °C 30 min, −0.1 °C s^−1^ to 20 °C. Annealed DNA was stored at 4 °C and ready for TIRF microscopy.

For other linear DNAs, we ordered Alexa Fluor 488-5′-CGGATCCTTATGCCTCGGTAATG (forward primer) and biotin-TEG-5′-reverse primer (matching corresponding position in MC sequence) from IDT for PCR. PCR product is checked by agarose gel electrophoresis and the linear DNA fragment was purified using a Monarch Spin PCR DNA cleanup kit (New England Biolabs (NEB), T1130). Purified DNA was stored at 4 °C and ready for TIRF microscopy.

For dual-labeled DNA used in three-color FRET experiments, we ordered forward UAS oligo (CATTTTTTTTTTTCCACCTAGCGGATGACTCT TTTTT/iCy5N/TTCTTAGCGATTGGCAT), reverse UAS oligo (/5Phos/CTAGATGCCAATCGCTAAGAAAAAAAAAGAGTCATCCGCTAGGTGGAAAAAAAAAAATG), forward half-site oligo (/5Phos/CTAGTGAGCAGGCAAGATAAACGAAGGAAAAGATGACAGAGCAGAAAGCCCTAGTAAAGCGTA) and reverse half-site oligo (/5BioTinTEG/TACGCT TTACTAGGGCTTTCTGCTCTGTCATCTTT/iCy7N/CCTTCGTTTATCTTGCCTGCTCA) from IDT. Corresponding forward and reverse oligos were annealed and then two double-stranded DNA fragments were ligated by T4 ligase to form the final construct.

To construct the *HIS3*–601 (half-site) sequence, we inserted 122 bp from MC (or 116 bp for UAS^∆^, CGGATCCTTATGCCTCGGTAATGATTTTCAT TTTTTTTTTTCCACCTAGCGGATGACTCTTTTTTTTTCTTAGCGATTGGCATTATCACATAATGAATTATACATTATATAAAGTAATGTGA) upstream of the Widom 601 sequence^66^ in pCR2.1-601 plasmid. A half-site (ATGAC) was engineered into Widom 601 sequence corresponding to the +24 (relative to +1 TSS) half-site in *HIS3*. PCR and purification were conducted as described above. Nucleosome reconstitution and purification were performed as described previously^8^. *Xenopus* *laevis* H2A/H2B dimers (with Cy5-labeled H2B K105C mutant) and H3/H4 tetramers were generous gifts from the G. D. Bowman lab.

### Purification of GCN4 variants

The 6×His–SUMO–GGG–GCN4 variants (GCN4^FL^, GCN4^∆AD^, VP16^AD^–GCN4^DBD^ and GAL4^AD^–GCN4^DBD^) were expressed from pET plasmids. Transformed *E*. *coli* (Rosetta 2 DE3; Novagen, Sigma-Aldrich, 71397) was induced to express the proteins by adding 1 mM IPTG (Sigma-Aldrich, I6758) for 3 h, collected and resuspended in BT-450 (50 mM Tris-HCl pH 7.5, 450 mM NaCl, 5 mM β-mercaptoethanol (β-ME), with protease inhibitor cocktail (PI) including a final concentration of 2.5 µg ml^−1^ leupeptin, 1.5 µg ml^−1^ pepstatin A, 0.1 mM PMSF, 2 mM benzamidine HCl and 10 µg ml^−1^ each TPCK and TLCK). Bacteria were lysed by sonication (Bioruptor 300, Diagenode, ‘H’ level, 30 s on, 30 s off, 60 cycles; Sonicator S-4000, Misonix, with microtip, 10 s on, 50 s off, 18 cycles) and lysate was centrifuged at 15,000 rpm (27,143.1*g*), 4 °C for 30 min (JA-20 rotor; Beckman Coulter, 334831).

We applied lysates to Ni Sepharose 6 Fast Flow resin (Cytiva, 17531801) and eluted the proteins into BTN-500 (50 mM Tris-HCl, 500 mM NaCl, 0.1% NP-40, 5% glycerol, 500 mM imidazole and 5 mM β-ME, with PI added). Buffer was exchanged into BT-300 (50 mM Tris-HCl pH 7.5, 5% glycerol, 0.05% NP-40, 300 mM NaCl and 5 mM β-ME, without PI) using PD-10 desalting columns (Cytiva, 17085101), the final volume was adjusted to 10 ml by adding BT-300 and then 76.5 µg of 6×His–SUMO protease (lab stock) was added for overnight incubation at 4 °C.

We removed 6×His–SUMO protease and free SUMO tag by another round of Ni Sepharose column purification as described above but collected flowthrough this time and diluted its NaCl concentration to 150 mM using BT-0 (same as BT-300 but without NaCl). Proteins were applied to Q Sepharose Fast Flow (for GCN4^FL^ and VP16^AD^–GCN4^DBD^; Cytiva, 17051001) or Heparin Sepharose 6 Fast Flow (for GCN4^∆AD^ and GAL4^AD^–GCN4^DBD^; Cytiva, 17099801) and proteins were eluted into BT-400, BT-600, BT-800 and BT-1000 (same as BT-300 but with indicated concentrations of NaCl and 1 mM DTT instead of β-ME, without PI). Target fractions were combined and concentrated by an Amicon Ultra centrifugal filter (10-kDa molecular weight cutoff; Millipore Sigma, UFC9010). After final concentration measurement, GGG–GCN4 variants were frozen in liquid nitrogen and stored at −80 °C.

### Purification of LacI–SNAP and adaptor protein

LacI (residues 1–349; deleting the last 11 residues abolishes its tetramerization ability^67^)–GGGSGGG–SNAPtag–6×His was expressed from pRSF plasmid with lacO deleted and purified using a Ni Sepharose column as described above.

Adaptor protein (NLS–TetR–GR6–TAP (SBP–2×TEV site–protein G–6×His)^68,69^) was expressed from pRSF plasmid. Cell lysis, Ni Sepharose column purification, Heparin Sepharose column purification and ultra centrifugal filter concentration were conducted as described above. Purified protein was exchanged to BE-300 (20 mM HEPES–KOH pH 7.6, 0.25 mM EDTA, 0.05% NP-40, 10% glycerol, 300 mM potassium acetate pH 7.6 and 1 mM DTT, with PI added) and frozen.

### Protein labeling

We labeled GCN4 variants by Sortase A5 (ref. ^70^). The labeling reaction included 6.5 mM Tris-HCl pH 7.5, 300 mM NaCl, 1 mM CaCl_2_, 10 mM MgCl_2_, 6.7 µM Sortase A5 (Active Motif, 13100), 1.3 µM GGG–GCN4 variant and 267 µM TAMRA–LPETGG peptide (Innovagen, SP-5364-1) in a total volume of 750 µl. The reaction mix was incubated at room temperature for 1 h and then quenched by adding 32 µl of 0.5 M EDTA. The quenched reaction was applied to a Heparin Sepharose column and free fluorescent peptide was washed away with HSB-150 (20 mM HEPES–KOH pH 7.6, 1 mM EDTA, 10% glycerol, 0.1% NP-40, 150 mM NaCl and 1 mM DTT, with PI added). Labeled GCN4 variants were eluted with HSB-1000 (same as HSB-150 but with 1 M NaCl) and exchanged into BE-300. The concentration of labeled protein was measured by SDS–PAGE (scanning the gel in the Cy3 channel using an Amersham Typhoon 5 biomolecular imager; Cytiva, 29187191), with quantification by ImageStudio (LICORbio) using TAMRA–peptide as the fluorescence standard. Freezing and storage were performed as described above.

For three-color FRET experiments, GCN4–LPETGG was purified as described earlier. Labeling was carried out using GGG–Cy3 (Vector Labs, CCT-1584-1) as described above.

Next, ~3.5 nmol of LacI–SNAP was labeled in 1 ml of BT-300 (same as BT-100 but with 300 mM NaCl) containing 0.2 mg ml^−1^ BSA and 10 µM SNAP–Surface Alexa Fluor 488 (NEB, S9129). The reaction was incubated at room temperature for 2 h and the buffer was exchanged into BN-5 (same as BTN-500 but with 150 mM NaCl and 5 mM imidazole). Protein was applied to a Ni Sepharose column and free dye was removed by BN-5 washing. Labeled LacI–SNAP was eluted with BN-500 (same as BN-5 but with 500 mM imidazole). Subsequent buffer exchange, concentration quantification and storage were conducted as described above.

### MC purification

Yeast carrying MC is cultured in 1 L of CSM −Trp medium at 30 °C until the optical density at 660 nm (OD_660_) reached 1–1.5. Yeast cells were collected as described above and resuspended in BH-150 (50 mM HEPES–KOH pH 7.6, 0.1 mM EDTA, 0.2 mM EGTA, 2 mM MgCl_2_, 5% glycerol, 0.1% Tween-20, 150 mM KCl and 1 mM DTT, with PI added). The final volume was adjusted to 5 ml per 0.8–1 g of cells. Cells were lysed with zirconia/sillica beads (0.5 mm in diameter; BioSpec, 11079105) in a mini-beadbeater-96 (BioSpec, 1001) for 3 min 2 s for two cycles, with a 9-min chilling at −20 °C between beating cycles. Lysed cell debris was pelleted at 27,000 rpm (124,668.3*g*), 4 °C, for 95 min (SW41-Ti rotor (Beckman Coulter, 331362) and L8-70 ultracentrifuge (Beckman Coulter)). Supernatant was collected as whole-cell extract (WCE).

We prepared 75 µl of IgG Dynabeads for every gram of yeast cells (IgG Dynabeads prepared using purified rabbit IgG (MP Biomedical, 55944) and Dynabeads M-270 Epoxy (Invitrogen, 14301)). IgG Dynabeads were washed in BH-150, mixed with adaptor protein (0.14 nmol of adaptor per 100 µl of IgG Dynabeads used, also including 0.2 mg ml^−1^ BSA during incubation) and incubated while slowly rotating for 1 hr at 4 °C. Then, IgG Dynabeads were washed three times again in BH-150, mixed with WCE and slowly rotated for 2 h at 4 °C. The Dynabeads were then washed three times in BH-200 (same as BH-150 but with 200 mM KCl; starting from this step, all buffers contained 0.2 mg ml^−1^ BSA without adding PI) followed by three washing steps in BH-150. MC was eluted from beads by TEV digestion; the beads were resuspended in BH-150 (for every L of yeast culture equivalent, we used 600 µl of BH-150 + 20 µl of IceTEV protease (Sigma-Aldrich, SAE0195)) and slowly rotated for 2 h at 4 °C. The supernatant was collected and 10% (v/v) pure glycerol was added. Aliquot, freezing and storage were as described above. One aliquot was used to examine the final adaptor protein concentration by SDS–PAGE.

For RSC-treated MC, IgG Dynabeads were washed in remodeling buffer (25 mM HEPES–KOH pH 7.6, 150 mM KCl, 10 mM MgCl_2_, 9% glycerol, 0.02% NP40 and 124 µg ml^−1^ BSA) right after WCE incubation. The reaction was conducted in remodeling buffer with 2 mM ATP and ~32 nM RSC remodeler purified from yeast (estimated RSC-to-nucleosome molar ratio of 1:10, with RSC purified as described previously^71^) and proceeded for 10 min at room temperature. The Dynabeads were then washed with BH-200 four times in the presence of ATP to get rid of the remaining RSC. Subsequent steps were as described above.

### MC MNase-seq

For every batch of MC that needed MNase-seq, we aliquoted one third of the beads after the three BH-200 washing steps (described above) for MNase-seq. Beads carrying MC were further washed three times in ExB6.5/70 (10 mM HEPES–KOH pH 7.6, 0.5 mM EGTA, 6.5 mM MgCl_2_, 10% glycerol, 70 mM KCl, 1 mM DTT and 0.2 mg ml^−1^ BSA) and resuspended in 64 µl of ExB6.5/70. Next, 6 U of MNase (Nuclease S7, Roche, 10107921001) and 1.33 µl of 0.1 M CaCl_2_ were added to the reaction. The beads were slowly rotated at room temperature for 15 min and the reaction was quenched by adding 27 µl of STOP buffer (100 mM EDTA and 2.5% sarkosyl). Then, 333 µl of SDS mix (20 mM EDTA, 200 mM NaCl and 1% SDS) and 7 µl of proteinase K (Invitrogen, AM2548) were further added to the beads, which were slowly rotated at 37 °C overnight. DNA fragments were purified from supernatant using a Monarch Spin PCR DNA cleanup kit (NEB, T1130). We applied 1 µl of sample on agarose gel to check digestion efficiency (major band at ~150 bp) and sent the rest for sequencing (Plasmidsaurus, premium PCR service).

### ccDNA purification

We proceeded with the MC purification protocol to three BH-150 washing steps before TEV digestion as described above. The beads were directly resuspended in 1 ml of SDS mix with 21 µl of proteinase K (for 1 L of yeast culture equivalent) and slowly rotated at 37 °C overnight; ccDNA was purified using a Monarch Spin PCR DNA cleanup kit (NEB, T1130) from the supernatant.

### PEG-passivated TIRF slide preparation

We followed the Park et al.^72^ protocol with some modifications. Quartz slides (G. Finkenbeiner; 1′ × 3′ × 1 mm) were drilled and put in Coplin jars together with briefly flamed coverslips (VWR, 48393-230; 40 × 24 mm). Slides and coverslips were washed with MilliQ water, Piranha-etched for 30 min, washed with MilliQ water again, sonicated in methanol for 30 min twice and sonicated in 1.5 M KOH for 40 min. KOH was thoroughly washed away with MilliQ water (ten washes) and any moisture was removed by extensive methanol washing (five washes). Next, 100 ml of methanol, 5 ml of glacial acetic acid and 2 ml of *N*-(2-aminoethyl)-3-aminopropyltrimethoxysilane (UCT, A0700) were added into each jar before incubating in the dark for 30 min. The jar was gently shaken 3–4 times during incubation. The slides and coverslips were thoroughly washed with methanol and then with water, dried with air dusters and put in a wet box. Next, 10 mg of biotin-PEG-SVA (molecular weight: 5,000; Laysan Bio) and 190 mg of mPEG-SVA (molecular weight: 5,000; Laysan Bio) were dissolved in ~2 ml of 0.1 M NaHCO_3_ and 0.55 M K_2_SO_4_ water solution. The volume was carefully adjusted to just above the PEG cloud point^72^ and 100 µl of PEG solution was immediately sandwiched between each slide and coverslip. The slides were incubated in dark for 14–16 h, washed with water and dried thoroughly before being placed in 50-ml Falcon tubes sealed in vacuum bags. Passivated slides and coverslips were stored at −20 °C.

### TIRF microscope setup

We built a prism-type TIRF microscope on a Zeiss AXIO Observer inverted fluorescence microscope with a Zeiss C-Apochromat ×63 water-immersion Korr objective (421787-9970) following a blueprint provided by T. Ha. The laser source was a Stradus Versalase multiple-wavelength laser system (Vortran). Images were projected to a Hamamatsu electron-multiplying charge-coupled device camera (C9100-13) controlled by TriggerscopeMM-2.0gama software. One pixel length corresponded to 161 nm in imaged samples.

### Immobilization of ccDNA, MC and linear DNA or reconstituted nucleosomes

All flow-in operations were performed using a Syringe pump (Chemyx, Fusion 200; withdraw rate: 1.1 ml min^−1^) through tubes (Weico Wire, ETT-26). For ccDNA, 25 pmol of adaptor proteins were digested using 0.6 µl of IceTEV protease in 100 µl of B-60 (50 mM HEPES–KOH pH 7.6, 0.2 mM EDTA, 5 mM MgCl_2_, 5% glycerol and 60 mM KCl) with 0.5 mM DTT and 0.2 mg ml^−1^ BSA at 4 °C for at least 30 min before immobilization. First, 100 µl of 0.5 mg ml^−1^ streptavidin (in B-60) was added to the imaging chamber and incubated for 90 s. Second, the chamber was washed with 200 µl of B-60. Third, 0.83 µl of digested adaptor protein, ~80 ng of ccDNA, ~1.2 pmol of LacI–SNAP–AF488 and 100 µg of BSA (Ultrapure, Invitrogen, AM2616) were mixed in a small volume (<5 µl), incubated on ice for 3 min, topped up to 100 µl with B-60, then added into an imaging chamber and incubated for another 5 min. Fourth, 50 µl of B-60 + 0.2 mg ml^−1^ BSA was added to the chamber, followed by 50 µl of imaging buffer (10 mM HEPES–KOH pH 7.6, 2 mM MgSO_4_, 150 mM potassium acetate, 5% glycerol, 0.2 mg ml^−1^ Ultrapure BSA, 1 mg ml^−1^ Trolox adjusted to pH ~7 with KOH, 0.77 mg ml^−1^ protocatechuic acid (MP Biomedical, 99-50-3) and 0.9 U per ml protocatechuate-3,4-dioxygenase (MP Biomedical, 9029-47-4)).

The protocol for MC was the same except for the third step, where MC stock was instead diluted with B-60 + BSA to ensure that the adaptor protein concentration was equal to that for ccDNA. Next, 100 µl of diluted MC was added into the chamber and incubated for 5 min.

For linear DNA or reconstituted nucleosomes, in the first step, we used neutravidin instead of streptavidin and incubated for 50 s; in the third step, linear DNA or nucleosome was diluted to ~20 pM in B-60 and incubated for 60 s. The remaining steps were the same as above.

### TIRF video recording

We prepared imaging mix (0.1 nM labeled GCN4 variant in 100 µl of imaging buffer as described above) at room temperature. The frame rate was 10 fps (100 ms of exposure time) for all videos. For the first video in each chamber, we performed a ten-frame 488-nm laser (5 mW) flash, switched off the laser and turned on the 561-nm laser (3 mW) while flowing in the imaging mix. We collected 6,000 frames with the 561-nm laser on and then performed another ten-frame 488-nm laser flash before ending the video. After the first video, 4–5 videos (ten-frame 488-nm flash, 6,000 frames with the 561-nm laser and another 488-nm flash) were collected from different fields of view in the same imaging chamber.

For Cy5 nucleosome experiments, extra ten-frame flashes with a 639-nm laser (5 mW) were performed at the beginning and the end of each video.

For three-color FRET videos, the protocol was slightly modified. The 561-nm laser power was 5 mW and ten-frame flashes with a 639-nm laser (5 mW) and 750-nm laser (15 mW) were performed at the beginning and the end of each video.

### Live-cell SMT

We followed the Ling et al.^73^ protocol with some modifications. Yeast was maintained in log phase (OD_660_ ≤ 0.8) in CSM −Ura medium for at least 2 days before imaging, then inoculated into 2.5 ml of CSM medium containing 250 nM JF552–HaloTag ligand and cultured at 30 °C, 220 rpm, overnight. We controlled the initial OD_660_ such that it did not exceed 0.8. On the second day, the yeast culture was diluted to OD_660_ = 0.2 (extra dye added to maintain 250 nM) and further cultured for ~5 h. Yeast cells were collected by centrifuge at 3,000*g* for 5 min and washed with CSM medium eight times. We further cultured the yeast in 2.5 ml of fresh CSM medium without dye for 30 min, collected the cells and washed another ten times with CSM. Cells were adhered to the coverslip in a cell chamber, washed with 500 µl of CSM three times, left to sit in CSM for 5 min and washed another three times before imaging. The imaging setup included a 5-mW 554-nm laser with a frame rate of 4 fps (250 ms of exposure time).

### Data analysis and quantification

#### Statistical information

For ccDNA and MC, the numbers of biological replicates (purified from independent yeast cultures) and total binding events (*n*) used for calculating residence time (Figs. 2–5 and Extended Data Figs. 2–5) were as follows:

GCN4 (GCN4^FL^) on WT ccDNA, four replicates, *n* = 1,021; UAS^∆^ ccDNA, three replicates, *n* = 1,468; UAS^∆^scr ccDNA, two replicates, *n* = 1,589; WT MC, three replicates, *n* = 2,738; UAS^∆^ MC, three replicates, *n* = 6,131; UAS^∆^scr MC, two replicates, *n* = 7,499; Promoter^∆^ MC, three replicates, *n* = 5,614; RSC-treated MC, three replicates, *n* = 4,170.

GCN4^∆AD^ on WT ccDNA, two replicates, *n* = 443; UAS^∆^ ccDNA, two replicates, *n* = 337; WT MC, two replicates, *n* = 561; UAS^∆^ MC, two replicates, *n* = 857.

VP16^AD^–GCN4^DBD^ on WT ccDNA, two replicates, *n* = 5,527; UAS^∆^ ccDNA, two replicates, *n* = 11,920; WT MC, three replicates, *n* = 17,039; UAS^∆^ MC, two replicates, *n* = 15,343.

GAL4^AD^–GCN4^DBD^ on WT ccDNA, two replicates, *n* = 846; UAS^∆^ ccDNA, two replicates, *n* = 1,117; WT MC, two replicates, *n* = 1,176; UAS^∆^ MC, two replicates, *n* = 996.

Exponential fitting was carried out for each replicate individually and the mean values of all replicates are shown in the figures. For these data, the s.d. (error bars) and double-tailed *t*-tests were calculated among replicates (Figs. 2–5, Extended Data Figs. 2–5).

For linear DNA fragments and reconstituted nucleosomes, there were no biological replicates as they were synthesized; accordingly, we pooled together all data from different experiments and the total numbers of binding events for residence time calculations (Figs. 2, 3 and 5 and Extended Data Figs. 2, 3 and 5) were as follows:

GCN4 (GCN4^FL^) on WT 55-bp, *n* = 1,213; WT 156-bp, *n* = 1,282; WT 286-bp, *n* = 739; UAS^∆^ 49-bp, *n* = 373; UAS^∆^ 150-bp, *n* = 3,098; UAS^∆^ 280-bp, *n* = 2,333; UAS^half^ 53-bp, *n* = 1,452; UAS^∆^scr 280-bp, *n* = 5,049; WT HIS3–601 (half-site) 275-bp, *n* = 2160; WT–601 (half-site) nucleosome (122N6), *n* = 2,121; UAS^∆^–601 (half-site) 269-bp DNA, *n* = 8,542; UAS^∆^–601 (half-site) nucleosome (116N6), *n* = 6,414; UAS^∆^–601 (no half-site) 269-bp DNA, *n* = 12,632; UAS^∆^–601 (no half-site) nucleosome (116N6), *n* = 2,687.

GCN4^∆AD^ on WT 55-bp, *n* = 646; UAS^∆^ 49-bp, *n* = 170.

We use bootstrapping method with 200 resampling to determine 95% confidence intervals (CIs; error bars) of exponential fitting parameter values. For these data, double-tailed *t*-tests were carried out on bootstrapping results assuming df = 1 for stringent comparisons.

When analyzing association rates (*k*_on_ and *k*_on,sb_; Figs. 4 and 5 and Extended Data Figs. 4 and 5), we only used the ‘flow-in’ videos in each imaging session. We pooled together flow-in traces from all experiments and used a bootstrapping method with 200 resampling to determine 95% CIs (error bars). Total numbers of flow-in traces were as follows:

GCN4 (GCN4^FL^) on WT 55-bp, *n* = 232; WT 156-bp, *n* = 279; WT 286-bp, *n* = 203; WT 587-bp, *n* = 294; WT–601 (half-site) nucleosome (122N6), *n* = 266; WT ccDNA, *n* = 396; WT MC, *n* = 266; Promoter^∆^ MC, *n* = 245; RSC-treated MC, *n* = 169.

GCN4^∆AD^ on WT 55-bp, *n* = 138; WT ccDNA, *n* = 54; WT MC, *n* = 90.

VP16^AD^–GCN4^DBD^ on WT ccDNA, *n* = 187; WT MC, *n* = 283.

GAL4^AD^–GCN4^DBD^ on WT ccDNA, *n* = 129; WT MC, *n* = 96.

For comparisons of association rates (Figs. 4 and 5 and Extended Data Figs. 4 and 5), we carried out Wilcoxon rank-sum tests for corrected on-times (time intervals between protein flow-in and first binding events/first stable binding events multiplied with protein concentrations).

For live-cell SMT (Fig. 5 and Extended Data Fig. 8), we pooled together all data from four independent imaging sessions and collected a total of 868 trajectories for GCN4^FL^ and 1,776 trajectories for GCN4^∆AD^.

For MNase-seq, averages of two biological replicates (independent yeast culture) are shown for each MC nucleosome map (Figs. 1, 3 and 4 and Extended Data Fig. 1).

#### Fluorescence trace acquisition and binding state calling

We acquired fluorescence traces using custom FIJI and Matlab (R2022b) codes (using Localizer^74^ to read videos). Briefly, ten-frame average image stacks were made from the starting and ending 488-nm flashing (Methods) and DNA/MC spots were localized (SpotFinding.m) by fitting pixel intensities [*I*(*x*,*y*)] to a two-dimensional (2D) Gaussian distribution with rotation:

$$I\left(x,y\right)=A{e}^{-\left[\frac{{\left({x}_{\rm{rot}}-{x}_{0{\rm{rot}}}\right)}^{2}}{2{{\sigma }_{x}}^{2}}+\frac{{\left({y}_{\rm{rot}}-{y}_{0{\rm{rot}}}\right)}^{2}}{2{{\sigma }_{y}}^{2}}\right]}$$

where *A* is amplification, *σ*_*x*_ and *σ*_*y*_ describe width in the *x* and *y* directions and

$$\left[\begin{array}{cc}{x}_{\rm{rot}} & {y}_{\rm{rot}}\end{array}\right]=\left[\begin{array}{cc}x & y\end{array}\right]\bullet \left[\begin{array}{cc}\cos \varphi & \sin \varphi \\ -\sin \varphi & \cos \varphi \end{array}\right]$$

$$\left[\begin{array}{cc}{x}_{0\mathrm{rot}} & {y}_{0\mathrm{rot}}\end{array}\right]=\left[\begin{array}{cc}{x}_{0} & {y}_{0}\end{array}\right]\bullet \left[\begin{array}{cc}\cos \varphi & \sin \varphi \\ -\sin \varphi & \cos \varphi \end{array}\right]$$

where *φ* is the rotational angle and (*x*_0_, *y*_0_) are the center coordinates of 2D Gaussian distribution. Fitting was performed using lsqcurvefit in Matlab and the found spots were filtered on the basis of their brightness (*A*), width (*σ*_*x*_ and *σ*_*y*_) and *xy* asymmetry (*σ*_*x*_/*σ*_*y*_).

Positional drifting (DriftingCorrection.m) in videos (normally <2 pixels) is presumed to be linear. DNA/MC coordinates were projected to 6,000-frame TAMRA videos and fluorescence intensity was calculated (TraceGenerator.m) by averaging pixel brightness within 3 × 3 (linear DNA and MC) or 5 × 5 (ccDNA) squares centering each spot coordinate each frame and subtracting background brightness (average brightness of 7 × 7 hollow squares centering each coordinate each frame). The resulting matrix was *n* rows (DNA/MC spot number) × 6,001 columns (6,000 frames + numerical ID of the trace).

Binding state calling is semi-automatic (BindingCalling.m). For each trace, fluorescence intensity distribution was fitted to a multi-Gaussian distribution on the basis of which a cutoff was calculated. Any frame above the cutoff was noted as ‘bound’ (assigned ‘1’), while those below the cutoff were noted as ‘unbound’ (assigned ‘0’). Any interval < 3 frames were regarded as blinking and concatenated. We also manually discard traces with extremely fluctuating baselines (interference by adjacent molecules) or abnormally high signals (aggregates) by changing their numerical ID to ‘−1’. The resulting matrix was *n* rows × 6,001 columns binary with the last column being numerical ID or −1 (discarded trace).

For Cy5 nucleosome experiments, we only included traces with both AF488 and Cy5 signals at the beginning of the video.

For three-color FRET experiments, spot finding was completed in a 639-nm channel.

#### Residence time calculations and multicomponent exponential fitting (Fitting.m)

All residence times were calculated from the binary matrix for binding states. We discarded binding events that started in the middle but lasted until the end of a video (right-censored events) because they introduce high variations during fitting. Discarding these events leads to mild underestimation of F_sb_ and *τ*_sb_ for templates with long GCN4 *τ* such as WT ccDNA. For binding events lasting through an entire video, we included them as double-censored events, meaning that the actual residence time was no less than 600 s (video length), and we used cumulative probability function to fit them. We used the maximum-likelihood estimate function in Matlab, with the probability density function set to

$$\mathrm{pdf}\left(t\right)=\sum _{i}{{{\rm{F}}}_{i}{k}_{i}e}^{-{k}_{i}t},\quad\left(\sum _{i}{{\rm{F}}}_{i}=1\right)$$

where *i* refers to different components, F_*i*_ is the fraction of component *i* and *k*_*i*_ is the *k*_off_ for component *i*. Furthermore, the cumulative distribution function was set to

$$\mathrm{cdf}\left(t\right)=\sum _{i}{{\rm{F}}}_{i}{e}^{-{k}_{i}t},\quad\,\left(\sum _{i}{{\rm{F}}}_{i}=1\right)$$

‘Censoring’ was set to 1 for censored events and 0 for other binding events used for fitting. The final command was as follows:

mle(fitdata,‘pdf’,pdf,‘cdf’,cdf,‘Start’,starting_guesses,‘Censoring’,censoring_vector,‘LowerBound’,[0 0 0 0 0],‘UpperBound’,[1 1 1 1 1],‘Options’,statset(‘MaxIter’,100000,‘MaxFunEvals’,200000)).

We evaluated approaches ranging from single-exponential fitting to five-component fitting. For most ccDNA and MC samples, three-component fitting performed the best according to the Bayesian information criterion and the fitting results; thus, we used three-component fitting for all ccDNA and MC experiments (except for GCN4^∆AD^ data, where we used two-component fitting) and final values were averaged from different replicates with s.d. calculated as described above.

For linear DNA, two-component exponential fitting performed well. For WT linear DNA, the stable fraction was always dominant; hence, we presented *k*_off,sb_ values. For UAS^∆^ and other mutant linear DNA, there was always one fraction that was dominant; hence, we presented the dominant *k*_off_ value for mutant linear DNA. As linear DNA is PCR-generated and does not have biological replicates, we pooled together all binding events from different videos and used bootstrapping methods (200 resampling) to determine the 95% CI.

#### *k*_on_ calculations (ontimeFitting.m)

To avoid complications caused by photobleaching and secondary binding events, we only used the time duration before the first binding event (on-time) in each trace in the flow-in videos (described above) to calculate *k*_on_. On-times from all flow-in videos were pooled together and we applied a single-exponential fitting:

$$\mathrm{pdf}\left(t\right)=\left[\mathrm{GCN}4\right]{k}_{\mathrm{on}}{e}^{-\left[GCN4\right]{k}_{\mathrm{on}}t}$$

For *k*_on,sb_, we ignored any binding events shorter than *τ*_cutoff_ and applied the analysis described above.

Bootstrapping with 200 resampling was used to determine the 95% CI as described above.

#### MNase-seq analysis

We used minimap2 to map reads with the following command: minimap2 -r 5,5 --no-long-join -o outputfilename.paf referencesequence.fa fastqfilename.fastq.paf. Files were further processed using custom R code (version 4.2.2). First, all entries with gaps ≥ 10 bp (col 11 − col 10 ≥ 10) were discarded; second, redundant mappings were discarded (reads aligning to 6×LacO or 7×TetO could be assigned more than one position; thus, only one entry with the longest alignment length was kept and other entries for the same read were discarded); third, an alignment length filter was applied (skipped for Extended Data Fig. 1c); fourth, normalized coverage for each bp on MC sequence was calculated as follows:

$$\mathrm{Normalized}\,\mathrm{coverage}\,\mathrm{at}\,x\mathrm{th}\,\mathrm{bp}=\frac{\mathrm{no}.\,\mathrm{of}\,\mathrm{reads}\,\mathrm{covering}\,x\mathrm{th}\,\mathrm{bp}}{\sum _{\mathop{\mathrm{all}}\,{\mathrm{reads}}}\nolimits\mathrm{alignment}\,\mathrm{length}}\times 10,000$$

#### SMT analysis

In vivo GCN4 residence time was calculated using Sojourner and custom R codes as described previously^73,75^. MSD calculations followed other previous studies^44,76^ in the lab.

### Reporting summary

Further information on research design is available in the Nature Portfolio Reporting Summary linked to this article.

## Online content

Any methods, additional references, Nature Portfolio reporting summaries, source data, extended data, supplementary information, acknowledgements, peer review information; details of author contributions and competing interests; and statements of data and code availability are available at https://doi.org/10.1038/s41594-026-01864-x.

## Supplementary information

Supplementary Information Information on all DNA sequences used in this study.

Reporting Summary

Peer Review file

Supplementary Table 1 This table provides the exact values of residence time exponential fitting for each biological replicate of ccDNA and MC samples, average fitting results and s.d. values among these replicates, residence time fitting results and 95% CIs for linear DNA and nucleosome samples and fitting results with 95% CIs for all *k*_on_ measurements.

## Source data

Source Data Fig. 1 Uncropped field of view and example frames.

Source Data Figs. 2, 4 and 5 and Extended Data Figs. 2, 3 and 5–8 Source data.

Source Data Fig. 3 Uncropped gel. The third lane on the right is the UAS^∆^ 116N6 (no half-site) nucleosome.

Source Data Extended Data Fig. 1 Uncropped gel. The six lanes on the left side of marker lane represent a bacterial pBSK plasmid as a positive control for supercoiled DNA. The time points of the six lanes are 0 s, 30 s, 1 min, 5 min, 30 min and 60 min.

## Data Availability

All data involved in this study can be found in a public Johns Hopkins repository (https://doi.org/10.7281/T1LI0D6C). The FASTQ files for MNase-seq were deposited to the Sequence Read Archive under BioProject PRJNA1478324. Source data are provided with this paper.

## Code availability

All custom codes and example data involved in this study can be found in a public Johns Hopkins repository (https://doi.org/10.7281/T1LI0D6C).
