# Supplementary material for: Nucleosomes and IDRs suppress promiscuous GCN4 binding on minichromosomes
Source: Nat Struct Mol Biol. 2026 Aug 13;33(9):1332–40. doi: 10.1038/s41594-026-01864-x (PMC13561865; doi:10.1038/s41594-026-01864-x)
Supplement: Supplementary file 1 [file 41594_2026_1864_MOESM1_ESM.pdf]

nature structural & molecular biology

Supplementary information https://doi.org/10.1038/s41594-026-01864-x

Nucleosomes and IDRs suppress promiscuous GCN4 binding on minichromosomes

In the format provided by the authors and unedited

Sequence information

TATO7LO6-HIS3 (WT MC):

TRP1

ARS1

Poly dT

UAS

TATA box

HIS3 ORF

6x LacO

7x TetO

Underlined bolded degenerate motifs are scrambled for UAS$^{\Delta}$scr templates.

CTAGATTCCGATGCTGACTTGCTGGGTATTATATGTGTGCCCAATAGAAAGAGAACA
ATTGACCCGGTTATTGCAAGGAAAATTTCAAGTCTTGTAAAAGCATATAAAAATAGTT
CAGGCACTCCGAAATACTTGGTTGGCGTGTTTCGTAATCAACCTAAGGAGGATGTTT
TGGCTCTGGTCAATGATTACGGCATTGATATCGTCCAACTGCATGGAG**ATGAGTCGT**
GGCAAGAATACCAAGAGTTCCTCGGTTTGCCAGTTATTAAAAGACTCGTATTTCCAA
AAGACTGCAACATACTACTCAGTGCAGCTTCACAGAAACCTCATTCGTTTATTCCCTT
GTTTGATTCAGAAGCAGGTGGGACAGGTGAACTTTTGGATTGGAACTCGATTTCTG
ACTGGGTTGGAAGGCAAGAGAGCCCCGAAAGCTTACATTTTATGTTAGCTGGTGGA
CTGACGCCAGAAAATGTTGGTGATGCGCTTAGATTAAATGGCGTTATTGGTGTTGAT
GTAAGCGGAGGTGTGGAGACAAATGGTGTAAAAGACTCTAACAAAATAGCAAATTTC
GTCAAAAATGCTAAGAAATAGGTTATTACTGAGTAGTATTTATTTAAGTATTGTTTGTG
CACTTGCCTGCAGGCCTTTTGAAAAGCAAGCATAAAAGATCTAAACATAAAATCTGTA
AAATAACAAGATGTAAAGATAATGCTAAATCATTTGGCTTTTTGATTGATTGTACAGGA
AAATATACATCGCAGGGGGTTGACTTTTACCATTTCACCGCAATGGAATCAAACTTGT
TGAAGAGAATGTTCACAGGCGCATACGCTACA**ATGACCCGA**TTCTTGCTAGCCTTTT
CTCGGTCTTGCAAACAACCGCCCCGGATCCTTATGCCTCGGTAATGATTTTCATTTT
TTTTTTTCCACCTAGCGGA**TGACTC**TTTTTTTTTCTTAGCGATTGGCATTATCACATAA
TGAATTATACATTATATAAAGTAATGTGATTTCTTCGAAGAATATACTAAAAAATGAGCA
GGCAAGATAAACGAAGGCAAAG**ATGACAGAG**CAGAAAGCCCTAGTAAAGCGTATTA
CAAATGAAACCAAGATTCAGATTGCGATCTCTTTAAAGGGTGGTCCCCTAGCGGCA
CGAGCATCAGCCGTGCATAGAGCACTCGATCTTCCCAGAAAAAGAGGCAGAAGCA
GTAGCAGAACAGGCCACACAATCGCAAGTGATTAACGTCCACACAGGTATAGGGTT
TCTGGACCATATGATACATGCTCTGGCCAAGCATTCCGGCTGGTCGCTAATCGTTGA
GTGCATTGGTGACTTACACATAGACGACCATCACACCACTGAAGACTGCGGGATTG

CTCTCGGTCAAGCTTTTAAAGAGGCCCTAGGGGCCGTGCGTGGAGTAAAAAGGTTT
GGATCAGGATTTGCGCCTTTGGATGAGGCACTTTCCAGAGCGGTGGTAGATCTTTC
GAACAGGCCGTACGCAGTTGTCGAACTTGGTTTGCAAAGGGAGAAAGTAGGAGAT
CTCTCTTGCGAGATGATCCCGCATTTTCTTGAAAGCTTTGCAGAGGCTAGCAGAATT
ACCCTCCACGTTGATTGTCTGCGAGGCAAGAATGATCATCACCGTAGTGAGAGTGC
GTTCAAGGCTCTTGCGGTTGCCATAAGAGAAGCCACCTCGCCCAATGGTACCAACG
ATGTTCCCTCCACCAAAGGTGTTCTTATGTAGTGACACCGATTATTTAAAGCTGCAG
CATACGATATATATACATGTGTATATATGTATACCTATGAATGTCAGTAAGTATGTATACG
AACAGTATGATACTGAAG**ATGACAAGG**TAATGCATCATTCTAT**ACGTGTCAT**TCTGAA
CGGATCCGGCCGGATCGGGCAGCTTAGTATATAAATACACATGTACATACCTCTCTC
CGTATCCTCGTAATCATTTTCTTGTATTTATCGTCTTTTCGCTGTAAAAACTTTATCACA
CTTATCTCAAATACACTTATTAACCGCTTTTACTATTATCTTCTACGCTGACAGTAATAT
CAAACA**GTGACACAT**ATTAAACACAGTGGTTTCTTTGCATAAACACCATCAGCCTCA
AGTCGTCAAGTAAAGATTTCGTGTTCATGCAGATAGATAACAATCTATATGTTGATAAT
TAGCG**TTGCCTCAT**CAATGCGAGATCCGTTTAACCGGACCCTAGTGCACTTACCCC
ACGTTCGGTCCACTGTGTGCCGAACTAGCAATTGTGAGCGGATAACAATTACTAGCA
ATTGTGAGCGGATAACAATTACTAGCAATTGTGAGCGGATAACAATTACTAGCAATTG
TGAGCGGATAACAATTACTAGCAATTGTGAGCGGATAACAATTACTAGCAATTGTGAG
CGGATAACAATACTAGTTCCTTCACTATTTTAACATGTGGAATTCGTAA**TCATGTCAT**A
GCTGTTTCCTGTGTGAACACTGTGTGCCGAACATGCTCCTTCACTATTTTAACATGT
GGAATTCCGGGGGTCGAGATCCGTATCACGAGGCCCTTTCGTCTTCACTCGAGTTT
ACCACTCCCTATCAGTGATAGAGAAAAGTGAAAGTCGAGTTTACCACTCCCTATCAG
TGATAGAGAAAAGTGAAAGTCGAGTTTACCACTCCCTATCAGTGATAGAGAAAAGTG
AAAGTCGAGTTTACCACTCCCTATCAGTGATAGAGAAAAGTGAAAGTCGAGTTTACC
ACTCCCTATCAGTGATAGAGAAAAGTGAAAGTCGAGTTTACCACTCCCTATCAGTGA
TAGAGAAAAGTGAAAGTCGAGTTTACCACTCCCTATCAGTGATAGAGAAAAGTGAAA
GTCGGGCTCGGTTCCCCCGGAATTCGGTCGAAAAAAGAAAAGGAGAGGGCCAAGA
GGGAGGGCATTGGTGACTATTGAGCACGTGAGTATACGTGATTAAGCACACAAAGG
CAGCTTGGAGTATGTCTGTTATTAATTTCACAGGTAGTTCTGGTCCATTGGTGAAAGT
TTGCGGCTTGCAGAGCACAGAGGCCGCAGAATGTGCT

55-bp WT linear DNA, ordered from IDT:

Forward: AF488-
CATTTTTTTTTTTCCACCTAGCGGATGACTCTTTTTTTTTCTTAGCGATTGGCAT

Reverse: BiotinTEG-
ATGCCAATCGCTAAGAAAAAAAAAGAGTCATCCGCTAGGTGGAAAAAAAAAAATG

49-bp UAS$^{\Delta}$ linear DNA, IDT:

Forward: AF488-
CATTTTTTTTTTTCCACCTAGCGGATTTTTTTTTCTTAGCGATTGGCAT

Reverse: BiotinTEG-
ATGCCAATCGCTAAGAAAAAAAAATCCGCTAGGTGGAAAAAAAAAAATG

53-bp UAS^{half} linear DNA, IDT:

Forward: AF488-
CATTTTTTTTTTTCCACCTAGCGGATGACTTTTTTTTTCTTAGCGATTGGCAT

Reverse: BiotinTEG-
ATGCCAATCGCTAAGAAAAAAAAAGTCATCCGCTAGGTGGAAAAAAAAAAATG

For other linear DNA fragments, PCR were carried out on corresponding minichromosome templates using the same **forward primer (IDT):**

AF488-CGGATCCTTATGCCTCGGTAATG

and various **reverse primers (IDT)** listed below.

For 156-bp WT DNA and 150-bp UAS$^{\Delta}$ DNA:

BiotinTEG-GCCTGCTCATTTTTTAGTATATTCTTCGAAGAAATCAC

For 286-bp WT DNA, 280-bp UAS$^{\Delta}$ DNA, and 280-bp UAS$^{\Delta}$scr DNA:

BiotinTEG-TCTATGCACGGCTGATGCTC

For 587-bp WT DNA:

BiotinTEG-CGCTCTGGAAAGTGCCTCATC

For 3-color FRET experiments, the following oligos were ordered from IDT:

UAS forward:

CATTTTTTTTTTTCCACCTAGCGGATGACTCTTTTTT/iCy5N/TTCTTAGCGATTGGCAT

UAS reverse:

Phos-
CTAGATGCCAATCGCTAAGAAAAAAAAAGAGTCATCCGCTAGGTGGAAAAAAAAAAA
TG

Half-site forward:

Phos-
CTAGTGAGCAGGCAAGATAAACGAAGGAAAAGATGACAGAGCAGAAAGCCCTAGTA
AAGCGTA

Half-site reverse:

BiotinTEG-
TACGCTTTACTAGGGCTTTCTGCTCTGTCATCTTT/iCy7N/CCTTCGTTTATCTTGCCT
GCTCA

The **final ligated dsDNA product** is:

CATTTTTTTTTTTCCACCTAGCGGATGACTCTTTTTT/iCy5N/TTCTTAGCGATTGGCAT
CTAGTGAGCAGGCAAGATAAACGAAGGAAAAGATGACAGAGCAGAAAGCCCTAGTA
AAGCGTA-BiotinTEG

For long-linker nucleosomes, DNA fragments are cloned from **pCR2.1-HIS3-601halfsite** (or without half-site for no-half-site nucleosomes):

UAS

HIS3 promoter

Widom 601

ATGAC (half-site replacing the original CGGTT sequence)

Forward primer:

AF488-CGGATCCTTATGCCTCGGTAATG

Reverse primer:

BiotinTEG-ATCGATCTGGAGATACCCGGTG

AGCGCCCAATACGCAAACCGCCTCTCCCCGCGCGTTGGCCGATTCATTAATGCAGC
TGGCACGACAGGTTTCCCGACTGGAAAGCGGGCAGTGAGCGCAACGCAATTAATG
TGAGTTAGCTCACTCATTAGGCACCCCAGGCTTTACACTTTATGCTTCCGGCTCGTA
TGTTGTGTGGAATTGTGAGCGGATAACAATTTCACACAGGAAACAGCTATGACCATG
ATTACGCCAAGCTTGGTACCGAGCTCGGATCCACTAGTAACGGCCGCCAGTGTGCT
GGAATTCGCCCTTGCCTGCAGCGGATCCTTATGCCTCGGTAATGATTTTCATTTTTTT
TTTTCCACCTAGCGGA**TGACTC**TTTTTTTTTCTTAGCGATTGGCATTATCACATAATGA
ATTATACATTATATAAAGTAATGTGAACAGGATGTATATATCTTACACGTGCCTAGAGAC
TAGTAAGTAATCCTATTGG**ATGAC**AAAACGCGGTAGACAGCGCGTACGTGCGTTTAA
GCGGTGCTAGAGCTTGCTACGACCAATTAAGCGGCCTTAGCACCGGGTATCTCCAG
ATCGATGCAAGGGCGAATTCTGCAGATATCCATCACACTGGCGGCCGCTCGAGCAT
GCATCTAGAGGGCCCAATTCGCCCTATAGTGAGTCGTATTACAATTCACTGGCCGTC
GTTTTACAACGTCGTGACTGGGAAAACCCTGGCGTTACCCAACTTAATCGCCTTGC
AGCACATCCCCCTTTCGCCAGCTGGCGTAATAGCGAAGAGGCCCGCACCGATCGC
CCTTCCCAACAGTTGCGCAGCCTGAATGGCGAATGGACGCGCCCTGTAGCGGCGC
ATTAAGCGCGGCGGGTGTGGTGGTTACGCGCAGCGTGACCGCTACACTTGCCAGC
GCCCTAGCGCCCGCTCCTTTCGCTTTCTTCCCTTCCTTTCTCGCCACGTTCGCCGG

CTTTCCCCGTCAAGCTCTAAATCGGGGGCTCCCTTTAGGGTTCCGATTTAGTGCTTT
ACGGCACCTCGACCCCAAAAAACTTGATTAGGGTGATGGTTCACGTAGTGGGCCAT
CGCCCTGATAGACGGTTTTTCGCCCTTTGACGTTGGAGTCCACGTTCTTTAATAGTG
GACTCTTGTTCCAAACTGGAACAACACTCAACCCTATCTCGGTCTATTCTTTTGATTT
ATAAGGGATTTTGCCGATTTCGGCCTATTGGTTAAAAAATGAGCTGATTTAACAAAAA
TTTAACGCGAATTTTAACAAAATTCAGGGCGCAAGGGCTGCTAAAGGAAGCGGAAC
ACGTAGAAAGCCAGTCCGCAGAAACGGTGCTGACCCCGGATGAATGTCAGCTACT
GGGCTATCTGGACAAGGGAAAACGCAAGCGCAAAGAGAAAGCAGGTAGCTTGCAG
TGGGCTTACATGGCGATAGCTAGACTGGGCGGTTTTATGGACAGCAAGCGAACCGG
AATTGCCAGCTGGGGCGCCCTCTGGTAAGGTTGGGAAGCCCTGCAAAGTAAACTG
GATGGCTTTCTTGCCGCCAAGGATCTGATGGCGCAGGGGATCAAGATCTGATCAAG
AGACAGGATGAGGATCGTTTCGCATGATTGAACAAGATGGATTGCACGCAGGTTCT
CCGGCCGCTTGGGTGGAGAGGCTATTCGGCTATGACTGGGCACAACAGACAATCG
GCTGCTCTGATGCCGCCGTGTTCCGGCTGTCAGCGCAGGGGCGCCCGGTTCTTTT
TGTCAAGACCGACCTGTCCGGTGCCCTGAATGAACTGCAGGACGAGGCAGCGCG
GCTATCGTGGCTGGCCACGACGGGCGTTCCTTGCGCAGCTGTGCTCGACGTTGTC
ACTGAAGCGGGAAGGGACTGGCTGCTATTGGGCGAAGTGCCGGGGCAGGATCTC
CTGTCATCCCACCTTGCTCCTGCCGAGAAAGTATCCATCATGGCTGATGCAATGCG
GCGGCTGCATACGCTTGATCCGGCTACCTGCCCATTCGACCACCAAGCGAAACATC
GCATCGAGCGAGCACGTACTCGGATGGAAGCCGGTCTTGTCGATCAGGATGATCT
GGACGAAGAGCATCAGGGGCTCGCGCCAGCCGAACTGTTCGCCAGGCTCAAGGC
GCGCATGCCCGACGGCGAGGATCTCGTCGTGACCCATGGCGATGCCTGCTTGCCG
AATATCATGGTGGAAAATGGCCGCTTTTCTGGATTCATCGACTGTGGCCGGCTGGG
TGTGGCGGACCGCTATCAGGACATAGCGTTGGCTACCCGTGATATTGCTGAAGAGC
TTGGCGGCGAATGGGCTGACCGCTTCCTCGTGCTTTACGGTATCGCCGCTCCCGA
TTCGCAGCGCATCGCCTTCTATCGCCTTCTTGACGAGTTCTTCTGAATTGAAAAAGG
AAGAGTATGAGTATTCAACATTTCCGTGTCGCCCTTATTCCCTTTTTTGCGGCATTTT
GCCTTCCTGTTTTTGCTCACCCAGAAACGCTGGTGAAAGTAAAAGATGCTGAAGAT
CAGTTGGGTGCACGAGTGGGTTACATCGAACTGGATCTCAACAGCGGTAAGATCCT
TGAGAGTTTTCGCCCCGAAGAACGTTTTCCAATGATGAGCACTTTTAAAGTTCTGCT
ATGTGGCGCGGTATTATCCCGTATTGACGCCGGGCAAGAGCAACTCGGTCGCCGC
ATACACTATTCTCAGAATGACTTGGTTGAGTACTCACCAGTCACAGAAAAGCATCTTA
CGGATGGCATGACAGTAAGAGAATTATGCAGTGCTGCCATAACCATGAGTGATAACA
CTGCGGCCAACTTACTTCTGACAACGATCGGAGGACCGAAGGAGCTAACCGCTTTT
TTGCACAACATGGGGGATCATGTAACTCGCCTTGATCGTTGGGAACCGGAGCTGAA
TGAAGCCATACCAAACGACGAGCGTGACACCACGATGCCTGTAGCAATGGCAACAA
CGTTGCGCAAACTATTAACTGGCGAACTACTTACTCTAGCTTCCCGGCAACAATTAAT
AGACTGGATGGAGGCGGATAAAGTTGCAGGACCACTTCTGCGCTCGGCCCTTCCG
GCTGGCTGGTTTATTGCTGATAAATCTGGAGCCGGTGAGCGTGGGTCTCGCGGTAT
CATTGCAGCACTGGGGCCAGATGGTAAGCCCTCCCGTATCGTAGTTATCTACACGA

CGGGGAGTCAGGCAACTATGGATGAACGAAATAGACAGATCGCTGAGATAGGTGCC
TCACTGATTAAGCATTGGTAACTGTCAGACCAAGTTTACTCATATATACTTTAGATTGA
TTTAAAACTTCATTTTTAATTTAAAAGGATCTAGGTGAAGATCCTTTTTGATAATCTCAT
GACCAAAATCCCTTAACGTGAGTTTTCGTTCCACTGAGCGTCAGACCCCGTAGAAA
AGATCAAAGGATCTTCTTGAGATCCTTTTTTTCTGCGCGTAATCTGCTGCTTGCAAA
CAAAAAAACCACCGCTACCAGCGGTGGTTTGTTTGCCGGATCAAGAGCTACCAACT
CTTTTTCCGAAGGTAACTGGCTTCAGCAGAGCGCAGATACCAAATACTGTTCTTCTA
GTGTAGCCGTAGTTAGGCCACCACTTCAAGAACTCTGTAGCACCGCCTACATACCT
CGCTCTGCTAATCCTGTTACCAGTGGCTGCTGCCAGTGGCGATAAGTCGTGTCTTA
CCGGGTTGGACTCAAGACGATAGTTACCGGATAAGGCGCAGCGGTCGGGCTGAAC
GGGGGGTTCGTGCACACAGCCCAGCTTGGAGCGAACGACCTACACCGAACTGAG
ATACCTACAGCGTGAGCTATGAGAAAGCGCCACGCTTCCCGAAGGGAGAAAGGCG
GACAGGTATCCGGTAAGCGGCAGGGTCGGAACAGGAGAGCGCACGAGGGAGCTT
CCAGGGGGAAACGCCTGGTATCTTTATAGTCCTGTCGGGTTTCGCCACCTCTGACT
TGAGCGTCGATTTTTGTGATGCTCGTCAGGGGGGCGGAGCCTATGGAAAAACGCC
AGCAACGCGGCCTTTTTACGGTTCCTGGCCTTTTGCTGGCCTTTTGCTCACATGTT
CTTTCCTGCGTTATCCCCTGATTCTGTGGATAACCGTATTACCGCCTTTGAGTGAGC
TGATACCGCTCGCCGCAGCCGAACGACCGAGCGCAGCGAGTCAGTGAGCGAGGA
AGCGGAAG

For protein purification, GCN4 is expressed from **pET-6His-SUMO-3G-GCN4**:

GCN4 ORF

6xHis

SUMO

3xGly

GCN4 AD (deleted for GCN4$^{\Delta AD}$)

For GCN4-Cy3 used in 3-color FRET experiments, 3xGly is deleted and LPETGG is added to the C-terminus.

CAAGGAGATGGCGCCCAACAGTCCCCCGGCCACGGGGCCTGCCACCATACCCAC
GCCGAAACAAGCGCTCATGAGCCCGAAGTGGCGAGCCCGATCTTCCCCATCGGTG
ATGTCGGCGATATAGGCGCCAGCAACCGCACCTGTGGCGCCGGTGATGCCGGCCA
CGATGCGTCCGGCGTAGAGGATCGAGATCTCGATCCCGCGAAATTAATACGACTCA
CTATAGGGGAATTGTGAGCGGATAACAATTCCCCTCTAGAAATAATTTTGTTTAACTTT
AAGAAGGAGATATACATATGGGCAGCAGCCATCATCATCATCATCACGGCAGCGGC
CTGGTGCCGCGCGGCAGCGCTAGCATGTCGGACTCAGAAGTCAATCAAGAAGCTA
AGCCAGAGGTCAAGCCAGAAGTCAAGCCTGAGACTCACATCAATTTAAAGGTGTCC
GATGGATCTTCAGAGATCTTCTTCAAGATCAAAAAGACCACTCCTTTAAGAAGGCTG

ATGGAAGCGTTCGCTAAAAGACAGGGTAAGGAAATGGACTCCTTAAGATTCTTGTAC
GACGGTATTAGAATTCAAGCTGATCAGACCCCTGAAGATTTGGACATGGAGGATAAC
GATATTATTGAGGCTCACAGAGAACAGATTGGTGGTGGAGGAGGGTCCGAATATCA
GCCAAGTTTATTTGCTTTAAATCCAATGGGTTTCTCACCATTGGATGGTTCTAAATCA
ACCAACGAAAATGTATCTGCTTCCACTTCTACTGCCAAACCAATGGTTGGCCAATTG
ATTTTTGATAAATTCATCAAGACTGAAGAGGATCCAATTATCAAACAGGATACCCCTT
CGAACCTTGATTTTGATTTTGCTCTTCCACAAACGGCAACTGCACCTGATGCCAAGA
CCGTTTTGCCAATTCCGGAGCTAGATGACGCTGTAGTGGAATCTTTCTTTTCGTCAA
GCACTGATTCAACTCCAATGTTTGAGTATGAAAACCTAGAAGACAACTCTAAAGAAT
GGACATCCTTGTTTGACAATGACATTCCAGTTACCACTGACGATGTTTCATTGGCTG
ATAAGGCAATTGAATCCACTGAAGAAGTTTCTCTGGTACCATCCAATCTGGAAGTCT
CGACAACTTCATTCTTACCCACTCCTGTTCTAGAAGATGCTAAACTGACTCAAACAA
GAAAGGTTAAGAAACCAAATTCAGTCGTTAAGAAGTCACATCATGTTGGAAAGGATG
ACGAATCGAGACTGGATCATCTAGGTGTTGTTGCTTACAACCGCAAACAGCGTTCG
ATTCCACTTTCTCCAATTGTGCCCGAATCCAGTGATCCTGCTGCTCTAAAACGTGCT
AGAAACACTGAAGCCGCCAGGCGTTCTCGTGCGAGAAAGTTGCAAAGAATGAAAC
AACTTGAAGACAAGGTTGAAGAATTGCTTTCGAAAAATTATCACTTGGAAAATGAGG
TTGCCAGATTAAAGAAATTAGTTGGCGAACGCTGAAGACAAGCTTAGGTATTTATTC
GGCGCAAAGTGCGTCGGGTGATGCTGCCAACTTAGTCGAGCACCACCACCACCAC
CACTGAGATCCGGCTGCTAACAAAGCCCGAAAGGAAGCTGAGTTGGCTGCTGCCA
CCGCTGAGCAATAACTAGCATAACCCCTTGGGGCCTCTAAACGGGTCTTGAGGGGT
TTTTTGCTGAAAGGAGGAACTATATCCGGATTGGCGAATGGGACGCGCCCTGTAGC
GGCGCATTAAGCGCGGCGGGTGTGGTGGTTACGCGCAGCGTGACCGCTACACTTG
CCAGCGCCCTAGCGCCCGCTCCTTTCGCTTTCTTCCCTTCCTTTCTCGCCACGTTC
GCCGGCTTTCCCCGTCAAGCTCTAAATCGGGGGCTCCCTTTAGGGTTCCGATTTAG
TGCTTTACGGCACCTCGACCCCAAAAAACTTGATTAGGGTGATGGTTCACGTAGTG
GGCCATCGCCCTGATAGACGGTTTTTCGCCCTTTGACGTTGGAGTCCACGTTCTTT
AATAGTGGACTCTTGTTCCAAACTGGAACAACACTCAACCCTATCTCGGTCTATTCTT
TTGATTTATAAGGGATTTTGCCGATTTCGGCCTATTGGTTAAAAAATGAGCTGATTTAA
CAAAAATTTAACGCGAATTTTAACAAAATATTAACGCTTACAATTTAGGTGGCACTTTT
CGGGGAAATGTGCGCGGAACCCCTATTTGTTTATTTTTCTAAATACATTCAAATATGTA
TCCGCTCATGAATTAATTCTTAGAAAAACTCATCGAGCATCAAATGAAACTGCAATTT
ATTCATATCAGGATTATCAATACCATATTTTTGAAAAAGCCGTTTCTGTAATGAAGGAG
AAAACTCACCGAGGCAGTTCCATAGGATGGCAAGATCCTGGTATCGGTCTGCGATT
CCGACTCGTCCAACATCAATACAACCTATTAATTTCCCCTCGTCAAAAATAAGGTTAT
CAAGTGAGAAATCACCATGAGTGACGACTGAATCCGGTGAGAATGGCAAAAGTTTA
TGCATTTCTTTCCAGACTTGTTCAACAGGCCAGCCATTACGCTCGTCATCAAAATCA
CTCGCATCAACCAAACCGTTATTCATTCGTGATTGCGCCTGAGCGAGACGAAATACG
CGATCGCTGTTAAAAGGACAATTACAAACAGGAATCGAATGCAACCGGCGCAGGAA
CACTGCCAGCGCATCAACAATATTTTCACCTGAATCAGGATATTCTTCTAATACCTGG

AATGCTGTTTTCCCGGGGATCGCAGTGGTGAGTAACCATGCATCATCAGGAGTACG
GATAAAATGCTTGATGGTCGGAAGAGGCATAAATTCCGTCAGCCAGTTTAGTCTGAC
CATCTCATCTGTAACATCATTGGCAACGCTACCTTTGCCATGTTTCAGAAACAACTCT
GGCGCATCGGGCTTCCCATACAATCGATAGATTGTCGCACCTGATTGCCCGACATTA
TCGCGAGCCCATTTATACCCATATAAATCAGCATCCATGTTGGAATTTAATCGCGGCC
TAGAGCAAGACGTTTCCCGTTGAATATGGCTCATAACACCCCTTGTATTACTGTTTAT
GTAAGCAGACAGTTTTATTGTTCATGACCAAAATCCCTTAACGTGAGTTTTCGTTCCA
CTGAGCGTCAGACCCCGTAGAAAAGATCAAAGGATCTTCTTGAGATCCTTTTTTTCT
GCGCGTAATCTGCTGCTTGCAAACAAAAAAACCACCGCTACCAGCGGTGGTTTGTT
TGCCGGATCAAGAGCTACCAACTCTTTTTCCGAAGGTAACTGGCTTCAGCAGAGCG
CAGATACCAAATACTGTCCTTCTAGTGTAGCCGTAGTTAGGCCACCACTTCAAGAAC
TCTGTAGCACCGCCTACATACCTCGCTCTGCTAATCCTGTTACCAGTGGCTGCTGCC
AGTGGCGATAAGTCGTGTCTTACCGGGTTGGACTCAAGACGATAGTTACCGGATAA
GGCGCAGCGGTCGGGCTGAACGGGGGGTTCGTGCACACAGCCCAGCTTGGAGC
GAACGACCTACACCGAACTGAGATACCTACAGCGTGAGCTATGAGAAAGCGCCACG
CTTCCCGAAGGGAGAAAGGCGGACAGGTATCCGGTAAGCGGCAGGGTCGGAACA
GGAGAGCGCACGAGGGAGCTTCCAGGGGGAAACGCCTGGTATCTTTATAGTCCTG
TCGGGTTTCGCCACCTCTGACTTGAGCGTCGATTTTTGTGATGCTCGTCAGGGGG
GCGGAGCCTATGGAAAAACGCCAGCAACGCGGCCTTTTTACGGTTCCTGGCCTTTT
GCTGGCCTTTTGCTCACATGTTCTTTCCTGCGTTATCCCCTGATTCTGTGGATAACC
GTATTACCGCCTTTGAGTGAGCTGATACCGCTCGCCGCAGCCGAACGACCGAGCG
CAGCGAGTCAGTGAGCGAGGAAGCGGAAGAGCGCCTGATGCGGTATTTTCTCCTT
ACGCATCTGTGCGGTATTTCACACCGCAATGGTGCACTCTCAGTACAATCTGCTCTG
ATGCCGCATAGTTAAGCCAGTATACACTCCGCTATCGCTACGTGACTGGGTCATGGC
TGCGCCCCGACACCCGCCAACACCCGCTGACGCGCCCTGACGGGCTTGTCTGCT
CCCGGCATCCGCTTACAGACAAGCTGTGACCGTCTCCGGGAGCTGCATGTGTCAG
AGGTTTTCACCGTCATCACCGAAACGCGCGAGGCAGCTGCGGTAAAGCTCATCAG
CGTGGTCGTGAAGCGATTCACAGATGTCTGCCTGTTCATCCGCGTCCAGCTCGTTG
AGTTTCTCCAGAAGCGTTAATGTCTGGCTTCTGATAAAGCGGGCCATGTTAAGGGC
GGTTTTTTCCTGTTTGGTCACTGATGCCTCCGTGTAAGGGGGATTTCTGTTCATGGG
GGTAATGATACCGATGAAACGAGAGAGGATGCTCACGATACGGGTTACTGATGATGA
ACATGCCCGGTTACTGGAACGTTGTGAGGGTAAACAACTGGCGGTATGGATGCGG
CGGGACCAGAGAAAAATCACTCAGGGTCAATGCCAGCGCTTCGTTAATACAGATGT
AGGTGTTCCACAGGGTAGCCAGCAGCATCCTGCGATGCAGATCCGGAACATAATGG
TGCAGGGCGCTGACTTCCGCGTTTCCAGACTTTACGAAACACGGAAACCGAAGAC
CATTCATGTTGTTGCTCAGGTCGCAGACGTTTTGCAGCAGCAGTCGCTTCACGTTC
GCTCGCGTATCGGTGATTCATTCTGCTAACCAGTAAGGCAACCCCGCCAGCCTAGC
CGGGTCCTCAACGACAGGAGCACGATCATGCGCACCCGTGGGGCCGCCATGCCG
GCGATAATGGCCTGCTTCTCGCCGAAACGTTTGGTGGCGGGACCAGTGACGAAGG
CTTGAGCGAGGGCGTGCAAGATTCCGAATACCGCAAGCGACAGGCCGATCATCGT

CGCGCTCCAGCGAAAGCGGTCCTCGCCGAAAATGACCCAGAGCGCTGCCGGCAC
CTGTCCTACGAGTTGCATGATAAAGAAGACAGTCATAAGTGCGGCGACGATAGTCAT
GCCCCGCGCCCACCGGAAGGAGCTGACTGGGTTGAAGGCTCTCAAGGGCATCGG
TCGAGATCCCGGTGCCTAATGAGTGAGCTAACTTACATTAATTGCGTTGCGCTCACT
GCCCGCTTTCCAGTCGGGAAACCTGTCGTGCCAGCTGCATTAATGAATCGGCCAAC
GCGCGGGGAGAGGCGGTTTGCGTATTGGGCGCCAGGGTGGTTTTTCTTTTCACCA
GTGAGACGGGCAACAGCTGATTGCCCTTCACCGCCTGGCCCTGAGAGAGTTGCAG
CAAGCGGTCCACGCTGGTTTGCCCCAGCAGGCGAAAATCCTGTTTGATGGTGGTTA
ACGGCGGGATATAACATGAGCTGTCTTCGGTATCGTCGTATCCCACTACCGAGATAT
CCGCACCAACGCGCAGCCCGGACTCGGTAATGGCGCGCATTGCGCCCAGCGCCA
TCTGATCGTTGGCAACCAGCATCGCAGTGGGAACGATGCCCTCATTCAGCATTTGC
ATGGTTTGTTGAAAACCGGACATGGCACTCCAGTCGCCTTCCCGTTCCGCTATCGG
CTGAATTTGATTGCGAGTGAGATATTTATGCCAGCCAGCCAGACGCAGACGCGCCG
AGACAGAACTTAATGGGCCCGCTAACAGCGCGATTTGCTGGTGACCCAATGCGACC
AGATGCTCCACGCCCAGTCGCGTACCGTCTTCATGGGAGAAAATAATACTGTTGATG
GGTGTCTGGTCAGAGACATCAAGAAATAACGCCGGAACATTAGTGCAGGCAGCTTC
CACAGCAATGGCATCCTGGTCATCCAGCGGATAGTTAATGATCAGCCCACTGACGC
GTTGCGCGAGAAGATTGTGCACCGCCGCTTTACAGGCTTCGACGCCGCTTCGTTC
TACCATCGACACCACCACGCTGGCACCCAGTTGATCGGCGCGAGATTTAATCGCCG
CGACAATTTGCGACGGCGCGTGCAGGGCCAGACTGGAGGTGGCAACGCCAATCA
GCAACGACTGTTTGCCCGCCAGTTGTTGTGCCACGCGGTTGGGAATGTAATTCAGC
TCCGCCATCGCCGCTTCCACTTTTTCCCGCGTTTTCGCAGAAACGTGGCTGGCCTG
GTTCACCACGCGGGAAACGGTCTGATAAGAGACACCGGCATACTCTGCGACATCGT
ATAACGTTACTGGTTTCACATTCACCACCCTGAATTGACTCTCTTCCGGGCGCTATC
ATGCCATACCGCGAAAGGTTTTGCGCCATTCGATGGTGTCCGGGATCTCGACGCTC
TCCCTTATGCGACTCCTGCATTAGGAAGCAGCCCAGTAGTAGGTTGAGGCCGTTGA
GCACCGCCGCCGCAAGGAATGGTGCATG

For VP16^{AD} and GAL4^{AD} substitution, GCN4^{AD} above is replaced by:

VP16$^{413-490}$:

GCCCCCCCGACCGATGTCAGCCTGGGGGACGAGCTCCACTTAGACGGCGAGGAC
GTGGCGATGGCGCATGCCGACGCGCTAGACGATTTCGATCTGGACATGTTGGGGG
ACGGGGATTCCCCGGGTCCGGGATTTACCCCCCACGACTCCGCCCCCTACGGCGC
TCTGGATATGGCCGACTTCGAGTTTGAGCAGATGTTTACCGATGCCCTTGGAATTGA
CGAGTACGGTGGG

GAL4$^{763-881}$:

GCTTTGTTTGGTGGCGCCAATTTTAATCAAAGTGGGAATATTGCTGATAGCTCATTGT
CCTTCACTTTCACTAACAGTAGCAACGGTCCGAACCTCATAACAACTCAAACAAATT
CTCAAGCGCTTTCACAACCAATTGCCTCCTCTAACGTTCATGATAACTTCATGAATAA

TGAAATCACGGCTAGTAAAATTGATGATGGTAATAATTCAAAACCACTGTCACCTGGT
TGGACGGACCAAACTGCGTATAACGCGTTTGGAATCACTACAGGGATGTTTAATACC
ACTACAATGGATGATGTATATAACTATCTATTCGATGATGAAGATACCCCACCAAACCC
AAAAAAAGAG

Adaptor protein is expressed from **pRSF-ADP73**:

ORF

TetR

GR6 linker

SBP

TEV cleavage site

Protein G

6xHis

GGGGAATTGTGAGCGGATAACAATTCCCCTGTAGAAATAATTTTGTTTAACTTTAATA
AGGAGATATACCATGCCAAAGAAGAAGCGTAAGGTCTCTAGATTAGATAAAAGTAAA
GTGATTAACAGCGCATTAGAGCTACTTAATGAGGTCGGAATCGAAGGTTTAACAACC
CGTAAACTCGCCCAGAAGCTAGGTGTAGAGCAGCCTACATTGTATTGGCATGTAAAA
AATAAGCGGGCTTTGCTCGACGCCTTAGCCATTGAGATGTTAGATAGGCACCATACT
CACTTTTGCCCTTTAGAAGGGGAAAGCTGGCAAGATTTTTTACGTAATAACGCTAAA
AGTTTTAGATGTGCTTTACTAAGTCATCGCGATGGAGCAAAAGTACATTTAGGTACAC
GGCCTACAGAAAAACAGTATGAAACTCTCGAAAATCAATTAGCCTTTTTATGCCAACA
AGGTTTTTCACTAGAGAATGCATTATATGCACTCAGCGCTGTGGGGCATTTTACTTTA
GGTTGCGTATTGGAAGATCAAGAGCATCAAGTCGCTAAAGAAGAAAGGGAAACACC
TACTACTGATAGTATGCCGCCATTATTACGACAAGCTATCGAATTATTTGATCACCAAG
GTGCAGAGCCAGCCTTCTTATTCGGCCTTGAATTGATCATATGCGGATTAGAAAAAC
AACTTAAATGTGAAAGTGGGTCTGGATCCGGTGGGGGTAATGCCGAGGACACCATG
CGCAGCAAGGAGCTGGCCGAGCAGGAGGCAGCGCGGCAGCGGCAGTTGGCAGC
TGAGGAGGAGCAGAGGCGCCGGGAAGCCGAGGAGCGGGTGCAGAGGAGCCTGG
CAGCGGAGGAGGAAGCCGCACGGCAGCGCAAGGTCGCACTGGAGGAAGTCGAG
CGGCTCAAGGCCAAGGTTGAGGAAGCGCGGCGCCTGCGAGAGCGAGCTGAGCA
GGAGTCTGCGAGGCAGCTGCAGCTGGCCCAGGAGGCTGCCCAGAAACGGCTGCA
GGCGGAGGAGAAGGCGCACGCCTTTGTGGTGCAGCAGCGAGAAGAGGAGCTGCA
GCAGACTCTTCAGCAAGAGCAGAACATGCTGGAGCGGCTGCGGAGCGAGGCAGA
GGCAGCGCGGCGAGCTGCTGAGGAGGCGGAGGAGGCCCGGGAGCAGGCAGAAC
GTGAGGCAGCGCAGTCTAGGAAGCAAGTGGAAGAGGCCGAGCGGCTGAAGCAGT
CGGCAGAGGAGCAGGCTCAGGCCCAGGCCCAGGCGCAGGCGGCTGCAGAGAAA
CTGCGCAAGGAAGCGGAGCAGGAGGCGGCGCGTCGGGCCCAGGCGGAGCAGGC

TGCGTTGAAACAGAAGCAGGCAGCCGACGCGGAGATGGAGAAGCACAAGAAGTTT
GCAGAGCAGACGCTACGGCAGAAGGCTCAGGTAGAGCAGGAGCTGACCACGCTG
AGGCTGCAGCTCGAGGAGACCGACCACCAGAAGAGCATCCTGGATGAGGAGCTG
CAGCGGCTAAAGGCTGAGGTAACAGAGGCAGCCCGGCAGCGTAGCCAGGTAGAG
GAGGAGCTCTTCTCTGTCCGCGTGCAGATGGAGGAGCTGGGCAAACTCAAGGCTC
GCATTGAAGCTGAAAACCGGGCACTCATCCTTCGTCTCATGACCGGGGGCGGTGG
AGGATCCGGCGGCGGTGGTCATCACATGGACGAGAAGACCACCGGGTGGCGGGG
CGGCCACGTTGTGGAGGGTCTCGCTGGCGAGCTGGAGCAGCTCAGGGCCCGCTT
GGAGCACCATCCCCAGGGGCAACGCGAGCCTATCGATGAAAATCTGTACTTCCAG
GGGATCGATGAGAACCTGTACTTTCAGGGGGGTAGTACCCCGGCCGTGACGACCT
ACAAGCTGGTGATCAACGGCAAGACCCTGAAGGGCGAGACCACAACCAAGGCCGT
GGACGCTGAAACCGCAGAGAAGGCCTTCAAGCAGTACGCCAACGACAATGGCGTG
GACGGAGTGTGGACCTACGACGATGCCACCAAGACCTTCACCGTGACCGAGGTTA
ACACCCCCGCAGTCACCACATACAAACTGGTGATCAACGGAAAAACGCTGAAGGG
CGAAACTACGACCAAGGCTGTGGACGCGGAGACGGCCGAAAAGGCGTTCAAACA
GTATGCCAACGATAACGGTGTAGACGGCGTCTGGACATATGACGACGCGACAAAGA
CTTTCACTGTCACTGAAGGCAGTGGACACCACCACCATCATCATTAGTAACCTAGGC
TGCTGCCACCGCTGAGCAATAACTAGCATAACCCCTTGGGGCCTCTAAACGGGTCT
TGAGGGGTTTTTTGCTGAAACCTCAGGCATTTGAGAAGCACACGGTCACACTGCTT
CCGGTAGTCAATAAACCGGTAAACCAGCAATAGACATAAGCGGCTATTTAACGACCC
TGCCCTGAACCGACGACAAGCTGACGACCGGGTCTCCGCAAGTGGCACTTTTCGG
GGAAATGTGCGCGGAACCCCTATTTGTTTATTTTTCTAAATACATTCAAATATGTATCC
GCTCATGAATTAATTCTTAGAAAAACTCATCGAGCATCAAATGAAACTGCAATTTATTC
ATATCAGGATTATCAATACCATATTTTTGAAAAAGCCGTTTCTGTAATGAAGGAGAAAA
CTCACCGAGGCAGTTCCATAGGATGGCAAGATCCTGGTATCGGTCTGCGATTCCGA
CTCGTCCAACATCAATACAACCTATTAATTTCCCCTCGTCAAAAATAAGGTTATCAAG
TGAGAAATCACCATGAGTGACGACTGAATCCGGTGAGAATGGCAAAAGTTTATGCAT
TTCTTTCCAGACTTGTTCAACAGGCCAGCCATTACGCTCGTCATCAAAATCACTCGC
ATCAACCAAACCGTTATTCATTCGTGATTGCGCCTGAGCGAGACGAAATACGCGGT
CGCTGTTAAAAGGACAATTACAAACAGGAATCGAATGCAACCGGCGCAGGAACACT
GCCAGCGCATCAACAATATTTTCACCTGAATCAGGATATTCTTCTAATACCTGGAATG
CTGTTTTCCCGGGGATCGCAGTGGTGAGTAACCATGCATCATCAGGAGTACGGATA
AAATGCTTGATGGTCGGAAGAGGCATAAATTCCGTCAGCCAGTTTAGTCTGACCATC
TCATCTGTAACATCATTGGCAACGCTACCTTTGCCATGTTTCAGAAACAACTCTGGC
GCATCGGGCTTCCCATACAATCGATAGATTGTCGCACCTGATTGCCCGACATTATCG
CGAGCCCATTTATACCCATATAAATCAGCATCCATGTTGGAATTTAATCGCGGCCTAG
AGCAAGACGTTTCCCGTTGAATATGGCTCATACTCTTCCTTTTTCAATATTATTGAAG
CATTTATCAGGGTTATTGTCTCATGAGCGGATACATATTTGAATGTATTTAGAAAAATA
AACAAATAGGCATGCAGCGCTCTTCCGCTTCCTCGCTCACTGACTCGCTACGCTCG
GTCGTTCGACTGCGGCGAGCGGTGTCAGCTCACTCAAAAGCGGTAATACGGTTATC

CACAGAATCAGGGGATAAAGCCGGAAAGAACATGTGAGCAAAAAGCAAAGCACCG
GAAGAAGCCAACGCCGCAGGCGTTTTTCCATAGGCTCCGCCCCCCTGACGAGCAT
CACAAAAATCGACGCTCAAGCCAGAGGTGGCGAAACCCGACAGGACTATAAAGATA
CCAGGCGTTTCCCCCTGGAAGCTCCCTCGTGCGCTCTCCTGTTCCGACCCTGCCG
CTTACCGGATACCTGTCCGCCTTTCTCCCTTCGGGAAGCGTGGCGCTTTCTCATAG
CTCACGCTGTTGGTATCTCAGTTCGGTGTAGGTCGTTCGCTCCAAGCTGGGCTGTG
TGCACGAACCCCCCGTTCAGCCCGACCGCTGCGCCTTATCCGGTAACTATCGTCTT
GAGTCCAACCCGGTAAGACACGACTTATCGCCACTGGCAGCAGCCATTGGTAACTG
ATTTAGAGGACTTTGTCTTGAAGTTATGCACCTGTTAAGGCTAAACTGAAAGAACAG
ATTTTGGTGAGTGCGGTCCTCCAACCCACTTACCTTGGTTCAAAGAGTTGGTAGCT
CAGCGAACCTTGAGAAAACCACCGTTGGTAGCGGTGGTTTTTCTTTATTTATGAGAT
GATGAATCAATCGGTCTATCAAGTCAACGAACAGCTATTCCGTTACTCTAGATTTCAG
TGCAATTTATCTCTTCAAATGTAGCACCTGAAGTCAGCCCCATACGATATAAGTTGTA
ATTCTCATGTTAGTCATGCCCCGCGCCCACCGGAAGGAGCTGACTGGGTTGAAGG
CTCTCAAGGGCATCGGTCGAGATCCCGGTGCCTAATGAGTGAGCTAACTTACATTAA
TTGCGTTGCGCTCACTGCCCGCTTTCCAGTCGGGAAACCTGTCGTGCCAGCTGCA
TTAATGAATCGGCCAACGCGCGGGGAGAGGCGGTTTGCGTATTGGGCGCCAGGGT
GGTTTTTCTTTTCACCAGTGAGACGGGCAACAGCTGATTGCCCTTCACCGCCTGGC
CCTGAGAGAGTTGCAGCAAGCGGTCCACGCTGGTTTGCCCCAGCAGGCGAAAATC
CTGTTTGATGGTGGTTAACGGCGGGATATAACATGAGCTGTCTTCGGTATCGTCGTA
TCCCACTACCGAGATGTCCGCACCAACGCGCAGCCCGGACTCGGTAATGGCGCGC
ATTGCGCCCAGCGCCATCTGATCGTTGGCAACCAGCATCGCAGTGGGAACGATGC
CCTCATTCAGCATTTGCATGGTTTGTTGAAAACCGGACATGGCACTCCAGTCGCCTT
CCCGTTCCGCTATCGGCTGAATTTGATTGCGAGTGAGATATTTATGCCAGCCAGCCA
GACGCAGACGCGCCGAGACAGAACTTAATGGGCCCGCTAACAGCGCGATTTGCTG
GTGACCCAATGCGACCAGATGCTCCACGCCCAGTCGCGTACCGTCTTCATGGGAG
AAAATAATACTGTTGATGGGTGTCTGGTCAGAGACATCAAGAAATAACGCCGGAACA
TTAGTGCAGGCAGCTTCCACAGCAATGGCATCCTGGTCATCCAGCGGATAGTTAAT
GATCAGCCCACTGACGCGTTGCGCGAGAAGATTGTGCACCGCCGCTTTACAGGCT
TCGACGCCGCTTCGTTCTACCATCGACACCACCACGCTGGCACCCAGTTGATCGG
CGCGAGATTTAATCGCCGCGACAATTTGCGACGGCGCGTGCAGGGCCAGACTGGA
GGTGGCAACGCCAATCAGCAACGACTGTTTGCCCGCCAGTTGTTGTGCCACGCGG
TTGGGAATGTAATTCAGCTCCGCCATCGCCGCTTCCACTTTTTCCCGCGTTTTCGCA
GAAACGTGGCTGGCCTGGTTCACCACGCGGGAAACGGTCTGATAAGAGACACCGG
CATACTCTGCGACATCGTATAACGTTACTGGTTTCACATTCACCACCCTGAATTGACT
CTCTTCCGGGCGCTATCATGCCATACCGCGAAAGGTTTTGCGCCATTCGATGGTGT
CCGGGATCTCGACGCTCTCCCTTATGCGACTCCTGCATTAGGAAATTAATACGACTC
ACTATA

SNAP-LacI is expressed from **pRSF-nolacO-SNAP26f-lacI**:

ORF

6xHis

SNAP26f

LacI

GGCCTGTAGAAATAATTTTGTTTAACTTTAATAAGGAGATATACCATGGGCAGCAGCC
ATCACCATCATCACCACGGGAGCATGGACAAAGACTGCGAAATGAAGCGCACCACC
CTGGATAGCCCTCTGGGCAAGCTGGAACTGTCTGGGTGCGAACAGGGCCTGCACC
GTATCATCTTCCTGGGCAAAGGAACATCTGCCGCCGACGCCGTGGAAGTGCCTGC
CCCAGCCGCCGTGCTGGGCGGACCAGAGCCACTGATGCAGGCCACCGCCTGGCT
CAACGCCTACTTTCACCAGCCTGAGGCCATCGAGGAGTTCCCTGTGCCAGCCCTG
CACCACCCAGTGTTCCAGCAGGAGAGCTTTACCCGCCAGGTGCTGTGGAAACTGC
TGAAAGTGGTGAAGTTCGGAGAGGTCATCAGCTACAGCCACCTGGCCGCCCTGGC
CGGCAATCCCGCCGCCACCGCCGCCGTGAAAACCGCCCTGAGCGGAAATCCCGT
GCCCATTCTGATCCCCTGCCACCGGGTGGTGCAGGGCGACCTGGACGTGGGGGG
CTACGAGGGCGGGCTCGCCGTGAAAGAGTGGCTGCTGGCCCACGAGGGCCACAG
ACTGGGCAAGCCTGGGCTGGGTGGAGGCGGTTCAGGCGGTGGCATGGTGAAATA
TGTAACGTTATACGATGTCGCAGAGTATGCCGGTGTCTCTTATCAGACCGTTTCCCG
CGTGGTGAACCAGGCCAGCCACGTTTCTGCGAAAACGCGGGAAAAAGTGGAAGC
GGCGATGGCGGAGCTGAATTACATTCCCAACCGCGTGGCACAACAACTGGCGGGC
AAACAGTCGTTGCTGATTGGCGTTGCCACCTCCAGTCTGGCCCTGCACGCGCCGT
CGCAAATTGTCGCGGCGATTAAATCTCGCGCCGATCAACTGGGTGCCAGCGTGGT
GGTGTCGATGGTAGAACGAAGCGGCGTCGAAGCCTGTAAAGCGGCGGTGCACAAT
CTTCTCGCGCAACGCGTCAGTGGGCTGATCATTAACTATCCGCTGGATGACCAGGA
TGCCATTGCTGTGGAAGCTGCCTGCACTAATGTTCCGGCGTTATTTCTTGATGTCTC
TGACCAGACACCCATCAACAGTATTATTTTCTCCCATGAAGACGGTACGCGACTGGG
CGTGGAGCATCTGGTCGCATTGGGTCACCAGCAAATCGCGCTGTTAGCGGGCCCA
TTAAGTTCTGTCTCGGCGCGTCTGCGTCTGGCTGGCTGGCATAAATATCTCACTCG
CAATCAAATTCAGCCGATAGCGGAACGGGAAGGCGACTGGAGTGCCATGTCCGGT
TTTCAACAAACCATGCAAATGCTGAATGAGGGCATCGTTCCCACTGCGATGCTGGT
TGCCAACGATCAGATGGCGCTGGGCGCAATGCGCGCCATTACCGAGTCCGGGCTG
CGCGTTGGTGCGGACATCTCGGTAGTGGGATACGACGATACCGAAGACAGCTCAT
GTTATATCCCGCCGTTAACCACCATCAAACAGGATTTTCGCCTGCTGGGGCAAACCA
GCGTGGACCGCTTGCTGCAACTCTCTCAGGGCCAGGCGGTGAAGGGCAATCAGC
TGTTGCCCGTCTCACTGGTGAAAAGAAAAACCACCCTGGCGCCCAATACGCAAACC
GCCTCTCCCCGCGCGTTGGCCGATTCATTAATGCAGCTGTAATAGCTCGAGTCTGG
TAAAGAAACCGCTGCTGCGAAATTTGAACGCCAGCACATGGACTCGTCTACTAGCG
CAGCTTAATTAACCTAGGCTGCTGCCACCGCTGAGCAATAACTAGCATAACCCCTTG
GGGCCTCTAAACGGGTCTTGAGGGGTTTTTTGCTGAAACCTCAGGCATTTGAGAAG

CACACGGTCACACTGCTTCCGGTAGTCAATAAACCGGTAAACCAGCAATAGACATAA
GCGGCTATTTAACGACCCTGCCCTGAACCGACGACAAGCTGACGACCGGGTCTCC
GCAAGTGGCACTTTTCGGGGAAATGTGCGCGGAACCCCTATTTGTTTATTTTTCTAA
ATACATTCAAATATGTATCCGCTCATGAATTAATTCTTAGAAAAACTCATCGAGCATCA
AATGAAACTGCAATTTATTCATATCAGGATTATCAATACCATATTTTTGAAAAAGCCGTT
TCTGTAATGAAGGAGAAAACTCACCGAGGCAGTTCCATAGGATGGCAAGATCCTGG
TATCGGTCTGCGATTCCGACTCGTCCAACATCAATACAACCTATTAATTTCCCCTCGT
CAAAAATAAGGTTATCAAGTGAGAAATCACCATGAGTGACGACTGAATCCGGTGAGA
ATGGCAAAAGTTTATGCATTTCTTTCCAGACTTGTTCAACAGGCCAGCCATTACGCT
CGTCATCAAAATCACTCGCATCAACCAAACCGTTATTCATTCGTGATTGCGCCTGAG
CGAGACGAAATACGCGGTCGCTGTTAAAAGGACAATTACAAACAGGAATCGAATGC
AACCGGCGCAGGAACACTGCCAGCGCATCAACAATATTTTCACCTGAATCAGGATAT
TCTTCTAATACCTGGAATGCTGTTTTCCCGGGGATCGCAGTGGTGAGTAACCATGCA
TCATCAGGAGTACGGATAAAATGCTTGATGGTCGGAAGAGGCATAAATTCCGTCAGC
CAGTTTAGTCTGACCATCTCATCTGTAACATCATTGGCAACGCTACCTTTGCCATGTT
TCAGAAACAACTCTGGCGCATCGGGCTTCCCATACAATCGATAGATTGTCGCACCT
GATTGCCCGACATTATCGCGAGCCCATTTATACCCATATAAATCAGCATCCATGTTGG
AATTTAATCGCGGCCTAGAGCAAGACGTTTCCCGTTGAATATGGCTCATACTCTTCC
TTTTTCAATATTATTGAAGCATTTATCAGGGTTATTGTCTCATGAGCGGATACATATTTG
AATGTATTTAGAAAAATAAACAAATAGGCATGCAGCGCTCTTCCGCTTCCTCGCTCA
CTGACTCGCTACGCTCGGTCGTTCGACTGCGGCGAGCGGTGTCAGCTCACTCAAA
AGCGGTAATACGGTTATCCACAGAATCAGGGGATAAAGCCGGAAAGAACATGTGAG
CAAAAAGCAAAGCACCGGAAGAAGCCAACGCCGCAGGCGTTTTTCCATAGGCTCC
GCCCCCCTGACGAGCATCACAAAAATCGACGCTCAAGCCAGAGGTGGCGAAACCC
GACAGGACTATAAAGATACCAGGCGTTTCCCCCTGGAAGCTCCCTCGTGCGCTCTC
CTGTTCCGACCCTGCCGCTTACCGGATACCTGTCCGCCTTTCTCCCTTCGGGAAGC
GTGGCGCTTTCTCATAGCTCACGCTGTTGGTATCTCAGTTCGGTGTAGGTCGTTCG
CTCCAAGCTGGGCTGTGTGCACGAACCCCCCGTTCAGCCCGACCGCTGCGCCTTA
TCCGGTAACTATCGTCTTGAGTCCAACCCGGTAAGACACGACTTATCGCCACTGGC
AGCAGCCATTGGTAACTGATTTAGAGGACTTTGTCTTGAAGTTATGCACCTGTTAAG
GCTAAACTGAAAGAACAGATTTTGGTGAGTGCGGTCCTCCAACCCACTTACCTTGG
TTCAAAGAGTTGGTAGCTCAGCGAACCTTGAGAAAACCACCGTTGGTAGCGGTGGT
TTTTCTTTATTTATGAGATGATGAATCAATCGGTCTATCAAGTCAACGAACAGCTATTC
CGTTACTCTAGATTTCAGTGCAATTTATCTCTTCAAATGTAGCACCTGAAGTCAGCCC
CATACGATATAAGTTGTAATTCTCATGTTAGTCATGCCCCGCGCCCACCGGAAGGAG
CTGACTGGGTTGAAGGCTCTCAAGGGCATCGGTCGAGATCCCGGTGCCTAATGAG
TGAGCTAACTTACATTAATTGCGTTGCGCGAAATTAATACGACTCACTATA

For live-cell SMT, GCN4 is expressed from **pRS-REV1p-BPSV40NLS-GCN4-Halo**:

REV1 promoter

ORF

SV40 NLS

… indicates inserted GCN4 construct with 6xGGGGS linker

HaloTag

CYC1 terminator

GAGCTCCGGTGTTGTTATCCGATACAACCGGATATTTTTCTTTTAATGAGTCTAAACC
GTGATAGCTTCAGGTTAATACAATCAAAAAAAGCTCAAATATTCTTTTAATGCCGCGT
TCACAGATTCCAATTGAATACAACTAGGTAGTTCATTATATGAAGCCTTTGCTACTATT
TTTCACTATAGTCTGCCTTCACCTTAATGCAGACATCCACATATTTTAATCACTTTAAA
ATAAAAAGGAAGATATATTAGAAGCTATGATCCAATCTGTAAGCCAGATTAAAATTCAC
GAACTCTTCTTTCATTTGAATTGAATGCTTTGAGTTGGGGTAGATTATCGCAAATTAC
TCATCACATTTATTGACTACGAACTTGCTGATGTCCTTTTTTTATTTATATTTTTCTTCA
GTGAAGCGATTTTTTTTTTACACAGACCAAGACGGAAAAAAGTAGCTAAGGAAGAAA
ACAAAATCATGAAAAAAATGTGAAGTGATCATGCACATCGCATCAACTTAAACATTGG
CTTAGAGATATATAGAGTTAGAGTTTACGGCAACCTTTAAGCACCAATACCTTTTGGC
ATAGTCTAAAGACCTGGTTCTTAATTTTAAACAAATTTAACTAAAGATTTCCCTATCAA
AGAAGTAACGAGTTGACAGATTTTCTCAAAATAAATCGATACTGCATTTCTAGGCATA
TCCAGCGTCTAGATGAAACGGACGGCAGACGGATCCGAATTCGAGTCCCCGAAAA
AAAAACGGAAAGTT…GCTAGCGCAGAAATCGGTACTGGCTTTCCATTCGACCCCCA
TTATGTGGAAGTCCTGGGCGAGCGCATGCACTACGTCGATGTTGGTCCGCGCGAT
GGCACCCCTGTGCTGTTCCTGCACGGTAACCCGACCTCCTCCTACGTGTGGCGCA
ACATCATCCCGCATGTTGCACCGACCCATCGCTGCATTGCTCCAGACCTGATCGGT
ATGGGCAAATCCGACAAACCAGACCTGGGTTATTTCTTCGACGACCACGTCCGCTT
CATGGATGCCTTCATCGAAGCCCTGGGTCTGGAAGAGGTCGTCCTGGTCATTCACG
ACTGGGGCTCCGCTCTGGGTTTCCACTGGGCCAAGCGCAATCCAGAGCGCGTCAA
AGGTATTGCATTTATGGAGTTCATCCGCCCTATCCCGACCTGGGACGAATGGCCAG
AATTTGCCCGCGAGACCTTCCAGGCCTTCCGCACCACCGACGTCGGCCGCAAGCT
GATCATCGATCAGAACGTTTTTATCGAGGGTACGCTGCCGATGGGTGTCGTCCGCC
CGCTGACTGAAGTCGAGATGGACCATTACCGCGAGCCGTTCCTGAATCCTGTTGAC
CGCGAGCCACTGTGGCGCTTCCCAAACGAGCTGCCAATCGCCGGTGAGCCAGCG
AACATCGTCGCGCTGGTCGAAGAATACATGGACTGGCTGCACCAGTCCCCTGTCC
CGAAGCTGCTGTTCTGGGGCACCCCAGGCGTTCTGATCCCACCGGCCGAAGCCG
CTCGCCTGGCCAAAAGCCTGCCTAACTGCAAGGCTGTGGACATCGGCCCGGGTCT
GAATCTGCTGCAAGAAGACAACCCGGACCTGATCGGCAGCGAGATCGCGCGCTGG
CTGTCGACGCTAGAGATTTCCGGTTAAGTCGACCTCGAGTCATGTAATTAGTTATGT
CACGCTTACATTCACGCCCTCCCCCCACATCCGCTCTAACCGAAAAGGAAGGAGTT
AGACAACCTGAAGTCTAGGTCCCTATTTATTTTTTTATAGTTATGTTAGTATTAAGAAC
GTTATTTATATTTCAAATTTTTCTTTTTTTTCTGTACAGACGCGTGTACGCATGTAACAT

TATACTGAAAACCTTGCTTGAGAAGGTTTTGGGACGCTCGAAGGCTTTAATTTGCGG
CCGGTACCCAATTCGCCCTATAGTGAGTCGTATTACGCGCGCTCACTGGCCGTCGT
TTTACAACGTCGTGACTGGGAAAACCCTGGCGTTACCCAACTTAATCGCCTTGCAG
CACATCCCCCTTTCGCCAGCTGGCGTAATAGCGAAGAGGCCCGCACCGATCGCCC
TTCCCAACAGTTGCGCAGCCTGAATGGCGAATGGCGCGACGCGCCCTGTAGCGGC
GCATTAAGCGCGGCGGGTGTGGTGGTTACGCGCAGCGTGACCGCTACACTTGCCA
GCGCCCTAGCGCCCGCTCCTTTCGCTTTCTTCCCTTCCTTTCTCGCCACGTTCGCC
GGCTTTCCCCGTCAAGCTCTAAATCGGGGGCTCCCTTTAGGGTTCCGATTTAGTGC
TTTACGGCACCTCGACCCCAAAAAACTTGATTAGGGTGATGGTTCACGTAGTGGGC
CATCGCCCTGATAGACGGTTTTTCGCCCTTTGACGTTGGAGTCCACGTTCTTTAATA
GTGGACTCTTGTTCCAAACTGGAACAACACTCAACCCTATCTCGGTCTATTCTTTTG
ATTTATAAGGGATTTTGCCGATTTCGGCCTATTGGTTAAAAAATGAGCTGATTTAACAA
AAATTTAACGCGAATTTTAACAAAATATTAACGTTTACAATTTCCTGATGCGGTATTTT
CTCCTTACGCATCTGTGCGGTATTTCACACCGCATAGGGTAATAACTGATATAATTAA
ATTGAAGCTCTAATTTGTGAGTTTAGTATACATGCATTTACTTATAATACAGTTTTTTAG
TTTTGCTGGCCGCATCTTCTCAAATATGCTTCCCAGCCTGCTTTTCTGTAACGTTCA
CCCTCTACCTTAGCATCCCTTCCCTTTGCAAATAGTCCTCTTCCAACAATAATAATGT
CAGATCCTGTAGAGACCACATCATCCACGGTTCTATACTGTTGACCCAATGCGTCTC
CCTTGTCATCTAAACCCACACCGGGTGTCATAATCAACCAATCGTAACCTTCATCTCT
TCCACCCATGTCTCTTTGAGCAATAAAGCCGATAACAAAATCTTTGTCGCTCTTCGC
AATGTCAACAGTACCCTTAGTATATTCTCCAGTAGATAGGGAGCCCTTGCATGACAAT
TCTGCTAACATCAAAAGGCCTCTAGGTTCCTTTGTTACTTCTTCTGCCGCCTGCTTC
AAACCGCTAACAATACCTGGGCCCACCACACCGTGTGCATTCGTAATGTCTGCCCA
TTCTGCTATTCTGTATACACCCGCAGAGTACTGCAATTTGACTGTATTACCAATGTCA
GCAAATTTTCTGTCTTCGAAGAGTAAAAAATTGTACTTGGCGGATAATGCCTTTAGCG
GCTTAACTGTGCCCTCCATGGAAAAATCAGTCAAGATATCCACATGTGTTTTTAGTAA
ACAAATTTTGGGACCTAATGCTTCAACTAACTCCAGTAATTCCTTGGTGGTACGAAC
ATCCAATGAAGCACACAAGTTTGTTTGCTTTTCGTGCATGATATTAAATAGCTTGGCA
GCAACAGGACTAGGATGAGTAGCAGCACGTTCCTTATATGTAGCTTTCGACATGATT
TATCTTCGTTTCCTGCAGGTTTTTGTTCTGTGCAGTTGGGTTAAGAATACTGGGCAA
TTTCATGTTTCTTCAACACTACATATGCGTATATATACCAATCTAAGTCTGTGCTCCTT
CCTTCGTTCTTCCTTCTGTTCGGAGATTACCGAATCAAAAAAATTTCAAGGAAACCG
AAATCAAAAAAAAGAATAAAAAAAAAATGATGAATTGAATTGAAAAGCTGTGGTATGG
TGCACTCTCAGTACAATCTGCTCTGATGCCGCATAGTTAAGCCAGCCCCGACACCC
GCCAACACCCGCTGACGCGCCCTGACGGGCTTGTCTGCTCCCGGCATCCGCTTAC
AGACAAGCTGTGACCGTCTCCGGGAGCTGCATGTGTCAGAGGTTTTCACCGTCAT
CACCGAAACGCGCGAGACGAAAGGGCCTCGTGATACGCCTATTTTTATAGGTTAAT
GTCATGATAATAATGGTTTCTTAGACGTCAGGTGGCACTTTTCGGGGAAATGTGCGC
GGAACCCCTATTTGTTTATTTTTCTAAATACATTCAAATATGTATCCGCTCATGAGACA
ATAACCCTGATAAATGCTTCAATAATATTGAAAAAGGAAGAGTATGAGTATTCAACATT

TCCGTGTCGCCCTTATTCCCTTTTTTGCGGCATTTTGCCTTCCTGTTTTTGCTCACC
CAGAAACGCTGGTGAAAGTAAAAGATGCTGAAGATCAGTTGGGTGCACGAGTGGG
TTACATCGAACTGGATCTCAACAGCGGTAAGATCCTTGAGAGTTTTCGCCCCGAAG
AACGTTTTCCAATGATGAGCACTTTTAAAGTTCTGCTATGTGGCGCGGTATTATCCCG
TATTGACGCCGGGCAAGAGCAACTCGGTCGCCGCATACACTATTCTCAGAATGACT
TGGTTGAGTACTCACCAGTCACAGAAAAGCATCTTACGGATGGCATGACAGTAAGA
GAATTATGCAGTGCTGCCATAACCATGAGTGATAACACTGCGGCCAACTTACTTCTG
ACAACGATCGGAGGACCGAAGGAGCTAACCGCTTTTTTGCACAACATGGGGGATCA
TGTAACTCGCCTTGATCGTTGGGAACCGGAGCTGAATGAAGCCATACCAAACGACG
AGCGTGACACCACGATGCCTGTAGCAATGGCAACAACGTTGCGCAAACTATTAACT
GGCGAACTACTTACTCTAGCTTCCCGGCAACAATTAATAGACTGGATGGAGGCGGA
TAAAGTTGCAGGACCACTTCTGCGCTCGGCCCTTCCGGCTGGCTGGTTTATTGCTG
ATAAATCTGGAGCCGGTGAGCGTGGGTCTCGCGGTATCATTGCAGCACTGGGGCC
AGATGGTAAGCCCTCCCGTATCGTAGTTATCTACACGACGGGGAGTCAGGCAACTAT
GGATGAACGAAATAGACAGATCGCTGAGATAGGTGCCTCACTGATTAAGCATTGGTA
ACTGTCAGACCAAGTTTACTCATATATACTTTAGATTGATTTAAAACTTCATTTTTAATT
TAAAAGGATCTAGGTGAAGATCCTTTTTGATAATCTCATGACCAAAATCCCTTAACGT
GAGTTTTCGTTCCACTGAGCGTCAGACCCCGTAGAAAAGATCAAAGGATCTTCTTG
AGATCCTTTTTTTCTGCGCGTAATCTGCTGCTTGCAAACAAAAAAACCACCGCTACC
AGCGGTGGTTTGTTTGCCGGATCAAGAGCTACCAACTCTTTTTCCGAAGGTAACTG
GCTTCAGCAGAGCGCAGATACCAAATACTGTCCTTCTAGTGTAGCCGTAGTTAGGC
CACCACTTCAAGAACTCTGTAGCACCGCCTACATACCTCGCTCTGCTAATCCTGTTA
CCAGTGGCTGCTGCCAGTGGCGATAAGTCGTGTCTTACCGGGTTGGACTCAAGAC
GATAGTTACCGGATAAGGCGCAGCGGTCGGGCTGAACGGGGGGTTCGTGCACACA
GCCCAGCTTGGAGCGAACGACCTACACCGAACTGAGATACCTACAGCGTGAGCTAT
GAGAAAGCGCCACGCTTCCCGAAGGGAGAAAGGCGGACAGGTATCCGGTAAGCG
GCAGGGTCGGAACAGGAGAGCGCACGAGGGAGCTTCCAGGGGGAAACGCCTGG
TATCTTTATAGTCCTGTCGGGTTTCGCCACCTCTGACTTGAGCGTCGATTTTTGTGAT
GCTCGTCAGGGGGGCGGAGCCTATGGAAAAACGCCAGCAACGCGGCCTTTTTACG
GTTCCTGGCCTTTTGCTGGCCTTTTGCTCACATGTTCTTTCCTGCGTTATCCCCTGA
TTCTGTGGATAACCGTATTACCGCCTTTGAGTGAGCTGATACCGCTCGCCGCAGCC
GAACGACCGAGCGCAGCGAGTCAGTGAGCGAGGAAGCGGAAGAGCGCCCAATAC
GCAAACCGCCTCTCCCCGCGCGTTGGCCGATTCATTAATGCAGCTGGCACGACAG
GTTTCCCGACTGGAAAGCGGGCAGTGAGCGCAACGCAATTAATGTGAGTTACCTCA
CTCATTAGGCACCCCAGGCTTTACACTTTATGCTTCCGGCTCCTATGTTGTGTGGAA
TTGTGAGCGGATAACAATTTCACACAGGAAACAGCTATGACCATGATTACGCCAAGC
GCGCAATTAACCCTCACTAAAGGGAACAAAAGCTG
