# Supplementary material for: Nucleosomes and IDRs suppress promiscuous GCN4 binding on minichromosomes
Source: Nat Struct Mol Biol. 2026 Aug 13;33(9):1332–40. doi: 10.1038/s41594-026-01864-x (PMC13561865; doi:10.1038/s41594-026-01864-x)
Supplement: Supplementary file 2 [file 41594_2026_1864_MOESM2_ESM.pdf]

Corresponding author(s): Carl Wu

Last updated by author(s): Jun 24, 2026

Reporting Summary

Nature Portfolio wishes to improve the reproducibility of the work that we publish. This form provides structure for consistency and transparency in reporting. For further information on Nature Portfolio policies, see our Editorial Policies and the Editorial Policy Checklist.

Statistics

For all statistical analyses, confirm that the following items are present in the figure legend, table legend, main text, or Methods section.

n/a	Confirmed	
☐	☒	The exact sample size (n) for each experimental group/condition, given as a discrete number and unit of measurement
☐	☒	A statement on whether measurements were taken from distinct samples or whether the same sample was measured repeatedly
☐	☒	The statistical test(s) used AND whether they are one- or two-sided<br>*Only common tests should be described solely by name; describe more complex techniques in the Methods section.*
☒	☐	A description of all covariates tested
☒	☐	A description of any assumptions or corrections, such as tests of normality and adjustment for multiple comparisons
☐	☒	A full description of the statistical parameters including central tendency (e.g. means) or other basic estimates (e.g. regression coefficient) AND variation (e.g. standard deviation) or associated estimates of uncertainty (e.g. confidence intervals)
☐	☒	For null hypothesis testing, the test statistic (e.g. F, t, r) with confidence intervals, effect sizes, degrees of freedom and P value noted<br>*Give P values as exact values whenever suitable.*
☒	☐	For Bayesian analysis, information on the choice of priors and Markov chain Monte Carlo settings
☒	☐	For hierarchical and complex designs, identification of the appropriate level for tests and full reporting of outcomes
☒	☐	Estimates of effect sizes (e.g. Cohen's d, Pearson's r), indicating how they were calculated

Our web collection on statistics for biologists contains articles on many of the points above.

Software and code

Policy information about availability of computer code

Data collection	TIRF videos were collected by Micro Manager gamma 2.0 (https://micro-manager.org/Download_Micro-Manager_Latest_Release). Live cell SMT videos were collected by HCImage (https://hcimage.com/download/). MNase-seq were collected by Plasmidsaurus.
Data analysis	TIRF videos were processed by custom FIJI (ImageJ) and Matlab (R2022b) scripts. The Matlab scripts include a published package 'Localizer' (Dedecker, et al, doi:10.1117/1.JBO.17.12.126008) for .tif file reading. Live cell SMT videos were processed by Diatrack (Vallotton and Olivier, doi:10.1017/S1431927612014328) and custom R codes (Ling, et al, doi:10.1038/s41556-024-01382-2). MNase-seq data were processed by minimap2 and custom R codes (R 4.2.2). Visualizations were completed by Matlab (R2022b).

For manuscripts utilizing custom algorithms or software that are central to the research but not yet described in published literature, software must be made available to editors and reviewers. We strongly encourage code deposition in a community repository (e.g. GitHub). See the Nature Portfolio guidelines for submitting code & software for further information.

Data

Policy information about availability of data

All manuscripts must include a data availability statement. This statement should provide the following information, where applicable:

- Accession codes, unique identifiers, or web links for publicly available datasets
- A description of any restrictions on data availability
- For clinical datasets or third party data, please ensure that the statement adheres to our policy

All data, raw videos, and custom codes within this manuscript and its supplementary information are available at https://doi.org/10.7281/T1LI0D6C. Fastq files for MNase-seq are also available at SRA under accession number PRJNA1478324. All third party data are publicly available.

Research involving human participants, their data, or biological material

Policy information about studies with human participants or human data. See also policy information about sex, gender (identity/presentation), and sexual orientation and race, ethnicity and racism.

Reporting on sex and gender	No human participant involved.
Reporting on race, ethnicity, or other socially relevant groupings	No human participant involved.
Population characteristics	No human participant involved.
Recruitment	No human participant involved.
Ethics oversight	No human participant involved.

Note that full information on the approval of the study protocol must also be provided in the manuscript.

Field-specific reporting

Please select the one below that is the best fit for your research. If you are not sure, read the appropriate sections before making your selection.

☒ Life sciences ☐ Behavioural & social sciences ☐ Ecological, evolutionary & environmental sciences

For a reference copy of the document with all sections, see nature.com/documents/nr-reporting-summary-flat.pdf

Life sciences study design

All studies must disclose on these points even when the disclosure is negative.

Sample size

For ccDNA and MC, total binding events (n) used for calculating residence time (Fig. 2, 3, 4h, 5, Extended Data Fig. 2, 3, 4b-4c, 4e, 5) are:
GCN4 (GCN4FL) on WT ccDNA, n=1021; UASΔ ccDNA, n=1468; UASΔscr ccDNA, n=1589; WT MC, n=2738; UASΔ MC, n=6131; UASΔscr MC, n=7499; PromoterΔ MC, n=5614; RSC-treated MC, n=4170.
GCN4ΔAD on WT ccDNA, n=443; UASΔ ccDNA, n=337; WT MC, n=561; UASΔ MC, n=857.
VP16AD-GCN4DBD on WT ccDNA, n=5527; UASΔ ccDNA, n=11920; WT MC, n=17039; UASΔ MC, n=15343.
GAL4AD-GCN4DBD on WT ccDNA, n=846; UASΔ ccDNA, n=1117; WT MC, n=1176; UASΔ MC, n=996.
For linear DNA fragments and reconstituted nucleosomes, there is no 'biological replicate' since they are synthesized, so we pool together all data from different experiments and the total number of binding events for residence time calculations (Fig. 2, 3, 5, Extended Data Fig. 2, 3, 5) are:
GCN4 (GCN4FL) on WT 55-bp, n=1213; WT 156-bp, n=1282; WT 286-bp, n=739; UASΔ 49-bp, n=373; UASΔ 150-bp, n=3098; UASΔ 280-bp, n=2333; UAShalf 53-bp, n=1452; UASΔscr 280-bp, n=5049; WT HIS3-601 (half-site) 275-bp, n=2160; WT-601 (half-site) nucleosome (122N6), n=2121; UASΔ-601 (half-site) 269-bp DNA, n=8542; UASΔ-601 (half-site) nucleosome (116N6), n=6414; UASΔ-601 (no half-site) 269-bp DNA, n=12632; UASΔ-601 (no half-site) nucleosome (116N6), n=2687.
GCN4ΔAD on WT 55-bp, n=646; UASΔ 49-bp, n=170.
When analyzing association rates (kon and kon,sb, Fig. 4, 5, Extended Data Fig. 4, 5), we only use the 'flow-in' videos in each imaging session. We pool together 'flow-in' traces from all experiments. Total numbers of 'flow-in' traces are:
GCN4 (GCN4FL) on WT 55-bp, n=232; WT 156-bp, n=279; WT 286-bp, n=203; WT 587-bp, n=294; WT-601 (half-site) nucleosome (122N6), n=266; WT ccDNA, n=396; WT MC, n=266; PromoterΔ MC, n=245; RSC-treated MC, n=169.
GCN4ΔAD on WT 55-bp, n=138; WT ccDNA, n=54; WT MC, n=90.
VP16AD-GCN4DBD on WT ccDNA, n=187; WT MC, n=283.
GAL4AD-GCN4DBD on WT ccDNA, n=129; WT MC, n=96.
For live-cell SMT (Fig. 5, Extended Data Fig. 8), we pool together all data and collect in total 868 trajectories for GCN4FL and 1776 trajectories for GCN4ΔAD.

Data exclusions

For TIRF data, traces with extremely fluctuating baselines (interference by adjacent molecules) or abnormally high signals (protein aggregates) are manually excluded. The exclusion criteria were pre-established in the field to address common technical issues (Feng, et al. doi:10.1038/

s41594-025-01643-0). For live cell SMT, trajectories with poor spot localization were excluded as described in previous study (Ling, et al. doi:10.1126/science.ads0960). For MNase-seq, reads were selected based on their length (125 bp-175 bp), which is pre-estblished in the field for selecting mono-nucleosome footprint specifically.

Replication

For ccDNA and MC, numbers of biological replicates (purified from independent yeast cultures) (Fig. 2, 3, 4h, 5, Extended Data Fig. 2, 3, 4b-4c, 4e, 5) are:
GCN4 (GCN4FL) on WT ccDNA, 4 reps; UASΔ ccDNA, 3 reps; UASΔscr ccDNA, 2 reps; WT MC, 3 reps; UASΔ MC, 3 reps; UASΔscr MC, 2 reps; PromoterΔ MC, 3 reps; RSC-treated MC, 3 reps.
GCN4ΔAD on WT ccDNA, 2 reps; UASΔ ccDNA, 2 reps; WT MC, 2 reps; UASΔ MC, 2 reps.
VP16AD-GCN4DBD on WT ccDNA, 2 reps; UASΔ ccDNA, 2 reps; WT MC, 3 reps; UASΔ MC, 2 reps.
GAL4AD-GCN4DBD on WT ccDNA, 2 reps; UASΔ ccDNA, 2 reps; WT MC, 2 reps; UASΔ MC, 2 reps.
For linear DNA fragments and reconstituted nucleosomes, there is no 'biological replicate' since they are synthesized, so we pool together all data from at least three imaging sessions (technical replicates) (Fig. 2, 3, 5, Extended Data Fig. 2, 3, 5).
For live-cell SMT (Fig. 5, Extended Data Fig. 8), we pool together all data from 4 (GCN4FL) or 3 (GCN4ΔAD) independent cell cultures and imaging sessions (biological replicates) and collect in total 868 trajectories for GCN4FL and 1776 trajectories for GCN4ΔAD.
For MNase-seq, averages of 2 biological replicates (independent yeast culture) are shown for each MC nucleosome map (Fig. 1, 3, 4, Extended Data Fig. 1).

Randomization

Randomization is not relevant to this study.

Blinding

Blinding is not relevant to this study.

Reporting for specific materials, systems and methods

We require information from authors about some types of materials, experimental systems and methods used in many studies. Here, indicate whether each material, system or method listed is relevant to your study. If you are not sure if a list item applies to your research, read the appropriate section before selecting a response.

Materials & experimental systems

n/a	Involved in the study
☒	☐ Antibodies
☒	☐ Eukaryotic cell lines
☒	☐ Palaeontology and archaeology
☒	☐ Animals and other organisms
☒	☐ Clinical data
☒	☐ Dual use research of concern
☒	☐ Plants

Methods

n/a	Involved in the study
☒	☐ ChIP-seq
☒	☐ Flow cytometry
☒	☐ MRI-based neuroimaging

Plants

Seed stocks

No plant used in this study.

Novel plant genotypes

No plant used in this study.

Authentication

No plant used in this study.
