# Supplementary material for: Nucleosomes and IDRs suppress promiscuous GCN4 binding on minichromosomes
Source: Nat Struct Mol Biol. 2026 Aug 13;33(9):1332–40. doi: 10.1038/s41594-026-01864-x (PMC13561865; doi:10.1038/s41594-026-01864-x)
Supplement: Supplementary file 3 [file 41594_2026_1864_MOESM3_ESM.pdf]

nature portfolio

Peer Review File

Nucleosomes and IDRs suppress promiscuous GCN4 binding on minichromosomes

Corresponding Author: Professor Carl Wu

Version 0:

Decision Letter:

17th Apr 2026

Dear Professor Wu,

Thank you again for submitting your manuscript "Nucleosome organization and TF-IDRs facilitate target search by suppressing TF promiscuity on chromatin". I am very sorry for the delay in responding, which resulted from the difficulty in timely obtaining referee reports, with originally appointed Reviewer #3 not returning their report despite our repeated requests. Nevertheless, we now have comments (below) from the 2 reviewers who evaluated your paper. These reviewers cover both the technical and conceptual expertise needed to provide a comprehensive review of the work. In light of their reports, we remain interested in your study and would like to see your response to the comments of the referees in the form of a revised manuscript.

You will see that while the Reviewers appreciate the methods and systems used, they still raise notable concerns that must be entirely addressed in a revised manuscript. In particular, Reviewer #2 notes that errors and statistics much be more comprehensively reported and taken into consideration for conclusions to be considered valid. Furthermore, Reviewer #2 requests that the possibility of hopping, instead of 1D sliding, is tested and, along similar lines, Reviewer #1 notes that currently there is limited direct evidence for a 1D scanning model. Reviewer #2 additionally asks that generalisability claims are removed or the data are expanded to another TF with similar behaviour and both reviewers ask for certain clarifications and toning down in relevant sections. Please be sure to respond to all raised points in a detailed point-by-point response when submitting the revised manuscript

Our standard revision period is 3-6 months. We appreciate that the requested revisions are extensive. We are committed to providing a fair and constructive peer-review process and encourage you to contact us if you would like to discuss the reviews or anticipate any delay with the resubmission.

We will be happy to consider your revision as long as nothing similar has been accepted for publication at NSMB or published elsewhere. Should your manuscript be substantially delayed without notifying us in advance and your article is eventually published, the received date would be that of the revised, not the original, version.

REPORTING AND DATA AVAILABILITY: Please provide updated files as follows with your revision. As you already know, we put great emphasis on ensuring that the methods and statistics reported in our papers are correct and accurate.

1. REPORTING SUMMARY: Please provide an updated file:

https://www.nature.com/documents/nr-reporting-summary.pdf

This form is a dynamic ‘smart pdf’ and must be downloaded and completed in Adobe Reader.

2. REPORTING OF STRUCTURAL DATA: If there are additional or modified structures in the revision, please submit the corresponding PDB validation reports with your other files, and use the figshare integration system to provide access to maps, half-maps, and models.

Manuscripts reporting new structures should contain a table summarizing structural and refinement statistics. Please include these tables for new cryo-EM or -ET (https://www.nature.com/documents/nr-tables-cryo-em.doc and modifying this file for ET), NMR (https://www.nature.com/documents/nr-tables-nmr.doc) and X-ray crystallography

(https://www.nature.com/documents/nr-tables-xray.doc). To facilitate assessment of the quality of the structural data, a stereo image of a portion of the electron density map (for crystallography papers) or of the superimposed lowest energy structures (>10; for NMR papers) should be provided with the submitted manuscript. If the reported structure represents a novel overall fold, a stereo image of the entire structure (as a backbone trace) should also be provided. For cryo-EM structures, a representative micrograph showing individual particles should be provided in the figures alongside a processing workflow (please see our Editorial for more information: https://www.nature.com/articles/s41594-025-01567-9).

DATA DEPOSITION: We require deposition of coordinates (and, in the case of crystal structures, structure factors) into the Protein Data Bank with the designation of immediate release upon publication (HPUB). Electron microscopy-derived density maps and coordinate data must be deposited in EMDB and released upon publication. Deposition and immediate release of NMR chemical shift assignments are requested. To avoid delays in publication, dataset accession numbers must be supplied with the revised manuscript and appropriate release dates must be indicated at the galley proof stage.

3. REPORTING OF LIGHT MICROSCOPY DATA: For any revision that includes light microscopy data, we ask our authors to please include a completed light microscopy reporting table [https://www.nature.com/documents/Light_microscopy_reporting_table.xlsx] to ensure the methods are described thoroughly. The table will be available to reviewers and ultimately published, should the manuscript be accepted at the journal. When submitting the revised version of your manuscript, please pay close attention to our href="https://www.nature.com/nature-portfolio/editorial-policies/image-integrity">Digital Image Integrity Guidelines.

4. CODE AND COMPUTATIONAL WORK: If newly developed, unpublished code was used in this work, it must be provided with the revision and access to it must be disclosed in the Code Availability Statement in the manuscript. Please also provide the completed software submission checklist. This form is a dynamic ‘smart pdf’ and must be downloaded and completed in Adobe Reader, instead of opening it in a web browser: https://www.nature.com/documents/nr-software-policy.pdf

If molecular dynamics (MD) simulations were performed, please refer to this Editorial and provide a completed MD simulations checklist with your revision: https://www.nature.com/articles/s42003-023-04653-0

Lastly, if it applies, please complete and upload the completed machine learning checklist with your submission for re-review: https://www.nature.com/documents/machine-learning-checklist.pdf

5. SOURCE GEL IMAGES: Unprocessed scans should be provided for all gels and western blots presented in figures and should be clearly labelled with the figure number in the file title. The source gel images should be uncropped, unmodified images, with molecular weight markers. Uncropped, unmodified gel images from existing experimental repeats, in addition to the data shown in the figures, can also be presented in source data. Please provide these files as PDF with your revision.

Please ensure that all control panels for gels and western blots are appropriately described as loading or IP controls. Lastly, please ensure that all images in the paper are checked for duplication of panels and for splicing of gel lanes.

6. SOURCE NUMERICAL DATA: we urge authors to provide, in tabular form in excel files, the data underlying the graphical representations used in figures. This is to further increase transparency in data reporting, as detailed in this editorial (http://www.nature.com/nsmb/journal/v22/n10/full/nsmb.3110.html). Please provide one excel file per figure max, with one panel per tab. When submitting files, the title field should indicate which figure the source data pertains to.

7. DATA AVAILABILITY: this journal strongly supports public availability of data. All data used in accepted papers should be available via a public data repository, or alternatively, as Supplementary Information. If data can only be shared on request, please explain why in your Data Availability Statement and in the correspondence with your editor.

For some data types, deposition in a public repository is mandatory as detailed below:

https://www.nature.com/nature-research/editorial-policies/reporting-standards#availability-of-data

Finally, please ensure that you retain unprocessed data and metadata files after publication, ideally archiving data in perpetuity, as these may be requested during the peer review and production process or after publication if any issues arise.

EXTENDED DATA FIGURES

When re-submitting your manuscript, please ensure that any supplementary figures and tables that are crucial to the manuscript’s conclusions are converted into Extended Data figures and tables to increase visibility of these data. Extended Data figures and tables are online-only (present in the online PDF and full-text HTML versions of the paper), peer-reviewed display items that provide essential background to the article but are not included in the main article due to space constraints. A maximum of ten Extended Data display items (figures and tables) is permitted.

While we encourage the use of color in preparing figures, please note that this will incur a charge to partially defray the cost of printing. Information about color charges can be found at http://www.nature.com/nsmb/authors/submit/index.html#costs

Nature Structural & Molecular Biology is committed to improving transparency in authorship. As part of our efforts in this direction, we are now requesting that all authors identified as ‘corresponding author’ on published papers create and link their Open Researcher and Contributor Identifier (ORCID) with their account on the Manuscript Tracking System (MTS), prior to acceptance. This applies to primary research papers only. ORCID helps the scientific community achieve unambiguous attribution of all scholarly contributions. You can create and link your ORCID from the home page of the MTS by clicking on ‘Modify my Springer Nature account’. For more information please visit please visit www.springernature.com/orcid.

Please use the link below to submit your revised manuscript and related files:

Link Redacted

Note: This URL links to your confidential home page and associated information about manuscripts you may have submitted, or that you are reviewing for us. If you wish to forward this email to co-authors, please delete the link to your homepage.

We look forward to seeing the revised manuscript, thank you for the opportunity to review your work and once more our sincere apologies for the delay in returning an editorial decision.

Sincerely,

Dimitris Typas
Senior Editor
Nature Structural & Molecular Biology
ORCID: 0000-0002-8737-1319

Reviewer #1 (Remarks to the Author):

This is a strong and technically impressive manuscript that addresses an important and longstanding question in transcription biology: how a transcription factor efficiently locates its cognate sites within chromatin while avoiding extensive off-target trapping. The experimental strategy is particularly elegant. The combination of purified native yeast minichromosomes, single-molecule TIRF measurements, locus mutagenesis at the HIS3 promoter, and complementary live-cell SMT provides a powerful framework for dissecting the search process. The work is conceptually clear, carefully executed, and the conclusions are well supported by the data.The use of a gene-sized native chromatin template is a major strength and allows the authors to examine TF search behavior in a more realistic genomic context. The comparison between naked genomic DNA and chromatin is also very convincing and clearly shows that naked DNA promotes extensive irregular trapping of GCN4, whereas nucleosome organization suppresses this promiscuity and effectively confines productive search to NFRs. The authors observe that the GCN4 activation domain/IDR accelerates both association and dissociation kinetics - thereby improving targeting efficiency -is quite compelling and will likely stimulate further work on the kinetic roles of IDRs in transcription factor function.

This is an elegant single-molecule study that provides important new insight into how chromatin organization shapes transcription factor target search. The experiments are carefully designed, the data are convincing, and the conceptual advance will be of broad interest to the chromatin and gene regulation communities. I am highly enthusiastic about publication after minor revision.

1. The long-linker nucleosome experiment provides a useful reductionist test of the model. The authors note that some free DNA is present in the nucleosome preparation (Fig. 3e/f). If not already quantified, it would be helpful to provide an estimate of the free DNA fraction or briefly explain why this does not substantially affect the interpretation of the experiment.

2. One minor point from a single-molecule perspective: because the in vitro TIRF movies are 600 s long and the longest bin includes events >600 s, it may be worth adding one sentence clarifying how right-censoring of very long events was handled in the residence-time analysis, particularly for the most stable ccDNA-bound population. I do not think this affects the main conclusions, but a brief clarification would help readers interpret the absolute values of the longest-lived component.

3. The data strongly support the idea that nucleosomes suppress off-target trapping by restricting the effective search space to NFR DNA. The authors may consider being even more careful about wording the chromatin results as direct evidence for 1D scanning on the minichromosome, since what is measured here is a kinetic signature that is consistent with a roadblock model rather than direct tracking of sliding itself.

Reviewer #2 (Remarks to the Author):

The manuscript by Wei et al reports how nucleosome organization and IDRs might help GCN4 binding to its specific binding site. The single molecule TIRF imaging method on bare DNA and mini-chromosomes is elegant and seem to yield high quality data to address this question. There are, however, several major issues that need to be addressed before suitability for publication of this manuscript can be determined.

1) The errors and statistics of their findings are not well reported. In the text and figures many numbers are presented and discussed without much regards of the statistical significance. This includes not mentioning the sampling sizes, the nature and the value of the errors -SD or SE-. This is a major weakness and needs to be fixed. Moreover, the discussion/conclusions drawn should be done with these errors/confidence intervals taken into account. It might actually change what can and can't be concluded.
2) The representation of their off-rates/residence times is a bit unusual (Fig 2a and others). The authors indicate that there are different 'binding populations' of GCN4, this is typically concluded from life-time binding plots that show that multiple exponentials can be fitted to the data. The authors should consider doing this as well and as such prove more solidly that 3 population indeed are present. In fact, the line 114 of the text seems to suggest that exponentials were fitted (but not shown).
3) From the data the authors conclude that GCN4 does 1D sliding on the bare DNA. The possibility of hopping (very short distance dissociation and association) is not considered. There is a vast amount of literature discussing the search dynamics of proteins on DNA and it might be insightful to consider more of what has been reported in the past about this. For example, one key parameter that is often varied is the salt concentration which impacts 1D, 3D search dynamics in different ways. The author should consider investigating that as well.
4) The paragraph starting from line 183 is quite speculative and not firmly based on data/observation reported here. The content should be revised, toned down and/or partly moved to the discussion.
5) The report investigates GCN4 and the results are thus valid for that system. A more generic statement about how TF search their target sides on chromatin is not warranted without showing similar results on at least one other protein.

The manuscript is full of abbreviations which makes reading it much harder. I would suggest the authors to critical assess if they can reduce their use of abbreviation.

In many places the order of the panels discussed is different than the order in which they are shown in the figures. This is confusing and should be fixed.

Version 1:

Decision Letter:

Our ref: NSMB-A52440A

5th Jun 2026

Dear Professor Wu,

Thank you for submitting your revised manuscript "Nucleosomes and TF-IDRs facilitate target search by suppressing TF promiscuity on minichromosome" (NSMB-A52440A). I apologise for the delay in returning our editorial decision as we faced difficulty in timely obtaining and discussing reviewers' reports. Nevertheless, the revised manuscript has now been seen by the two original referees that had provided feedback and their comments are below. The reviewers find that the paper has improved in revision and retain no concerns. Therefore we can now accept it in principle for publication in Nature Structural & Molecular Biology, pending minor revisions to comply with our editorial and formatting guidelines.

We are now performing detailed checks on your paper and will send you a checklist detailing our editorial and formatting requirements in 1-2 weeks. Please do not upload the final materials and make any revisions until you receive this additional information from us.

To facilitate our work at this stage, it is important that we have a copy of the main text as a word file. If you could please send along a word version of this file as soon as possible, we would greatly appreciate it; please make sure to copy the NSMB account (cc'ed above).

Thank you again for your interest in Nature Structural & Molecular Biology Please do not hesitate to contact me if you have any questions.

Sincerely,

Dimitris Typas
Senior Editor
Nature Structural & Molecular Biology
ORCID: 0000-0002-8737-1319

Reviewer #1 (Remarks to the Author):

The authors have done an exemplary job of addressing all reviewers' comments, and I most enthusiastically recommend publication of the manuscript.

Reviewer #2 (Remarks to the Author):

The authors did a nice job revising the manuscript and all the issues that I raised have been addressed.It can now be considered for publication.

Version 2:

Decision Letter:

14th Jul 2026

Dear Professor Wu,

We are now happy to accept your revised paper "Nucleosomes and IDRs suppress promiscuous GCN4 binding on minichromosomes" for publication as an Article in Nature Structural & Molecular Biology.

Acceptance is conditional on the manuscript's not being published elsewhere and on there being no announcement of this work to the newspapers, magazines, radio or television until the publication date in Nature Structural & Molecular Biology.

Over the next few weeks, your paper will be copyedited to ensure that it conforms to Nature Structural & Molecular Biology style. Once your paper is typeset, you will receive an email with a link to choose the appropriate publishing options for your paper and our Author Services team will be in touch regarding any additional information that may be required.

After the grant of rights is completed, you will receive a link to your electronic proof via email with a request to make any corrections within 48 hours. If, when you receive your proof, you cannot meet this deadline, please inform us at rjsproduction@springernature.com immediately.

You will not receive your proofs until the publishing agreement has been received through our system.

Due to the importance of these deadlines, we ask that you please let us know now whether you will be difficult to contact over the next month. If this is the case, we ask you provide us with the contact information (email, phone and fax) of someone who will be able to check the proofs on your behalf, and who will be available to address any last-minute problems.

To assist our authors in disseminating their research to the broader community, our SharedIt initiative provides all co-authors with the ability to generate a unique shareable link that will allow anyone (with or without a subscription) to read the published article. Recipients of the link with a subscription will also be able to download and print the PDF.

As soon as your article is published, you can generate your shareable link by entering the DOI of your article here: http://authors.springernature.com/share. Corresponding authors will also receive an automated email with the shareable link

Note the policy of the journal on data deposition: http://www.nature.com/authors/policies/availability.html.

Your paper will be published online soon after we receive proof corrections and will appear in print in the next available issue. You can find out your date of online publication by contacting the production team shortly after sending your proof corrections.

You may wish to make your media relations office aware of your accepted publication, in case they consider it appropriate to organize some internal or external publicity. Once your paper has been scheduled you will receive an email confirming the publication details. This is normally 3-4 working days in advance of publication. If you need additional notice of the date and time of publication, please let the production team know when you receive the proof of your article to ensure there is sufficient time to coordinate. Further information on our embargo policies can be found here: https://www.nature.com/authors/policies/embargo.html

You can now use a single sign-on for all your accounts, view the status of all your manuscript submissions and reviews, access usage statistics for your published articles and download a record of your refereeing activity for the Nature journals.

If you have not already done so, we strongly recommend that you upload the step-by-step protocols used in this manuscript to the Protocol Exchange. Protocol Exchange is an open online resource that allows researchers to share their detailed

experimental know-how. All uploaded protocols are made freely available, assigned DOIs for ease of citation and fully searchable through nature.com. Protocols can be linked to any publications in which they are used and will be linked to from your article. You can also establish a dedicated page to collect all your lab Protocols. By uploading your Protocols to Protocol Exchange, you are enabling researchers to more readily reproduce or adapt the methodology you use, as well as increasing the visibility of your protocols and papers. Upload your Protocols at www.nature.com/protocolexchange/. Further information can be found at www.nature.com/protocolexchange/about.

An online order form for reprints of your paper is available at https://www.nature.com/reprints/author-reprints.html. Please let your coauthors and your institutions' public affairs office know that they are also welcome to order reprints by this method.

Authors may need to take specific actions to achieve compliance with funder and institutional open access mandates. If your research is supported by a funder that requires immediate open access (e.g. according to Plan S principles or the NIH public access policy) then you should select the gold OA route, and we will direct you to the compliant route where possible. Because authors warrant under our subscription licensing terms that they haven't committed to licensing any version of their article under a licence inconsistent with the terms of our agreement – including the applicable embargo period – publication under the subscription model isn't suitable for authors whose funders require no embargo.

In approximately 10 business days you will receive an email with a link to choose the appropriate publishing options for your paper and our Author Services team will be in touch regarding any additional information that may be required.

You will not receive your proofs until the publishing agreement has been received through our system.

If you have any questions about our publishing options, costs, Open Access requirements, or our legal forms, please contact ASJournals@springernature.com

Sincerely,

Dimitris Typas
Senior Editor
Nature Structural & Molecular Biology
ORCID: 0000-0002-8737-1319

Click here if you would like to recommend Nature Structural & Molecular Biology to your librarian: http://www.nature.com/subscriptions/recommend.html#forms

Open Access This Peer Review File is licensed under a Creative Commons Attribution 4.0 International License, which permits use, sharing, adaptation, distribution and reproduction in any medium or format, as long as you give appropriate

credit to the original author(s) and the source, provide a link to the Creative Commons license, and indicate if changes were made.
In cases where reviewers are anonymous, credit should be given to 'Anonymous Referee' and the source.
The images or other third party material in this Peer Review File are included in the article's Creative Commons license, unless indicated otherwise in a credit line to the material. If material is not included in the article's Creative Commons license and your intended use is not permitted by statutory regulation or exceeds the permitted use, you will need to obtain permission directly from the copyright holder.
To view a copy of this license, visit https://creativecommons.org/licenses/by/4.0/

Dear Dimitris,

We greatly appreciate your editorial efforts in coordinating the review of our paper and we also thank the reviewers for their time and valuable comments which have substantially helped us improve the work.

In response to their comments, we have conducted additional experiments and modified the text and figures to strengthen our conclusions. We trust the revised manuscript meets reviewers' expectations for acceptance by the journal.

Below please find a point-by-point reply to the critique.

Reviewer #1 (Remarks to the Author):

This is a strong and technically impressive manuscript that addresses an important and longstanding question in transcription biology: how a transcription factor efficiently locates its cognate sites within chromatin while avoiding extensive off-target trapping. The experimental strategy is particularly elegant. The combination of purified native yeast minichromosomes, single-molecule TIRF measurements, locus mutagenesis at the HIS3 promoter, and complementary live-cell SMT provides a powerful framework for dissecting the search process. The work is conceptually clear, carefully executed, and the conclusions are well supported by the data. The use of a gene-sized native chromatin template is a major strength and allows the authors to examine TF search behavior in a more realistic genomic context. The comparison between naked genomic DNA and chromatin is also very convincing and clearly shows that naked DNA promotes extensive irregular trapping of GCN4, whereas nucleosome organization suppresses this promiscuity and effectively confines productive search to NFRs. The authors observe that the GCN4 activation domain/IDR accelerates both association and dissociation kinetics - thereby improving targeting efficiency -is quite compelling and will likely stimulate further work on the kinetic roles of IDRs in transcription factor function.

This is an elegant single-molecule study that provides important new insight into how chromatin organization shapes transcription factor target search. The experiments are carefully designed, the data are convincing, and the conceptual advance will be of broad interest to the chromatin and gene regulation communities. I am highly enthusiastic about publication after minor revision.

1. The long-linker nucleosome experiment provides a useful reductionist test of the model. The authors note that some free DNA is present in the nucleosome preparation (Fig. 3e/f). If not already quantified, it would be helpful to provide an estimate of the free DNA fraction or briefly explain why this does not substantially affect the interpretation of the experiment.

Thank you for pointing out this technical limitation of our long-linker nucleosome experiments. Indeed, the residence time we measured in the original experiment would be a mixture of GCN4 binding on long linker nucleosomes and free DNA (quantification: 76% nuc. versus 24% DNA for WT and 59% versus 41% for UAS^{Δ}). To strengthen our conclusion, we repeated this experiment using nucleosomes with Cy5-labeled H2B (a generous gift from Greg D. Bowman lab) and AF488-labeled DNA, which allows detection of GCN4 binding events on the long linker nucleosomes only (by selecting for spots with both AF488 (DNA) and Cy5 (H2B) signals). We have replaced the original Fig. 3e-3g as follows:

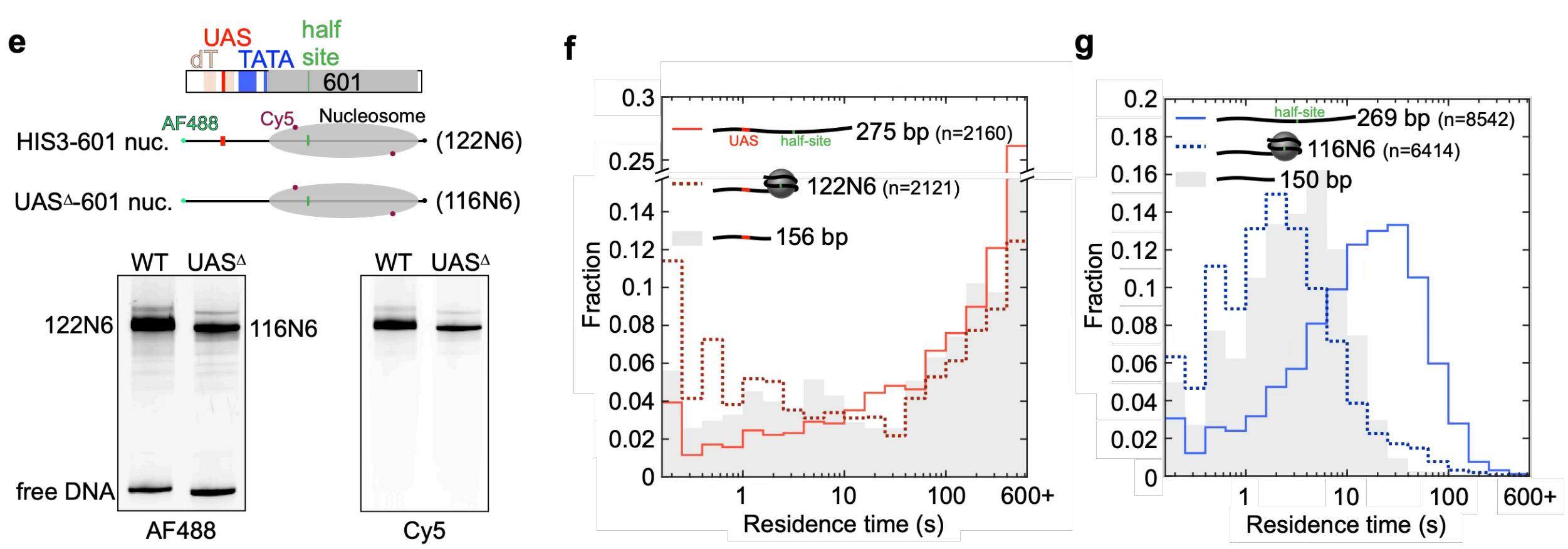

We also add exponential fitting results and statistics for these experiments into Extended Data Fig. 3:

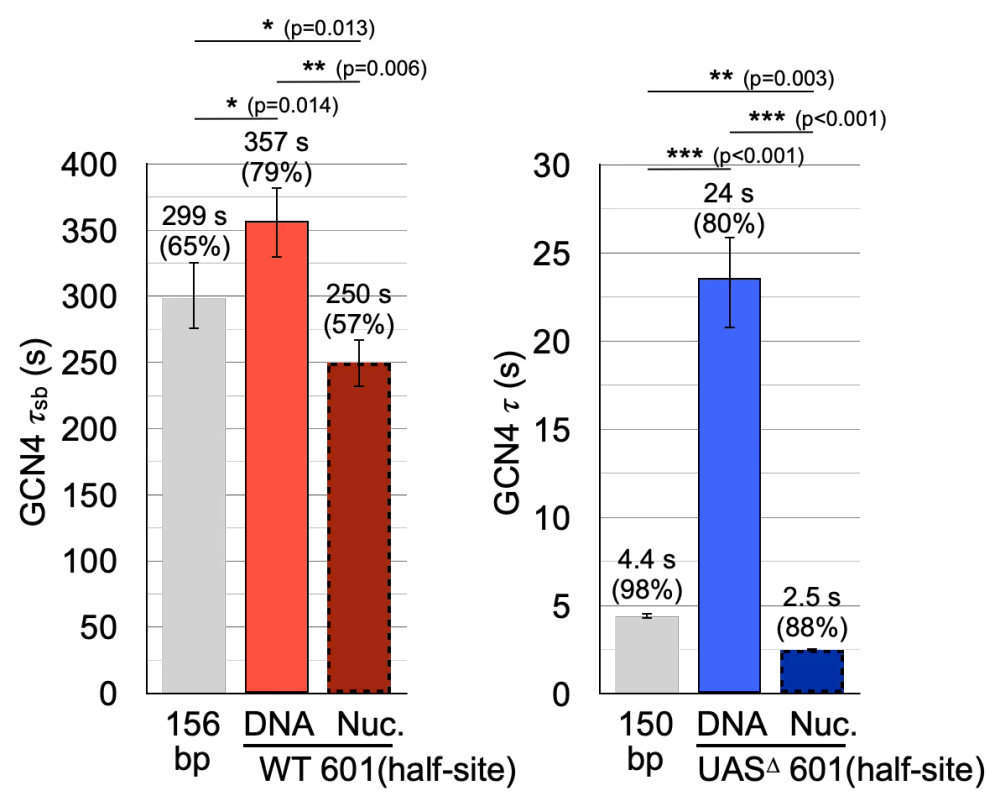

Our new experiments notably eliminate interference by contaminating free DNA and show that nucleosome organization substantially reduces GCN4 τ (please see modified text line 166-178).

Moreover, the same analysis on UAS$^{\Delta}$ 116N6 **without** half-site also reveals a reduction of GCN4 τ (new Extended Data Fig. 3i).

2. One minor point from a single-molecule perspective: because the in vitro TIRF movies are 600 s long and the longest bin includes events >600 s, it may be worth adding one sentence clarifying how right-censoring of very long events was handled in the residence-time analysis, particularly for the most stable ccDNA-bound population. I do not think this affects the main conclusions, but a brief clarification would help readers interpret the absolute values of the longest-lived component.

Thank you for pointing out right-censored events. In our 'Residence time calculations and multi-component exponential fitting' section under 'Data analysis and quantification', we briefly mentioned how we handle censored events and we agree that we should clarify better as follows:

Left-censored events are treated as non-censored events given the memorylessness of the exponential distribution. We also include events that last throughout the entire 600-s video (double-censored events), but the right-censored events are discarded during the fitting because of the high variance they introduce (see Methods).

We have added a clarification noting the under-estimation of stable binding on WT ccDNA due to right-censored event exclusion in the main text (line 118-120: 'F_{sb} and τ_{sb} for WT

ccDNA could be under-estimated due to partial exclusion of right-censored binding events, see Methods').

3. The data strongly support the idea that nucleosomes suppress off-target trapping by restricting the effective search space to NFR DNA. The authors may consider being even more careful about wording the chromatin results as direct evidence for 1D scanning on the minichromosome, since what is measured here is a kinetic signature that is consistent with a roadblock model rather than direct tracking of sliding itself.

The reviewer is completely correct. To address this issue, we have performed a new 3-color FRET experiment (Marklund, *et al.* Nature, 2020; Feng, *et al*. NSMB, 2025) and now show below that GCN4 can undergo 1D diffusion by direct observations (please see additional details in new Extended Data Fig. 7):

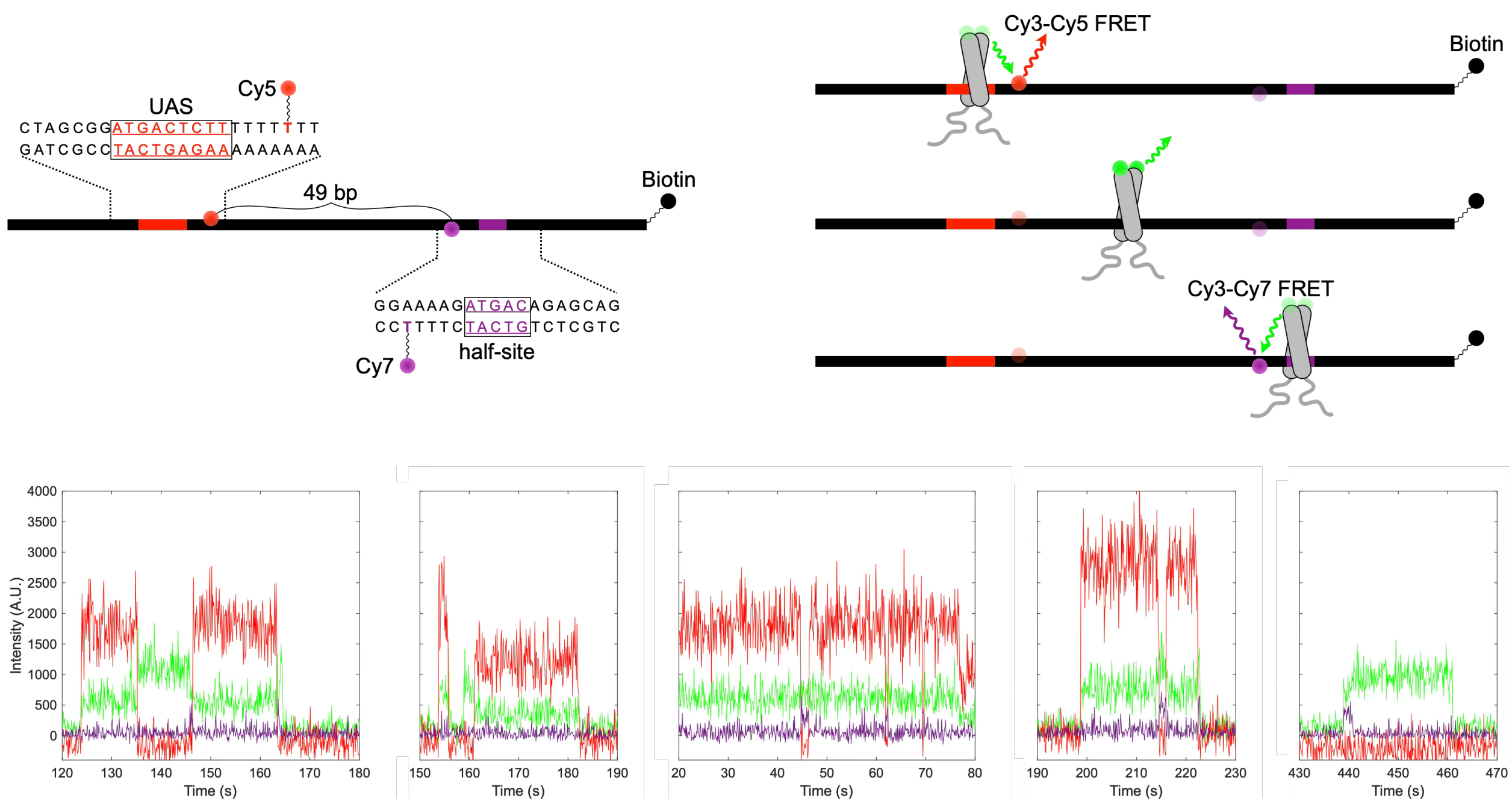

We also added the corresponding descriptions into the main text (line 135).

Reviewer #2 (Remarks to the Author):

The manuscript by Wei et al reports how nucleosome organization and IDRs might help GCN4 binding to its specific binding site. The single molecule TIRF imaging method on bare DNA and mini-chromosomes is elegant and seem to yield high quality data to address this question. There are, however, several major issues that need to be addressed before suitability for publication of this manuscript can be determined.

1) The errors and statistics of their findings are not well reported. In the text and figures many numbers are presented and discussed without much regards of the statistical significance. This includes not mentioning the sampling sizes, the nature and the value of the errors -SD or SE-. This is a major weakness and needs to be fixed. Moreover, the discussion/conclusions drawn should be done with these errors/confidence intervals taken into account. It might actually change what can and can't be concluded.

We appreciate the request for rigorous statistics. For improvements, we have made these revisions:

1. In addition to our original descriptions on sample sizes in Methods, we now label n values in Fig. 2, 3, part of 4, 5, Extended Data Fig. 2-5. For a few panels lacking space (Fig. 4b and 4c, Extended Data Fig. 5g), we note where n values can be found. We also add sample sizes for key experiments in the main text.

2. Regarding standard deviation (S.D.) and 95% confidence intervals (95% C.I.), we upload three extra tables (MCandccDNA_fitting.csv, linearDNAandnuc_fitting.csv, and kon_fitting.csv) in supplementary files including exact values of S.D. or 95% C.I. for all data presented in our work.

3. Regarding statistical confidence of conclusions, we now add p-values in brackets in the main text for major conclusions we have drawn, in addition to p-values provided in main Figs and Extended Data Figs.

2) The representation of their off-rates/residence times is a bit unusual (Fig 2a and others). The authors indicate that there are different 'binding populations' of GCN4, this is typically concluded from life-time binding plots that show that multiple exponentials can be fitted to the data. The authors should consider doing this as well and as such prove more solidly

that 3 population indeed are present. In fact, the line 114 of the text seems to suggest that exponentials were fitted (but not shown).

We appreciate the request regarding data presentation and fitting methods, and have added 1-CDF plots with exponential fitting to Fig. 2 and 3. We wish to keep the original histograms to show more details of the residence time distributions.

Regarding fitting methods, we used standard multi-component exponential fitting for our residence time measurements with details included in Methods.

Regarding the number of components (populations), here are our reasoning for using 3-component exponential fitting:

1. For all data, we tested 1- to 5-component exponential fitting, and used Bayesian information criterion (BIC) to decide the number of components. For example, for a typical dataset ‘WTG0909’, the BIC values for 1- to 5- component exponential fitting are:

1-component	2-component	3-component	4-component	5-component
Fitting failed	9968.39915530984	9914.61664437564	9921.61336544550	9935.14491286002

The BIC for 3-component fitting is the lowest. Therefore, we conventionally use 3-component exponential fitting.

2. The results from 1-component to 5-component fitting for the above dataset show that, the fraction values for 3 major populations barely change above 3-component fitting.

	**Fraction 1**	**k1**	**Fraction 2**	**k2**	**Fraction 3**	**k3**	**Fraction 4**	**k4**	**Fraction 5**	**k5**
**1-comp**	failed									
**2-comp**	0.7227	0.2093	0.2773	0.0191						
**3-comp**	0.4296	0.4061	0.4874	0.0642	0.0830	0.0088				
**4-comp**	0.4018	0.4324	0.4838	0.0725	0.1115	0.0128	0.0029	0.0004		
**5-comp**	0.4062	0.4267	0.4854	0.0709	0.1059	0.0123	0.0018	0.0002	0.0007	1.52e-11

3. 2-component fitting clearly misses the tail starting at 100 s (left panel is 2-component fitting and right is 3-component):

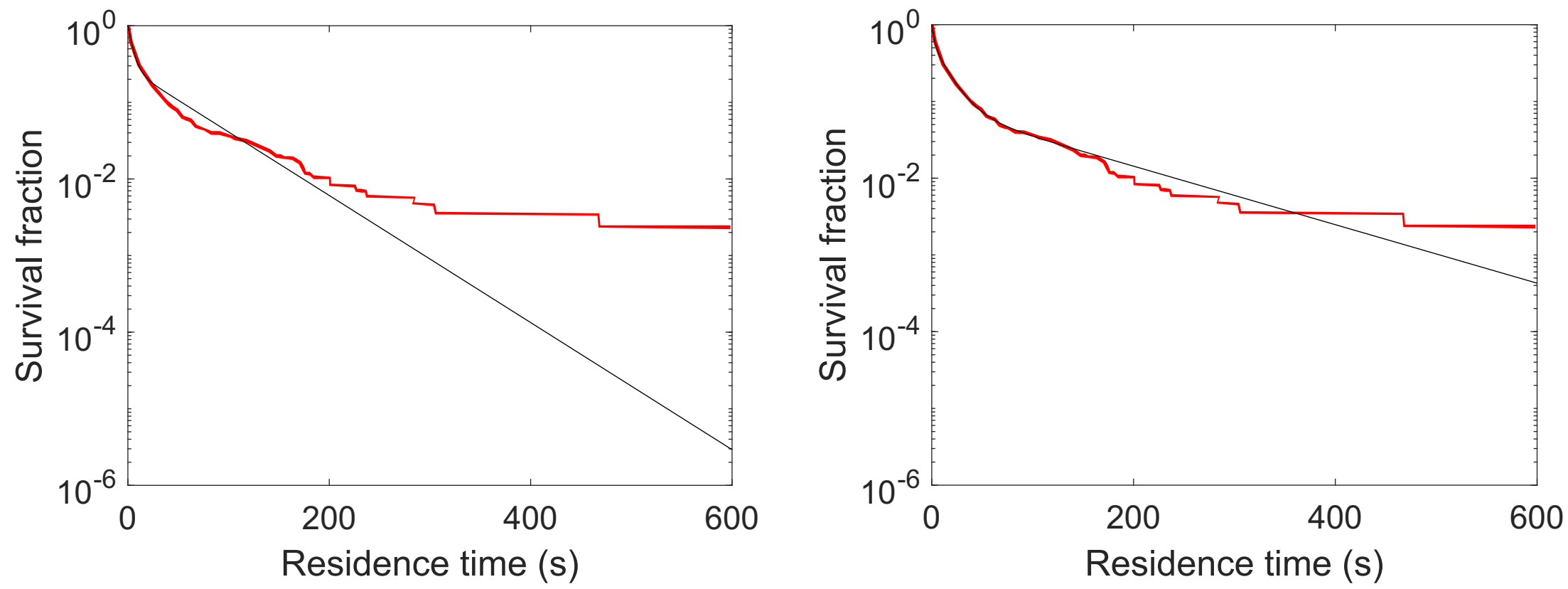

Altogether, we conclude that 3-component exponential fitting is optimal.

3) From the data the authors conclude that GCN4 does 1D sliding on the bare DNA. The possibility of hopping (very short distance dissociation and association) is not considered. There is a vast amount of literature discussing the search dynamics of proteins on DNA and it might be insightful to consider more of what has been reported in the past about this. For example, one key parameter that is often varied is the salt concentration which impacts 1D, 3D search dynamics in different ways. The author should consider investigating that as well.

We agree this is an important point, and now provide a 3-color FRET experiment data (Marklund, *et al.* Nature, 2020; Feng, *et al*. NSMB, 2025) to directly observe GCN4 1D diffusion on linear DNA in different salt concentrations:

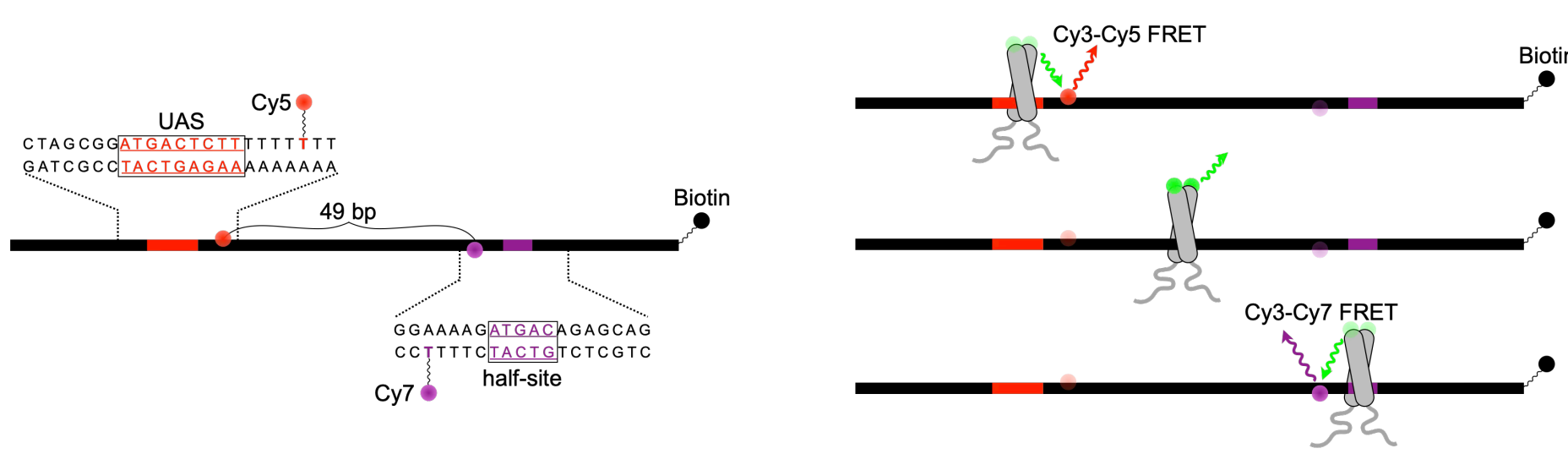

When K^+ concentration is 175 mM, only 7.6% of the traces show 1D diffusion:

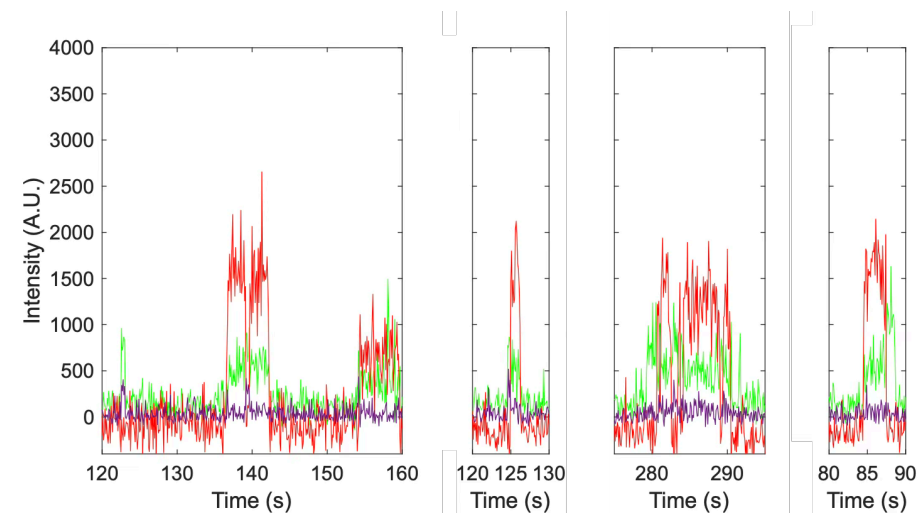

Under our standard conditions (150 mM KOAc), 21.6% of traces show GCN4 1D diffusing between UAS, half-site and off-target DNA sequences:

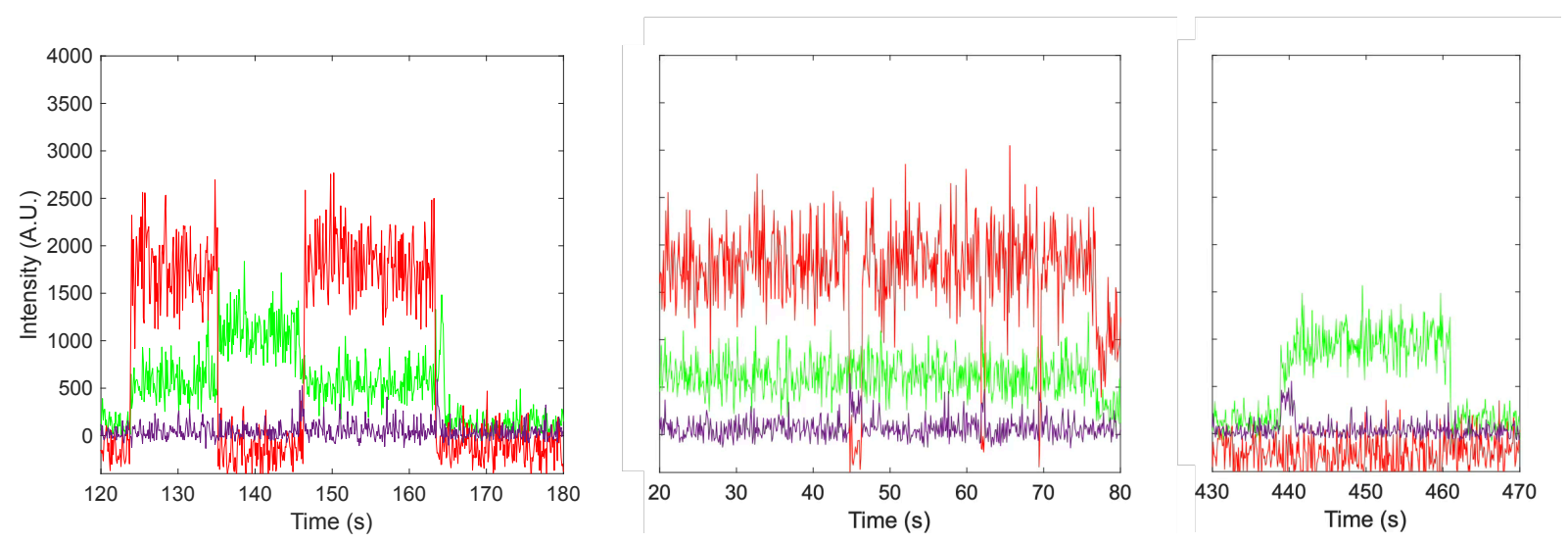

When dropping K^+ concentration to 100 mM, 41.9% of the traces show 1D diffusion:

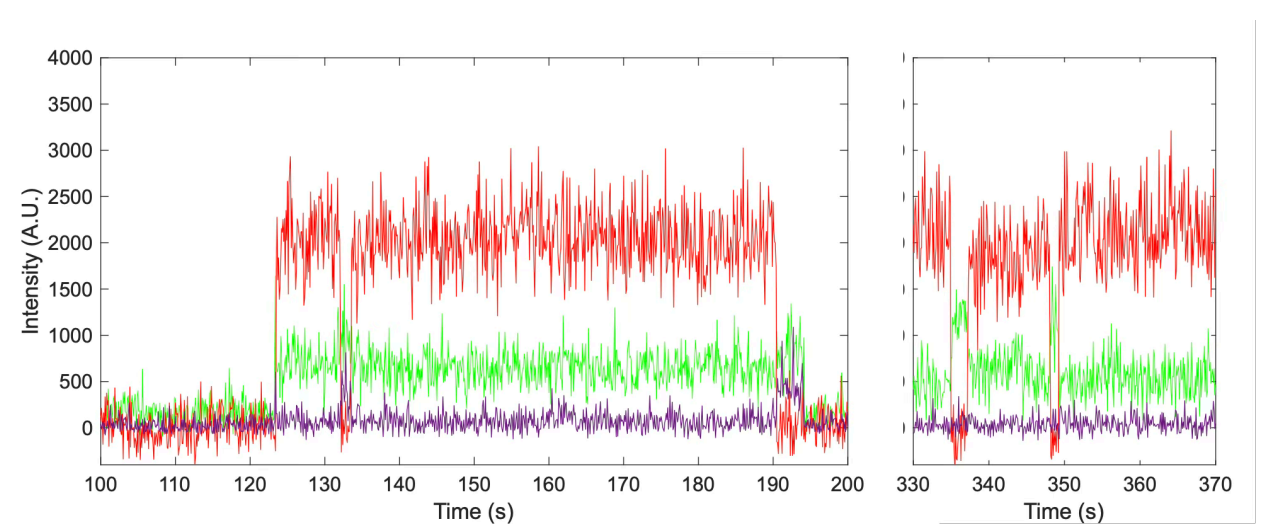

Such salt sensitivity is consistent with other reports for a role of TF hopping in 1D. We have organized these data into a new Extended Data Fig. and added the corresponding descriptions in the main text.

4) The paragraph starting from line 183 is quite speculative and not firmly based on data/observation reported here. The content should be revised, toned down and/or partly moved to the discussion.

We agree and have revised the statements as follows:

1. Original: “Unlike general k_{on}, the $k_{on,sb}$ reaches a plateau…indicating **a 1D diffusion distance between 103 bp and 233 bp.**”

Revised: …suggesting that $k_{on,sb}$ is limited by the distance range of 1D diffusion before entrapment or dissociation.

(We also cite Sakong, *et al.* Nat. Commun. 2026 where they mathematically modeled the sliding length of TFs based on similar observations).

2. Original: "For ccDNA, $k_{on,sb}$ apparently exceeds this limit, **likely due to the supercoiled conformation** (Extended Data Fig. 1b) **favoring DNA proximity and therefore assisting GCN4 transfer to the UAS in 3D space**."

Revised: "Of interest, $k_{on,sb}$ apparently exceeds this limit for ccDNA (see Discussion)."

In discussion: "It is interesting that $k_{on,sb}$ for ccDNA is substantially exceeds the upper limit for short linear fragments. Besides the inclusion of stable, off-target binding events, we speculate that the elevated $k_{on,sb}$ could be due to the higher spatial DNA density of a supercoiled template (Extended Data Fig. 1b). Futures studies on supercoiled and relaxed ccDNA and MC should provide insights on the influence of DNA topoisomerases on the kinetics of TF target search."

5) The report investigates GCN4 and the results are thus valid for that system. A more generic statement about how TF search their target sides on chromatin is not warranted without showing similar results on at least one other protein.

We are unclear on which generic statement regarding GCN4 target search is being questioned by the reviewer. We have cited other eukaryotic TFs that show similar nucleosome restriction of 1D target search (GAGA factor, Feng, *et al.* NSMB, 2025) or display 1D diffusion behaviors (Sox2, J. Chen, *et al.* Cell, 2014, Sakong, *et al.* Nat Commun. 2026; p53, Leith, *et al*, PNAS, 2012).

For TF AD functions, we cited TFs with similar AD/IDR-facilitated dissociation/association behaviors (Chen, *et al.* Cell, 2014; Krois, *et al.* PNAS, 2018; He, *et al.* PNAS, 2019; Schutz, *et al.* Biochemistry, 2024) but also TFs showing different functions (**T. Nguyen,** *et al.* Nat. Commun. 2022; **A. A. Abidi**, *et al.* Science, 2026; **N. Strugo**, *et al.* Nat. Commun. 2025). It is not our intent to state that the GCN4 AD function is generalizable to all TFs; indeed, our AD substitution data do not support full generalization (Discussion).

We are prepared to consider modifying specific statements in the text that the reviewer identifies as not warranted by the presented data. The word ‘generalized’ appears once in the manuscript (Discussion: “These effects might be generalized to other TF ADs or IDRs, as substitution of VP16 AD…”) and we have changed the wording to ‘These effects are observed for other TFs…’

The manuscript is full of abbreviations which makes reading it much harder. I would suggest the authors to critical assess if they can reduce their use of abbreviation.

We now directly use WT 122N6 (half-site), UAS^{Δ} 116N6 (half-site), or UAS^{Δ} 116N6 (no half-site) to describe nucleosomes instead of using asterisks to denote the inserted half-site within 601.

We also omit the asterisk in $GCN4^{FL}$*-Halo denoting PEST mutant, which is described in the text.

In many places the order of the panels discussed is different than the order in which they are shown in the figures. This is confusing and should be fixed.

Thank you for pointing this out. We re-ordered Fig. 2 to fit the logic of the main text. We also adjusted some panels in Fig. 3, Extended Data Fig. 3, switched Extended Data Fig. 6 and 7, re-ordered Extended Data Fig. 7, and added one Extended Data Fig. to make reading smoother.

Reviewer #1:

Remarks to the Author:

The authors have done an exemplary job of addressing all reviewers' comments, and I most enthusiastically recommend publication of the manuscript.

Reviewer #2:

Remarks to the Author:

The authors did a nice job revising the manuscript and all the issues that I raised have been addressed.It can now be considered for publication.

We sincerely appreciate the time and effort of the reviewers. Their remarks have greatly improved our manuscript.
