# Supplementary material for: Nucleosomes and IDRs suppress promiscuous GCN4 binding on minichromosomes
Source: Nat Struct Mol Biol. 2026 Aug 13;33(9):1332–40. doi: 10.1038/s41594-026-01864-x (PMC13561865; doi:10.1038/s41594-026-01864-x)

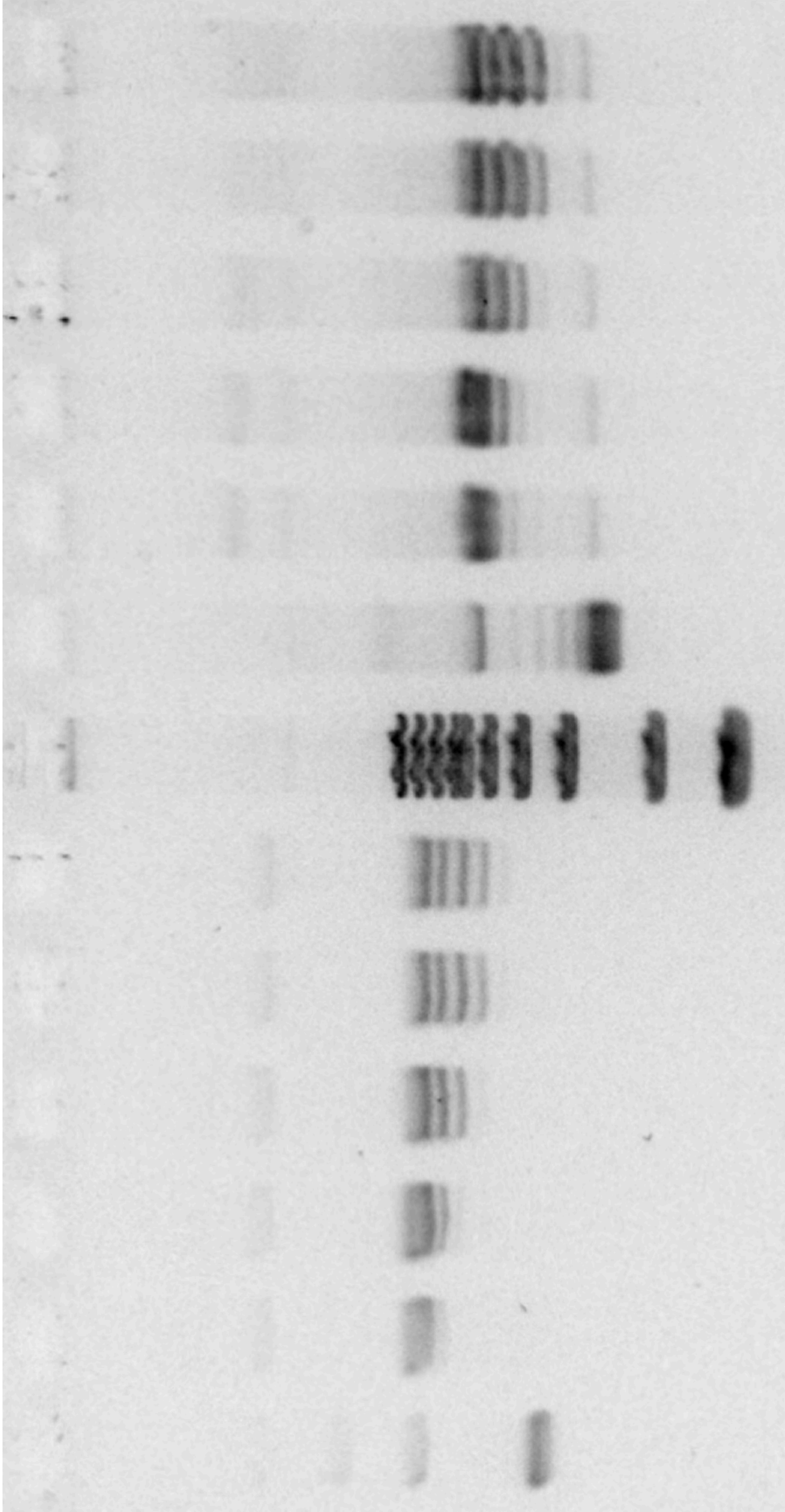

Supplement: Supplementary file 4 [file 41594_2026_1864_MOESM8_ESM.pdf]
